# A synthetic, catalytic and theoretical investigation of an unsymmetrical SCN pincer palladacycle

**DOI:** 10.1098/rsos.150656

**Published:** 2016-04-06

**Authors:** Gavin W. Roffe, Sarote Boonseng, Christine B. Baltus, Simon J. Coles, Iain J. Day, Rhiannon N. Jones, Neil J. Press, Mario Ruiz, Graham J. Tizzard, Hazel Cox, John Spencer

**Affiliations:** 1 Department of Chemistry, School of Life Sciences, University of Sussex, Falmer, Brighton BN1 9QJ, UK; 2 School of Science, University of Greenwich at Medway, University of Greenwich, Chatham ME4 4TB, UK; 3 UK National Crystallography Service, School of Chemistry, University of Southampton, Highfield, Southampton SO17 1BJ, UK; 4 Novartis Pharmaceuticals UK Ltd, Horsham RH12 5AB, UK

**Keywords:** palladium, C–H activation, calculations

## Abstract

The SCN ligand 2-{3-[(methylsulfanyl)methyl]phenyl}pyridine, 1, has been synthesized starting from an initial Suzuki–Miyaura (SM) coupling between 3-((hydroxymethyl)phenyl)boronic acid and 2-bromopyridine. The C–H activation of 1 with *in situ* formed Pd(MeCN)_4_(BF_4_)_2_ has been studied and leads to a mixture of palladacycles, which were characterized by X-ray crystallography. The monomeric palladacycle LPdCl 6, where L-H = 1, has been synthesized, and tested in SM couplings of aryl bromides, where it showed moderate activity. Density functional theory and the atoms in molecules (AIM) method have been used to investigate the formation and bonding of 6, revealing a difference in the nature of the Pd–S and Pd–N bonds. It was found that S-coordination to the metal in the rate determining C–H bond activation step leads to better stabilization of the Pd(II) centre (by 13–28 kJ mol^−1^) than with N-coordination. This is attributed to the electron donating ability of the donor atoms determined by Bader charges. The AIM analysis also revealed that the Pd–N bonds are stronger than the Pd–S bonds influencing the stability of key intermediates in the palladacycle formation reaction pathway.

## 1. Introduction

**Figure 1.**
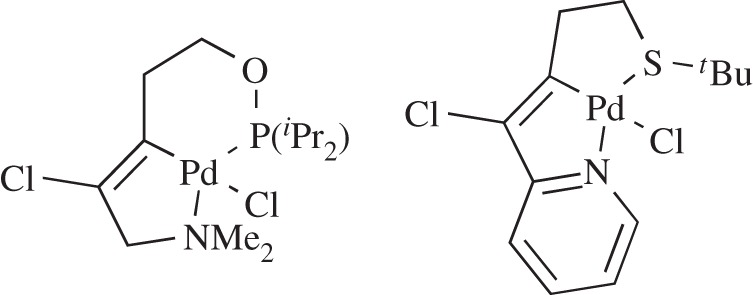
Unsymmetrical PCN [10] and SCN [11] palladacycles.

Since its inception, by Cope & Siekman, in 1965 [1], the chemistry of palladacycles has grown into a popular area of research. The seminal discovery by Herrmann and Beller that palladacycles are efficient catalysts for C–C bond formation [2,3] paved the way for a number of reports on catalytic applications, with several reviews and an excellent book covering the wide array of examples [4–7]. Pincer complexes, where the palladium–carbon bond is stabilized intramolecularly by two donor atoms, are another interesting subtype of palladacycles [8,9]. Although the majority are symmetrical [6], a limited number of unsymmetrical complexes have been reported, often synthesized by more challenging routes than their symmetrical analogues. Examples of interesting unsymmetrical pincers include those synthesized by Dupont and co-workers [10,11] (figure 1), by Fleckhaus *et al.* [12] and by Milstein and co-workers [13,14] on late transition metal unsymmetrical metallacycles. Other interesting pincers include ferrocene-based SCN palladacycles that were used in catalytic allylic alkylations [15].

A number of reports have been published showing unsymmetrical pincer palladacycles to be more active in various catalytic applications than their symmetrical counterparts [16,17].

The Suzuki–Miyaura (SM) coupling reaction [18,19] has widespread use in pharmaceutical research and academia. Recent examples using pincer palladacycles include the coupling of activated and deactivated bromides using SCN pincers, which also included use of the Hg drop test as evidence of the palladacycles acting as a source of catalytically active Pd(0) [20]. Thioether-based palladacycles have also been shown to form catalytically active Pd nanoparticles by transmission electron microscopy in SM couplings [21]. A range of activated and deactivated aryl bromides were coupled using a thioether-functionalized iminophosphorane SCN palladacycle [22]. Aryl bromides and aryl chlorides were also successfully coupled with NCN [23,24] and PCP [25] palladacycles. Therefore, owing to the number of reports of the application of pincer palladacycles in SM coupling, this reaction is considered to be a suitable benchmark for the study of new palladacycle catalysts, despite often being surpassed in performance by other palladium-based catalysts [19,26].

Previously described syntheses of unsymmetrical pincers are often low yielding. For example, attempts to desymmetrize 1,3-bis(bromomethyl)benzenes with different P- and S-based nucleophiles, gave the desired unsymmetrical product with concomitant formation of symmetrical bis-S-,S- and bis-P-,P-substituted products [17]. Applications in tandem catalysis and evidence that unsymmetrical pincers may provide opportunities to fine-tune catalytic activity encouraged us to investigate a more robust synthetic strategy, which would provide an easy route to a large number of new interesting ligands and unsymmetrical SCN pincer palladacycles and their applications in catalysis.

Recently, we showed that the strength and nature of the bonding in symmetrical palladacycles can have an effect on the energetics of a model formation reaction:

$$\text{LH}+{\text{PdCl}}_{2}\to \text{PdLCl}+\text{HCl},$$

studied using density functional theory (DFT). It was shown that the thermodynamic stability and the energy barriers for the key C–H bond activation step in the formation of symmetrical palladacycles were dependent on the pincer ligand donor atoms. The PCP palladacycle was found to have the smallest C–H activation barrier, SCS intermediate and NCN the largest barrier [27]. Work by other groups has included investigations of various mechanisms for C–H bond activation in cyclometallation [28–31].

The purposes of this paper are threefold. Firstly, to devise a robust synthesis for the formation of a novel SCN palladacycle that allows an easy route for future late-stage diversification, through modification of the sulfur substituent, as a simpler route to interesting unsymmetrical ligands, which often have challenging syntheses. Secondly, to investigate its catalytic activity in the SM coupling of aryl bromides in order to have a direct comparison to previously reported palladacycles. Finally, to determine the role of the donor atoms, and the donor atom substituents, in the bonding and stability of unsymmetrical SCN palladacycles, which is often not widely discussed [16].

## 2. Results and discussion

### 2.1. Synthesis of an SCN ligand and palladacycle

**Scheme 1.**
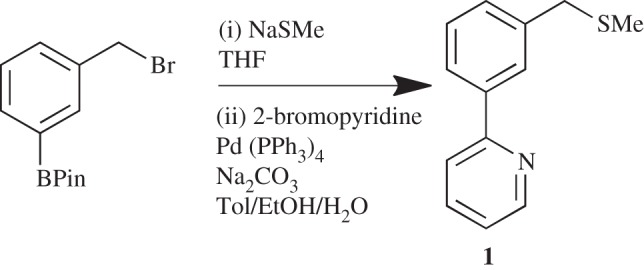
Previous synthesis of SCN ligand **1.**

**Scheme 2.**
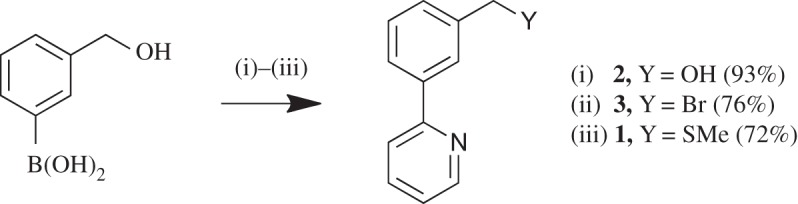
New synthesis of SCN ligand **1**. (i) 2-Bromopyridine, Pd(PPh_3_)_4_, base/solvent. (ii) HBr. (iii) NaSMe, EtOH.

The known ligand 2-{3-[(methylsulfanyl)methyl]phenyl}pyridine, **1**, has previously been prepared via the route shown in 78% yield (scheme 1) [32]. However, our desire was to introduce the sulfur nucleophiles at a later stage to allow future late-stage diversification (scheme 2), via the nucleophilic substitution of benzyl bromide **3**. Optimization of the first SM step was undertaken testing a variety of palladium catalysts and bases (table 1). From the conditions tested, it was found that Pd(PPh_3_)_4_ was the most effective catalyst compared with other ones tested: Pd(OAc)_2_, Pd(dppf)Cl_2_ and Buchwald's X Phos Pd G2 catalyst [33]. Varying the base using Pd(PPh_3_)_4_ as catalyst showed K_3_PO_4_ to be to the most effective and applying microwave (MW) heating was found to be advantageous (entry 8). The overall yield from the starting boronic acid to ligand **1** was 51%.

**Table 1.** Optimization of step (i) of scheme 2. Synthesis of SCN ligand **1** via SM coupling. 1 : 2 : 1 base : toluene : EtOH. (A) Thermal, 85°C, 48 h, (B) MW, 150°C, 10 min, (C) MW, 150°C, 20 min and (D) thermal, 85°C, 24 h.

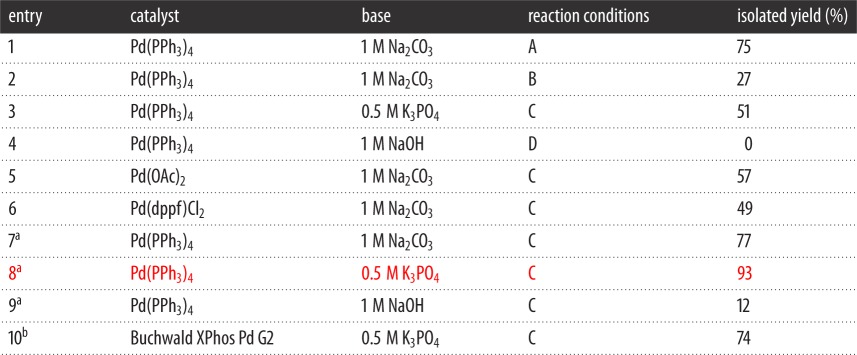

^a^10 : 7.5 : 5 base : toluene : EtOH.

^b^Methodology adapted from Buchwald *et al.* [33], 1.5 eq of boronic acid, 1 mol % catalyst, 1 : 2 base : THF.

**Scheme 3.**
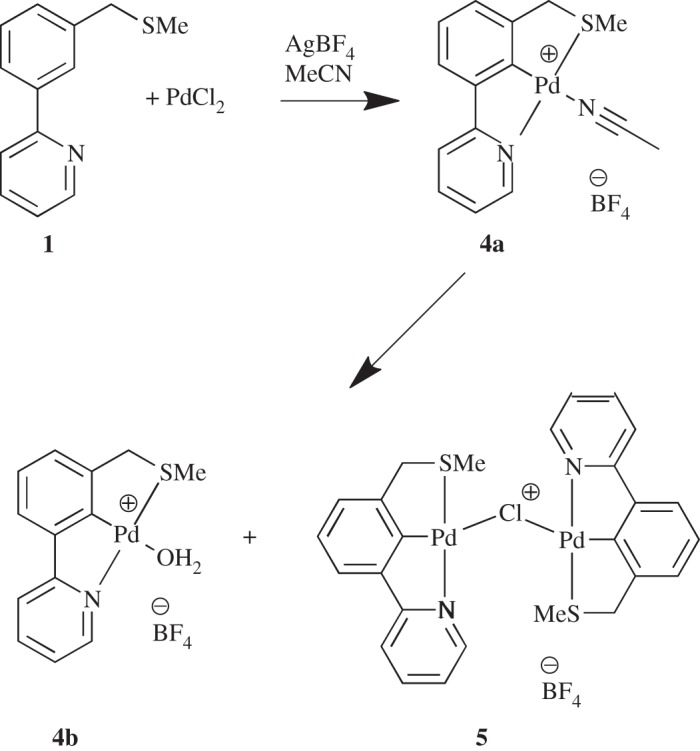
Synthesis of unexpected products **4b** and **5**.

Next, ligand **1** was selected for a C–H activation employing *in situ* generated Pd(MeCN)_4_(BF_4_)_2_ [34] (scheme 3). After work-up, two products were obtained, which was also suggested by high-resolution mass spectrometry (HRMS) data.

**Figure 2.**
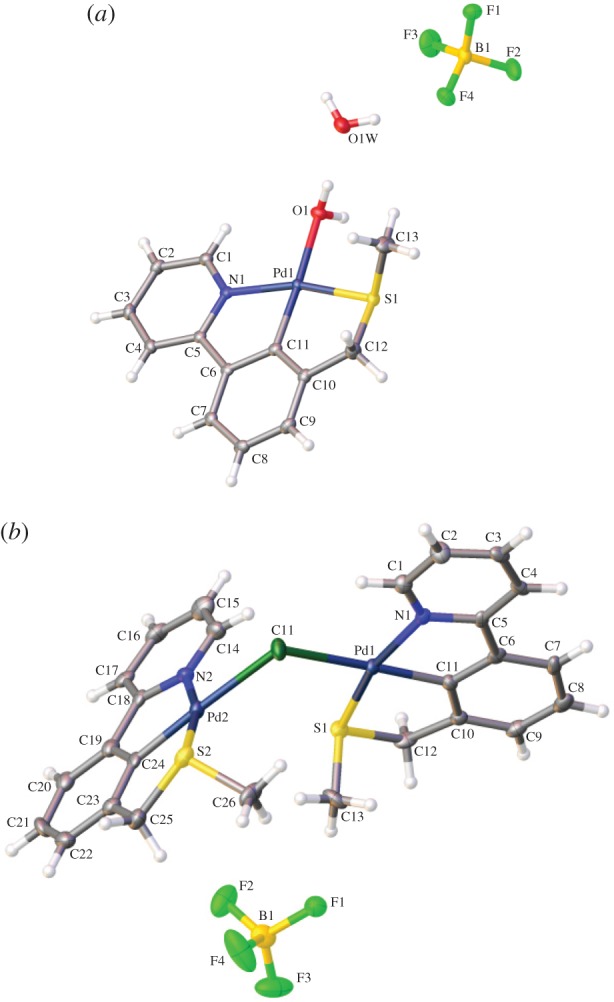
H_2_O bound palladacycle crystal structure **4b** (*a*) and chloride bridged palladacycle crystal structure **5** (*b*).

Thereafter, crystals were grown from the crude reaction mixture and X-ray crystal structures were determined for the water bound complex **4b** (figure 2), presumably formed from the expected acetonitrile complex **4a**, by water displacement (the crystallization was carried out in air) and the unusual dimeric structure **5** [35,36] which was probably formed from the formation of a monomeric chloro-palladacycle (from unreacted PdCl_2_) displacing solvent from either **4a** or **4b** (scheme 3).

**Figure 3.**
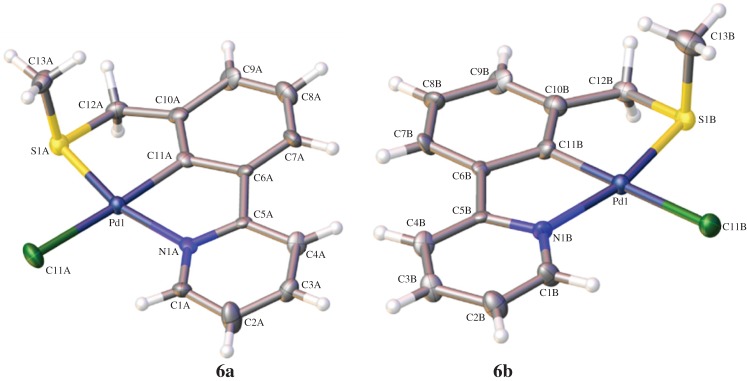
Monomeric palladacycle **6a** and **6b** crystal structures (formed from 50 : 50 ligand disorder in crystal lattice, separated for clarity).

**Scheme 4.**
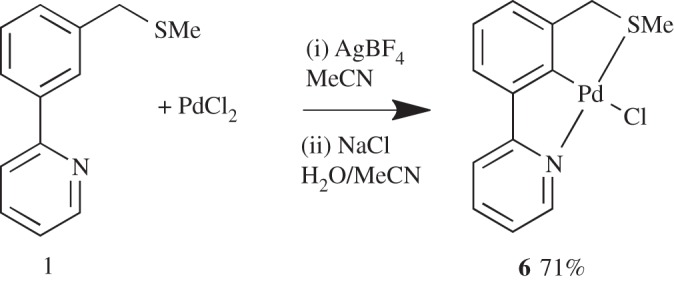
C–H activation followed by salt metathesis to **6**.

Repeating the C–H activation but subjecting the crude mixture to a simple salt metathesis (scheme 4) [17,37] gave the expected product **6** in 71% yield, and growth of crystals enabled its structural determination by X-ray diffraction to be carried out (figure 3). We now had a robust synthesis of **6** in order to synthesize quantities to be tested in catalytic applications.

### 2.2. X-ray crystal structure details

All three structures, **4b**, **5** and **6**, displayed a distorted square planar palladium(II) centre.

Structure **4b** crystallizes in the monoclinic P2_1_/c space group and comprises the H_2_O bound palladacycle, the tetrafluoroborate counterion and a water of crystallization. The palladacycle forms alternating stacks which propagate along the b-axis with the bound H_2_O forming a hydrogen bond to the water of crystallization (O^…^O = 2.67067(6) Å).

Structure **5** crystallizes in the monoclinic P2_1_ space group with the bridged palladacycle forming interleaved antiparallel stacks along the b-axis. This results in channels along the b-axis which are occupied by the tetrafluoroborate counterion.

Structure **6** crystallizes in the monoclinic P2_1_/c space group with the ligands disordered (50 : 50) across a mirror plane through the palladium centre and perpendicular to the rings of the SCN ligand. The structure comprises antiparallel tapes of **6** which propagate along the a-axis.

### 2.3. Catalytic investigations

The catalytic applications of palladacycle **6** have been investigated in the SM coupling. All reactions were performed in air using analytical grade solvents without further purification due to the air stability of the precatalyst **6**, which is advantageous for ease of use in the laboratory. Initial catalytic tests ascertained the minimum catalyst loading required for the coupling of 4-bromoanisole and phenylboronic acid (table 2). The conditions used were identical to those of Herrmann *et al.* [3]. The base used in each reaction was K_2_CO_3_, with the reaction undertaken in *o*-xylene at 130°C, with catalyst loadings from 0.001 to 0.5 mol %. Using gas chromatography (GC) conversions, a minimum catalyst loading of 0.01 mol % (entry 4) was deemed necessary, which was to be used in further catalytic runs. It was also found that, when performed under an argon atmosphere, lower catalyst loadings were possible, achieving conversions more than 50% with catalyst loadings as low as 0.0001 mol%; however, due to the very low catalyst loadings, concurrent results could not be obtained.

**Table 2.** SM coupling to determine minimum precatalyst loading.

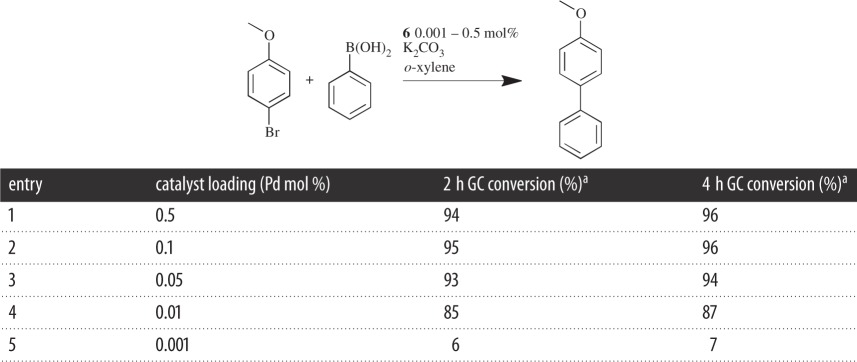

^a^Average of two runs based on 4-bromoanisole and product.

**Figure 4.**
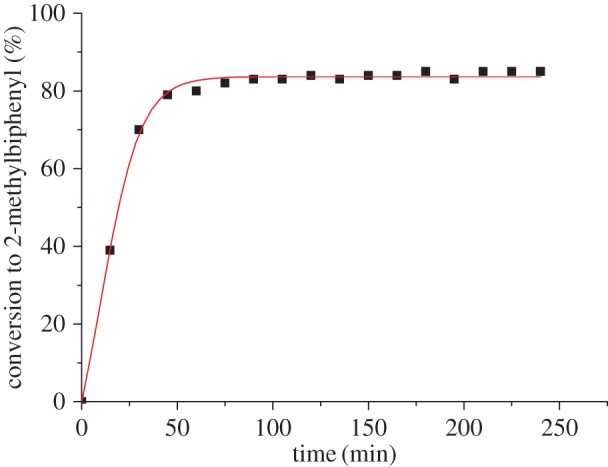
GC conversion of SM coupling of 2-bromotoluene and phenylboronic acid using **6** as a precatalyst, performed in duplicate.

To investigate optimal reaction conversions over time, 2-bromotoluene was coupled with phenylboronic acid using 0.01 mol% **6** using the conditions described previously, and the reaction monitored by GC every 15 min for 4 h (figure 4). The results show the reaction reaches maximum completion within 1 h. This suggests that the generation of the active palladium catalyst from the palladacycle precatalyst is rapid under these reaction conditions, with no improvement over time.

Following on from investigations into necessary precatalyst loading, the applicability of the precatalyst for challenging sterically hindered and electronically deactivated bromides was attempted using the previous conditions employed (table 3). All reactions were monitored via GC with conversions of starting material to product quoted. To validate GC conversions, in a related investigation, biaryl products have been isolated using related palladacycles in more than 70% yield, after purification (G. Roffe, J. Spencer 2014, unpublished data), and entry 1 confirmed via ^1^H NMR conversions.

**Table 3.** Testing of precatalyst^a^ using sterically hindered and electronically deactivated substrates.

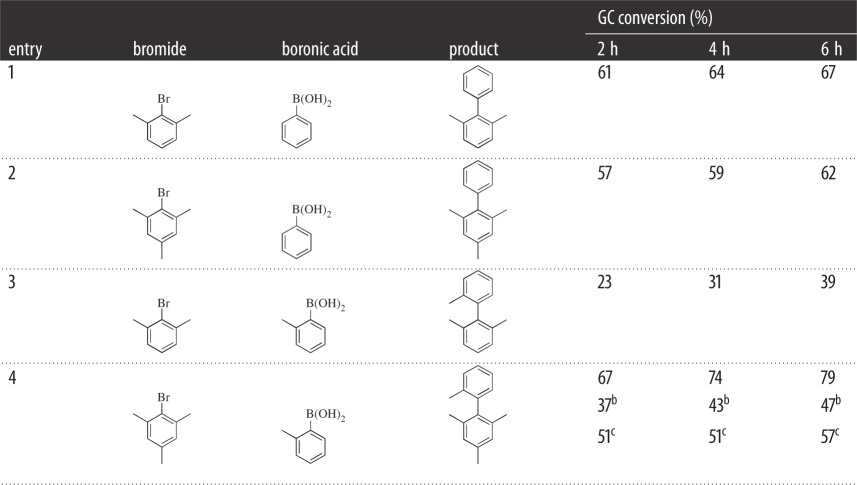

^a^0.01 mol % **6**, 2–6 h, *o*-xylene and K_2_CO_3_.

^b^Symmetrical SCS palladacycle replaced **6**.

^c^Herrmann–Beller palladacycle replaced **6**.

These catalytic tests, performed in duplicate, show moderate to good conversions of the starting *ortho* methylated aryl bromides to the corresponding biaryls. An interesting result is that the coupling of the 2-bromo-1,3-dimethylbenzene with 2-methylphenylboronic acid proceeds at much lower conversion than the corresponding more hindered bromomesitylene with 2-methylphenylboronic acid.

**Figure 5.**
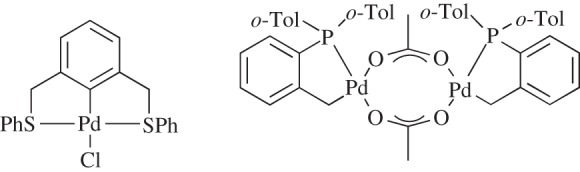
Symmetrical SCS palladacycle [38,39] and Herrmann–Beller catalyst [2].

However, overall, most of these substrates show similar conversions with **6** as the precatalyst. For entry 4, the results were compared to an SCS symmetrical example [38,39], and the Herrmann–Beller catalyst [2] (figure 5), revealing that our unsymmetrical example shows favourable results under these catalytic conditions. Having the ability to couple the much more challenging aryl chlorides would have been advantageous due to their greater availability and cheaper cost; however, in this case **6** was not successful, even with the addition of tetrabutylammonium bromide.

### 2.4. SCN model formation pathway

**Figure 6.**
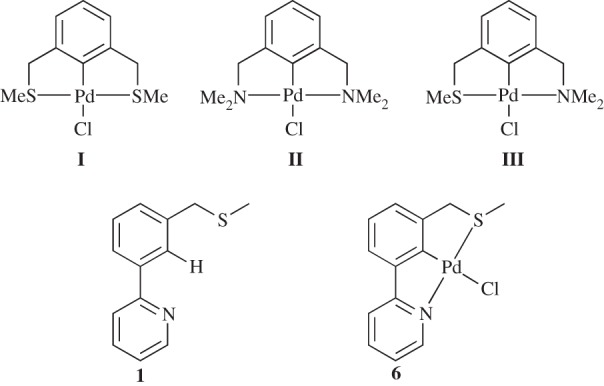
Symmetrical SCS (**I**) [40], symmetrical NCN (**II**) [41], and unsymmetrical SCN (**III**), unsymmetrical pyridine SCN ligand (**1**) and unsymmetrical pyridine SCN palladacycle (**6**).

Recently, we studied the formation reaction pathway of two symmetrical pincer palladacycles **I** [40] and **II** [41] (figure 6) from their respective ligands and palladium(II) chloride using DFT, and atoms in molecules (AIM) analysis was used to establish the nature of the bonding. Several computational model chemistries were investigated, and suitable candidates determined, one of which is used here [27]. In this work, we are interested in the effects when these palladacycles are desymmetrized, and we have therefore studied the formation reaction pathway towards **III**. We have also studied a potential formation pathway towards palladacycle **6** (figure 6).

Calculated (DFT) bond lengths and bond angles for the X-ray structures of **6** around the Pd centre are in excellent agreement when compared with the experimental values, confirming the accuracy of the DFT calculations. The errors in bond length are less than 0.023 Å and bond angles are within 1–2° with the exception of the Pd–S–CH_3_ bond angle which is overestimated by ≈ 6°.

**Scheme 5.**
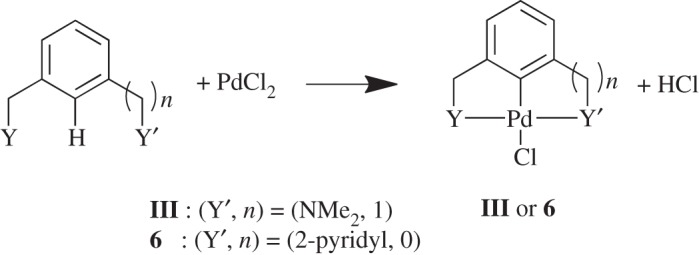
Model formation reaction, where Y = SMe, to unsymmetrical palladacycles **III** (*n* = 1, Y′ = NMe_2_) and **6** (*n* = 0, Y′ = pyridyl).

The formation reaction pathway investigated for **III** and **6** is shown in scheme 5. The pathway studied was based on that studied previously for the symmetrical examples [27], without the inclusion of an additional base in order to study the fundamental metal–donor atom interactions [42,43]. The Pd(II) source for C–H activation is modelled as monomeric PdCl_2_, as studied previously for Pd-based bond activations [44]. The use of monomeric PdCl_2_ is analogous to the use of monomeric Pd(OAc)_2_, or even the less computationally expensive Pd(η^2^-O_2_CH)_2_ [45], which are often used in calculations, instead of Pd_3_(OAc)_6_ [46].

The commonly discussed concerted metalation–deprotonation mechanism [28,30,31,47] involves assistance of deprotonation of the C–H bond by an acetate, carboxylate or pivalate base etc., resulting in very low energy barriers for C–H bond activation. However, in this work this is not relevant, as in the experimental conditions, these bases are not present.

Calculations were performed both without solvent to provide direct comparison with theoretical results on symmetric palladacycles [27], and with the inclusion of solvent effects as an energy correction using the polarization continuum model (PCM) [48,49] with acetonitrile as the solvent to model experimental conditions.

**Scheme 6.**
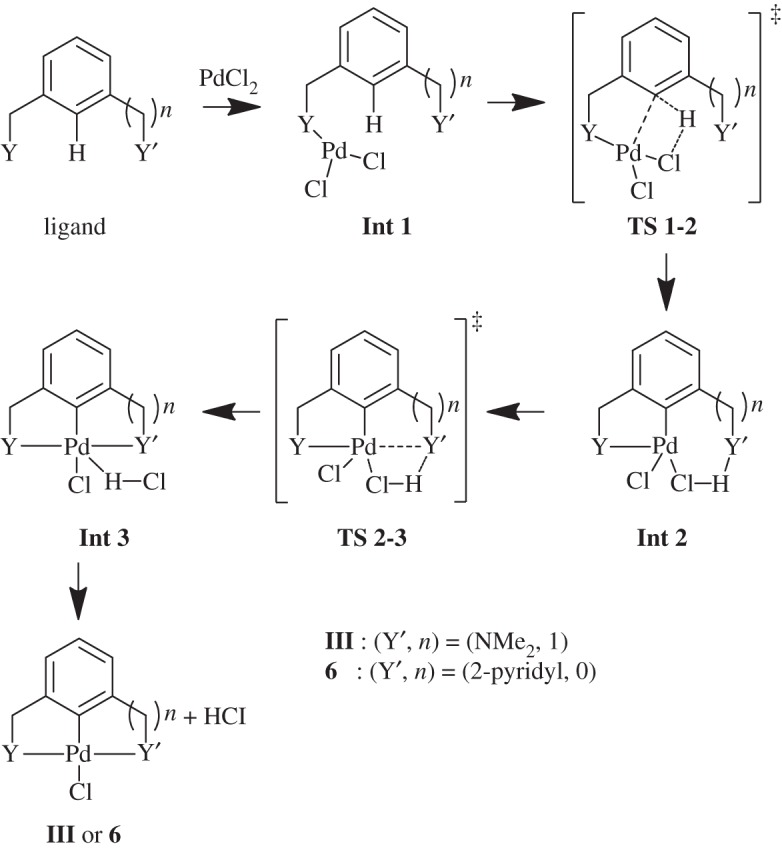
Model formation reaction, where Y = SMe, to unsymmetrical palladacycles **III** (Y^′^ = NMe_2_, *n* = 1) and **6** (Y^′^ = 2-pyridyl, *n* = 0). Pathway can occur with either Y coordinating to PdCl_2_ first (shown), or Y^′^ coordinating to PdCl_2_ first (not shown).

**Figure 7.**
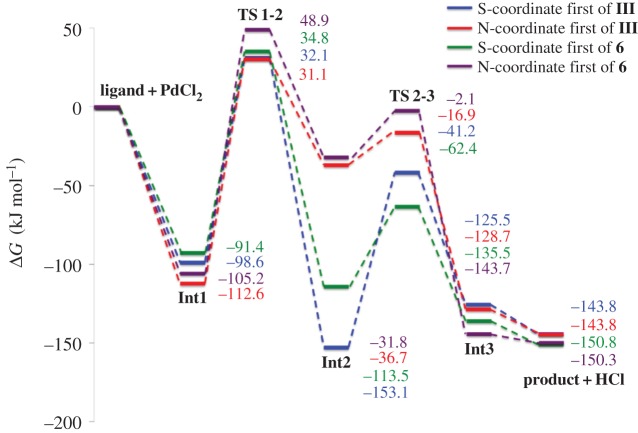
Gibbs free energies (solvent corrected using PCM, acetonitrile) for formation reaction pathways to **III** and **6** with Y or Y′ coordinating to PdCl_2_ first. **TS**, transition state; **Int**, intermediate.

The pathway towards **III** and **6** is shown in scheme 6 where each can occur via Y-coordination first, where Y = SMe, or via Y′-coordination first, where Y′ = NMe_2_ (**III**) or 2-pyridyl (**6**). The steps in the reaction include the initial ligand coordination to PdCl_2_ in **Int 1**, followed by C–H bond activation in **TS 1-2**, leading the new Pd–C bond in **Int 2** with a bridging HCl unit. The second ligand coordination step displaced the HCl, yielding the HCl adduct of the palladacycle in **Int 3**, which is then eliminated to form the final palladacycle **III** or **6**. The solvent-corrected Gibbs free energies, with both Y and Y′ coordinating first are shown in figure 7.

The energies for the formation reaction pathways (figure 7) show that both **III** and **6**, regardless of which ligand coordinates first are stable, with the formation of **6** slightly more energetically favourable than **III**. (The slightly different final energy values for **6** are due to the different conformers of ligand **1** needed for each pathway, and slight differences in the final structure of **6**.)

Insight into the role of the donor atoms can be gained by examining the stability of **Int 1**, where the ligand coordinates to PdCl_2_. In all cases, the ligand coordination is energetically more favourable, by at least 91 kJ mol^−1^, than the non-coordinated, free ligand. However, subtle differences emerge, depending on the ligand and which donor atom coordinates to Pd first. For both structures, **III** and **6**, N-coordination is more favourable than S-coordination (the difference between S- and N-coordinations is approx. 14 kJ mol^−1^ for both structures). In order to explain these differences, Bader's AIM theory [50] has been used to investigate the strength of the bonding between the palladium atom and either S or N in the formation of **6**.

**Figure 8.**
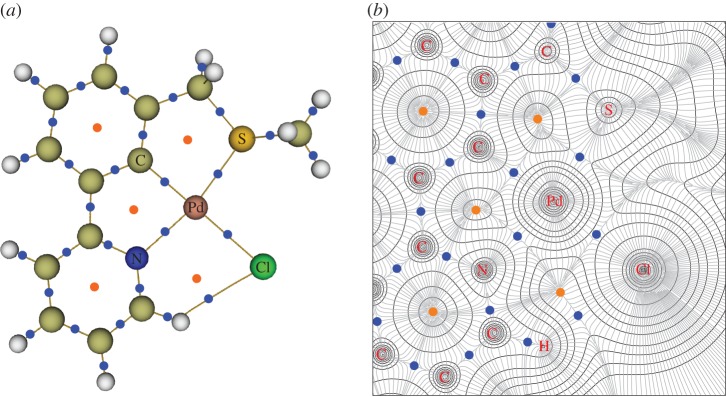
AIM analysis of **6** showing BCP (blue dots) and ring critical points (orange dots). (*a*) Molecular graph showing the bond paths between atoms in yellow, and (*b*) contour map of the electron density in the Cl–N–C plane of atoms coordinated to Pd showing the variation in the electron density.

In AIM analysis, chemical bonding can be characterized by first locating bond critical points (BCP): the point where the electron density becomes a minimum value along the bond path between interacting atoms, with key parameters at this point used to investigate the strength and nature of the bonding, tabulated fully in the electronic supplementary material. The topology of **6** is shown in figure 8, with BCP (blue dots) shown. In order to explain the more favourable N-coordination in **Int 1** of **III** and **6**, the electron density, *ρ*(**r**), at the BCP can be used. The magnitude of *ρ*(**r**) can be used to indicate the strength of a chemical bond [51]. Normally, *ρ*(**r**) is used to compare the strength of the same bond, for example, Te–N intramolecular interactions in different systems [52]. However, it has also been applied to study different bonds, such as M–H interactions for a variety of metals [53], or M–L bonds for a variety of metals and ligands [54], as in this work.

For **Int 1** in the formation pathway of **6**, the *ρ*(**r**) value is larger for N-coordination (0.096 au) than for S-coordination (0.087 au) indicating that the Pd–N bond is stronger than the Pd–S bond, attributed to more efficient orbital overlap in the Pd–N bond (see the electronic supplementary material) explaining the relative stabilities of the structures. The bond strengths of other key bonds are similar for the two structures.

Another key step in the formation reaction is the C–H bond activation, occurring at **TS 1-2**. This involves the cleaving of the C–H bond shown by decreasing *ρ*(**r**) values (from approx. 0.3 au in **Int 1** to approx. 0.1 au in **TS 1-2**), and the formation of a new Cl–H bond, resulting in a newly formed BCP. The activation barriers for this step vary for **III** and **6** depending on whether S or N coordinates first. The C–H bond activation barriers are smaller for S-coordination by 13 and 28 kJ mol^−1^ for **III** and **6,** respectively (figure 7). This difference has been investigated in terms of the Bader charge from the AIM analysis. The palladium charge in PdCl_2_ is 0.683 au, and in **TS 1-2** for pathway to **6** is 0.587 au with S coordinated to palladium, and 0.706 au with N coordinated to palladium. Clearly, the S donor is more electron donating due to the smaller Pd charge than for the N donor (this is also true for **Int 1**). The electrophilic Pd(II) centre [55] is therefore more stabilized by the S-coordination in the C–H bond activation step, resulting in the lower barrier compared with the N-coordinated examples.

The largest difference in relative stabilities occurs at **Int 2** (figure 7), which corresponds to the Pd coordinated to one of the donor atoms of the pincer ligand after inserting into the C–H bond. These energetic differences can be attributed to an interaction between H and the other donor atom. When the Pd is coordinated by N first, the H interacts with SMe. Therefore, the energies of **Int 2** in the formation of **III** and **6** are very similar. However, in the formation of **III** when Pd coordinates to the S first, a very strong H–NMe_2_ interaction occurs, forming a very stable structure. In the absence of solvent corrections **III** is approximately 30 kJ mol^−1^ more stable that **Int 2**, but with the addition of the solvent corrections (shown in figure 7) **Int 2** is slightly more stable than the product **III** (by 9.3 kJ mol^−1^). This interaction is slightly stronger than the H–pyridyl interaction in the formation of **6**. This is supported by *ρ*(**r**) values at the BCP between H and the respective donor atom (see the electronic supplementary material).

This analysis demonstrates the interplay between different bond strengths, with Pd–S interactions being weaker than Pd–N interactions for **Int 1** and **TS 1-2**, and the different bonding nature of S compared with N with different electron donation abilities having significant effects on the thermodynamics and kinetics of this cyclometallation mechanism. These effects could also have significance in the catalyst activation pathway in the SM reaction where the palladacycle is reduced to the catalytically active Pd(0) species [20,37,56–58].

## 3. Conclusion

Ligand **1** was readily synthesized via a key catalytic C–C bond forming reaction as one of the synthetic steps. C–H activation of **1** with *in situ* generated Pd(MeCN)_4_(BF_4_)_2_ led to a mixture of pincer palladacycles, which were converted to the desired unsymmetrical SCN pincer **6**. The overall yield from starting materials to the final product was 36%. Each SCN pincer palladacycle **6, 4b** and **5** was characterized in the solid state by X-ray crystallography. The monomeric chloride example **6** was tested as a precatalyst in a number of SM reactions of sterically challenging and electronically deactivated aryl bromides, showing favourable conversions in comparison to similar catalysts.

The bonding and stability of two unsymmetrical SCN palladacycles YCY′, **III** (Y = SMe, Y′ = NMe_2_, *n* = 1) and **6** (Y = SMe, Y′ = 2-pyridyl, *n* = 0), have been investigated using DFT. It is shown, based on a simple formation reaction with solvent effect of acetonitrile included through use of the PCM, that both palladacycles are thermodynamically stable, with **6** more stable than **III**, and the formation is spontaneous. For both structures two pathways were found, dependent on which donor atom of the ligand coordinates to palladium first. It was found that for the rate determining C–H bond activation step, in all cases, the barrier is influenced by the electron donating ability of the ligand atoms, with barriers lower by 13–28 kJ mol^−1^ when sulfur is coordinated to palladium rather than nitrogen. Unsymmetrical palladacycles clearly provide the opportunity for using the electron donating ability of the ligand atoms to alter key reaction steps, which could have implications in the catalyst activation pathway.

Future work will concentrate on exploiting our new synthetic method enabling late-stage derivatization of the biaryls **2** and **3**, enabling the potential synthesis of libraries of SCN, unsymmetrical NCN and PCN pincer palladacycle analogues. The catalyst activation pathways are also being investigated via DFT. Moreover, the introduction of unsymmetrical ligands should be useful in other areas of pincer chemistry [8]. Structures **4b**, **5** and **6** were given CCDC numbers 1033101, 1033102 and 1033103, respectively.

## 4. Experimental details

### 4.1. General details

Solvents and chemicals were purchased from commercial suppliers and used without further purification, with most reactions taking place open to atmosphere and moisture. C–H activation reactions were undertaken using Schlenk techniques, under nitrogen, in dry acetonitrile. MW reactions were performed using CEM explorer equipment.

### 4.2. Instrumentation

^1^H and ^13^C spectra were recorded on either a Varian 500 MHz or an ECP 400 MHz spectrometer. HRMS was conducted with an ESI mass spectrometer using a Bruker Daltonics Apex III, with ESI source Apollo ESI, using methanol as the spray solvent by Dr Alaa K. Abdul-Sada of the University of Sussex Mass Spectrometry Centre. GC measurements were obtained using a Perkin Elmer Autosystem XL gas chromatograph, using a flame ionization detector and a Supelco MDN-5S 30 m × 0.25 mm × 0.25 µm column, with a He mobile phase. Elemental analyses were run by the London Metropolitan University Elemental Analysis Service. Crystal structures were obtained by the UK National Crystallography Service at the University of Southampton as described previously [59].

### 4.3. [3-(Pyridin-2-yl)phenyl]methanol (**2**)

(3-(Hydroxymethyl)phenyl)boronic acid (4.04 mmol, 614 mg), 2-bromopyridine (4.04 mmol, 0.393 ml), Pd(PPh_3_)_4_ (0.16 mmol, 182 mg), 0.5 M K_3_PO_4_ (10 ml), toluene (7.5 ml) and EtOH (5 ml) were added to a sealed 35 ml MW vial and stirred under MW irradiation (maximum power 300 W, using dynamic heating) at 150°C for 20 min. The mixture was left to cool to room temperature, and the solvent was removed *in vacuo*. The mixture was diluted with H_2_O (25 ml) and EtOAc (25 ml). The crude product was extracted with EtOAc (2 × 25 ml), washed with H_2_O (2 × 25 ml) and brine (25 ml). The combined organic layers were dried over anhydrous MgSO_4_, filtered and concentrated *in vacuo*. The crude material was purified using flash column chromatography (7 : 3 DCM:EtOAc) yielding 694 mg of the expected product **2** as a yellow solid in 93% yield. ^1^H NMR (500 MHz, chloroform-*d*) δ 8.70 (d, *J* = 4.9 Hz, 1H), 8.02 (s, 1H), 7.90 (d, *J* = 7.6 Hz, 1H), 7.78–7.74 (m, 2H), 7.48 (dd, *J* = 7.6, 7.6 Hz, 1H), 7.44 (d, *J* = 7.6 Hz, 1H), 7.24 (ddd, *J* = 7.3, 4.9, 2.7 Hz, 1H), 4.80 (d, *J* = 6.0 Hz, 2H), 1.79 (t, *J* = 6.0 Hz, 1H). ^13^C NMR (126 MHz, chloroform-*d*) δ 157.3, 149.7, 141.5, 139.7, 136.8, 129.0, 127.5, 126.2, 125.5, 122.2, 120.6, 65.4. HRMS. Calcd for [C_12_H_11_NO + Na]^+^ 208.0733. Found 208.0731.

### 4.4. 2-[3-(Bromomethyl)phenyl]pyridine (**3**)

(3-(Pyridin-2-yl)phenyl)methanol (**2**) (3.03 mmol, 561 mg) and greater than or equal to 48% HBr in H_2_O (5 ml) were added to a 10 ml round bottomed flask and stirred at 125°C for 8 h, then left to stir overnight at room temperature. The reaction mixture pH was carefully adjusted to approximately 7.5 by careful addition of a saturated NaHCO_3_ solution. The crude product was extracted with EtOAc (3 × 50 ml), washed with H_2_O (3 × 50 ml) and brine (50 ml). The organic layers were dried over anhydrous MgSO_4_, filtered and solvent removed *in vacuo*. The crude product was purified using flash column chromatography (9 : 1 DCM : EtOAc) yielding 568 mg of the expected product **3** as a yellow oil in 76% yield. ^1^H NMR (500 MHz, chloroform-*d*) δ 8.70 (d, *J* = 4.9 Hz, 1H), 8.06 (s, 1H), 7.93–7.89 (m, 1H), 7.79–7.73 (m, 2H), 7.48–7.44 (m, 2H), 7.25 (ddd, *J* *=* 6.7, 4.9, 1.7 Hz, 1H), 4.58 (s, 2H). ^13^C NMR (126 MHz, chloroform-*d*) δ 156.7, 149.7, 134.0, 138.4, 136.8, 129.6, 129.2, 127.6, 126.9, 122.4, 120.6, 33.4. HRMS. Calcd for [C_12_H_10_BrN + H]^+^ 248.0069. Found 248.0071.

### 4.5. 2-{3-[(Methylsulfanyl)methyl]phenyl}pyridine (**1**)

2-(3-(Bromomethyl)phenyl)pyridine (**3**) (1.33 mmol, 331 mg), sodium thiomethoxide (1.62 mmol, 114 mg) and EtOH (4 ml) were added to a sealed MW vial and stirred under MW irradiation (maximum power 300 W, dynamic heating) at 150°C for 20 min. After cooling, the solvent was removed *in vacuo*, and the crude mixture was diluted with H_2_O (25 ml) and EtOAc (25 ml). The crude product was extracted with EtOAc (2 × 25 ml), washed with H_2_O (2 × 25 ml) and brine (25 ml). The organic layers were dried over anhydrous Na_2_SO_4_, filtered and concentrated *in vacuo*. The crude product was purified by flash column chromatography (9 : 1 DCM:EtOAc) yielding 207 mg of the expected product **1** as a yellow oil in 72% yield. ^1^H and ^13^C NMR spectra are in agreement with prior literature values [32].

### 4.6. Complexes **4b** and **5**

Palladium chloride (0.49 mmol, 87 mg) and MeCN (25 ml) were placed in a round bottomed flask and stirred under reflux (85°C) under a nitrogen atmosphere until all PdCl_2_ was dissolved. Silver tetrafluoroborate (0.98 mmol, 191 mg) was added and left to stir under reflux in a nitrogen atmosphere for 2 h. The mixture was then cooled and filtered over Celite. A solution of **1** (0.47 mmol, 100 mg) in MeCN (10 ml) was added to the filtrate and the solution stirred under reflux and nitrogen atmosphere for 6 h. The mixture was cooled to room temperature and filtered. The filtrate was concentrated to give 305 mg of a yellow solid which was purified by chromatography (DCM : MeOH 95 : 5) to give 133 mg of a light yellow solid. ^1^H NMR (400 MHz, DMSO-*d*_6_) δ 8.42 (d, *J* = 5.6 Hz, 1H), 8.19–8.11 (m, 2H), 7.64 (d, *J* = 7.6 Hz, 1H), 7.53 (ddd, *J* = 7.3, 5.6, 1.8 Hz, 1H), 7.18 (dd, 1H, *J* = 7.6 Hz), 7.11 (d, *J* = 7.6 Hz, 1H), 4.46 (m, 2H), 2.82 (s, 3H). ^13^C NMR (100 MHz, DMSO-*d*_6_) δ 164.4, 149.7, 148.6, 144.2, 141.4, 126.6, 125.8, 124.5, 123.5, 120.7, 46.4, 23.0 (1 carbon missing). ^19^F NMR (376 MHz, DMSO-*d*_6_) δ –148.33, –148.39. HRMS shows the presence of a mixture of structures.

### 4.7. 2-{3-[(Methylsulfanyl)methyl]phenyl}pyridine chloro-palladacycle (**6**)

The C–H activation technique was repeated as per synthesis of **4b** and **5**, and the crude reaction mixture was dissolved in MeCN (5 ml), and sodium chloride (19.9 mmol, 1.17 g) dissolved in H_2_O (5 ml) was added, and the mixture was stirred at room temperature for 3 h. The solvent was removed *in vacuo,* the crude mixture was then dissolved in DCM (35 ml) and H_2_O (35 ml) was added. The crude product was extracted with DCM (2 × 35 ml), washed with H_2_O (2× 35 ml) and brine (35 ml), dried over anhydrous MgSO_4_, filtered over Celite and the solvent was removed *in vacuo*. The crude product was purified by flash column chromatography (100% DCM → 98 : 2 DCM:MeOH) yielding 244 mg of the expected product **6** as a yellow solid in 71% yield. Crystals were grown by slow evaporation of DCM from a solution of the sample. ^1^H NMR (500 MHz, chloroform-*d*) δ 9.06 (s, 1H), 7.83 (dd, *J* = 7.9, 7.9 Hz, 1H), 7.61 (d, *J* = 7.9 Hz, 1H), 7.30 (d, *J* = 7.6 Hz, 1H), 7.22 (m, 1H), 7.06 (dd, *J* = 7.6, 7.6 Hz, 1H), 7.01 (d, *J* = 7.6 Hz, 1H), 4.30 (m, 2H), 2.84 (s, 3H).^13^C NMR (100 MHz, chloroform-*d*) δ 165.7, 165.2, 150.4, 147.8, 144.3, 138.9, 125.1, 124.7, 122.8, 122.2, 118.7, 49.4, 23.7. HRMS. Calcd for [C_13_H_12_NPdS]^+^ 319.9720. Found 319.9710. Anal. Calcd for C_13_H_12_NPdSCl: C, 43.84; H, 3.40; N, 3.93. Found: C, 43.71; H, 3.48; N, 3.93.

### 4.8 General method for Suzuki–Miyaura catalytic tests

The aryl bromide (1 mmol), boronic acid (1.5 mmol), K_2_CO_3_ (2 mmol), **6** (25 µl standard solution in chloroform, concentration depending on catalyst loading) and *o*-xylene (3 ml) were added to a reaction vessel and heated at 130°C. 0.1 ml aliquots were taken at various time intervals, washed with H_2_O and extracted with Et_2_O. A sample of the Et_2_O solution was then used for GC analysis.

## 5. Computational details

The structures and energies of all structures were calculated using DFT as implemented in GAUSSIAN 09 [60]. Geometry optimization and frequency analysis was performed using the ωB97XD [61] exchange-correlation functional. In each case, structural minima and transition states were verified by the absence or the presence of a single imaginary vibrational mode, respectively. Transition states were confirmed by eigenvector following calculations. For geometry optimizations, a 6-31++G(d,p) basis set was used for all atoms except Pd, for which the standard SDD pseudopotential was used [62]; this will be referred to as 6-31++G(d,p) [SDD]. The ωB97XD functional was chosen for this study after testing a range of functionals on their ability to reproduce the geometries of known symmetric pincer palladacycles [27]. Single point energy calculations were performed on both the X-ray structures and the ωB97XD optimized structures at the ωB97XD/6-311++G(2df,2p)[SDD] level of theory. Solvation effects were accounted for by performing single point energy calculations on the optimized geometries using the self-consistent reaction field PCM [48,49] with universal force field atomic radii at the ωB97XD/6-311++G(2df,2p)[SDD] level of theory. The solvent acetonitrile (*ϵ* = 35.688) was used in this study.

The ωB97XD functional [61] was chosen for the energy calculations due to the benefits of variable HF exchange with distance and the empirical dispersion correction. Furthermore, the reaction energy benchmark study by Zhao & Truhlar [63] showed the ωB97XD functional to have one of the smallest average mean unsigned errors of the 30 functionals tested.

The topological analysis of the electron density was performed using the AIM [64] method as implemented in the Multiwfn program [65]. In order to obtain reliable AIM parameters, the effective core potential basis set for Pd was replaced with the all electron basis set, DGDZVP [66], to generate the wave function at the ωB97XD level of theory, i.e. using ωB97XD/6-311++G(2df,2p)[DGDZVP].

## Supplementary Material

Supporting Information final draft

## Glossary of terms

DCM: dichloromethane
EtOAc: ethyl acetate
MeCN: acetonitrile
MeOH: methanol.
