# Supplementary material for: A synthetic, catalytic and theoretical investigation of an unsymmetrical SCN pincer palladacycle
Source: R Soc Open Sci. 2016 Apr 6;3(4):150656. doi: 10.1098/rsos.150656 (PMC4852630; doi:10.1098/rsos.150656)
Supplement: Supporting Information final draft [file rsos150656supp1.docx]

**Supporting information**

A Synthetic, Catalytic and Theoretical Investigation of an Unsymmetrical SCN Pincer Palladacycle

Gavin W. Roffe^a^, Sarote Boonseng^a^, Christine B. Baltus^b^, Simon J. Coles^c^, Iain J. Day^a^, Rhiannon N. Jones^a^, Neil J. Press^d^, Mario Ruiz^b^, Graham J. Tizzard^c^, Hazel Cox^a,^* and John Spencer^a,b,^*

^a.^ Department of Chemistry, School of Life Sciences, University of Sussex, Falmer, Brighton, East Sussex, BN1 9QJ, UK.
^b.^ School of Science, University of Greenwich at Medway, University of Greenwich, Chatham, ME4 4TB, UK.

^c.^ UK National Crystallography Service, School of Chemistry, University of Southampton, Highfield, Southampton, SO17 1BJ, UK.
^d.^ Novartis Pharmaceuticals UK Ltd, Horsham, Sussex, RH12 5AB, UK.

TABLE OF CONTENTS

|  | Title | Page |
| --- | --- | --- |
| S1 | Supporting Experimental Data. | 3 |
| S2 | Energies for formation reaction pathways to **III** and **6**. | 27 |
| S3 | Bader charge and AIM analysis data for formation pathway to **III** for S-coordination first for key atoms and bonds. | 28 |
| S4 | Bader charge and AIM analysis data for formation pathway to **III** for N-coordination first for key atoms and bonds. | 31 |
| S5 | Bader charge and AIM analysis data for formation pathway to **6** for S-coordination first for key atoms and bonds. | 35 |
| S6 | Bader charge and AIM analysis data for formation pathway to **6** for N-coordination first for key atoms and bonds. | 38 |
| S7 | Bader charge and AIM analysis data for PdCl_2_. | 41 |
| S8 | Molecular orbitals involving significant Pd-donor atom interactions in **Int 1** for **6**. | 42 |
| S9 | Cartesian coordinates, energies, zero-point energy correction, thermal correction to Gibbs free energy, single point and solvent corrected energies for pathway to **III** with S-coordination first. | 46 |
| S10 | Cartesian coordinates, energies, zero-point energy correction, thermal correction to Gibbs free energy, single point and solvent corrected energies for pathway to **III** with N-coordination first. | 53 |
| S11 | Cartesian coordinates, energies, zero-point energy correction, thermal correction to Gibbs free energy, single point and solvent corrected energies for pathway to **6** with S-coordination first. | 61 |
| S12 | Cartesian coordinates, energies, zero-point energy correction, thermal correction to Gibbs free energy, single point and solvent corrected energies for pathway to **6** with N-coordination first. | 69 |
| S13 | Cartesian coordinates, energies, zero-point energy correction, thermal correction to Gibbs free energy, single point and solvent corrected energies for HCl. | 76 |
| S14 | Cartesian coordinates, energies, zero-point energy correction, thermal correction to Gibbs free energy, single point and solvent corrected energies for PdCl­_2_. | 76 |

**S1**: Supporting Experimental Data.

|  | Title | Page |
| --- | --- | --- |
| Table 1 | Crystal data and structure refinement details for structure **4b** | 4 |
| Table 2 | Crystal data and structure refinement details for structure **5** | 5 |
| Table 3 | Crystal data and structure refinement details for structure **6** | 7 |
| Figure 1 | ^1^H NMR spectrum of [3-(pyridin-2-yl)phenyl]methanol (**2**) | 9 |
| Figure 2 | ^13^C spectrum of [3-(pyridin-2-yl)phenyl]methanol (**2**) | 10 |
| Figure 3 | HRMS spectrum of [3-(pyridin-2-yl)phenyl]methanol (**2**) | 11 |
| Figure 4 | ^1^H spectrum of 2-[3-(bromomethyl)phenyl]pyridine (**3**) | 12 |
| Figure 5 | ^13^C spectrum of 2-[3-(bromomethyl)phenyl]pyridine (**3**) | 13 |
| Figure 6 | HRMS of 2-[3-(bromomethyl)phenyl]pyridine (**3**) | 14 |
| Figure 7 | ^1^H of 2-{3-[(methylsulfanyl)methyl]phenyl}pyridine (**1**) | 15 |
| Figure 8 | ^13^C of 2-{3-[(methylsulfanyl)methyl]phenyl}pyridine (**1**) | 16 |
| Figure 9 | ^1^H of mixture of **4b** and **5** | 17 |
| Figure 10 | ^13^C of mixture of **4b** and **5** | 18 |
| Figure 11 | ^19^F of mixture of **4b** and **5** | 19 |
| Figure 12 | HRMS of mixture of **4b** and **5** | 20 |
| Figure 13 | ^1^H of 2-{3-[(methylsulfanyl)methyl]phenyl}pyridine chloro palladacycle (**6**) | 21 |
| Figure 14 | ^13^C 2-{3-[(methylsulfanyl)methyl]phenyl}pyridine chloro palladacycle (**6**) | 22 |
| Figure 15 | Elemental analysis of 2-{3-[(methylsulfanyl)methyl]phenyl}pyridine chloro palladacycle (**6**) | 23 |
| Figure 16 | HRMS of 2-{3-[(methylsulfanyl)methyl]phenyl}pyridine chloro palladacycle (**6**) | 24 |
| Figure 17 | Crude ^1^H NMR conversion of 2-bromo-1,3-dimethylbenzene to 1,3-dimethyl-2-phenylbenzene. | 25 |

**Table 1.** Crystal data and structure refinement details for structure **4b**.

|  |
| --- |

Identification code **2011ncs0576a**

Empirical formula C_13_H_16_BF_4_NO_2_PdS

Formula weight 443.54

Temperature 100(2) K

Wavelength 0.71075 Å

Crystal system Monoclinic

Space group *P*21/*c*

Unit cell dimensions *a* = **12.2089(4) Å** *α* = **90°**

*b* = **8.0243(2) Å** *β* = **90.738(6)°**

*c* = **15.9603(11) Å** *γ* = **90°**

Volume 1563.47(13) Å^3^

*Z* 4

Density (calculated) 1.884 Mg / m^3^

Absorption coefficient 1.368 mm**^−1^**

*F(000)* 880

Crystal chunk; colourless

Crystal size 0.08 × 0.07 × 0.06 mm^3^

*θ* range for data collection 3.03 − 27.48°

Index ranges −15 ≤ *h* ≤ 14, −10 ≤ *k* ≤ 9, −10 ≤ *l* ≤ 20

Reflections collected 7151

Independent reflections 3564 [*R_int_* = 0.0166]

Completeness to *θ* = 27.48° 99.3 %

Absorption correction Semi−empirical from equivalents

Max. and min. transmission 0.9224 and 0.8984

Refinement method Full-matrix least-squares on *F*^2^

Data / restraints / parameters 3564 / 6 / 221

Goodness-of-fit on *F*^2^ 0.917

Final *R* indices [*F*^2^ > 2*σ*(*F*^2^)] *R1* = 0.0184, *wR2* = 0.0516

*R* indices (all data) *R1* = 0.0205, *wR2* = 0.0527

Largest diff. peak and hole 0.397 and −0.341 e Å**^−3^**

|  |
| --- |

**Diffractometer**: *Rigaku AFC12 Kappa 3-circle with Saturn724+* area detector (*ω* scans to fill *asymmetric unit* sphere). **Cell determination:** *CrystalClear-SM Expert 2.0 r7* (Rigaku, 2011). **Data collection:** *CrystalClear-SM Expert 2.0 r7* (Rigaku, 2011). **Data reduction and cell** **refinement**: *CrystalClear-SM Expert 2.0 r7* (Rigaku, 2011). **Absorption correction**: *CrystalClear-SM Expert 2.0 r7* (Rigaku, 2011). S**tructure solution**: *SUPERFLIP* (Palatinus, L. & Chapuis, G. (2007). J. Appl. Cryst. 40, 786-790.) S**tructure refinement**: *SHELXL97* (G Sheldrick, G.M. (2008). Acta Cryst. A64, 112-122.). **Graphics:** *OLEX2* (Dolomanov, O. V., Bourhis, L. J., Gildea, R. J., Howard, J. A. K. & Puschmann, H. (2009). J. Appl. Cryst. 42, 339-341.)

**Table 2.** Crystal data and structure refinement details for structure **5**.

|  |
| --- |

Identification code **2011src0204c**

Empirical formula C_26_H_24_BClF_4_N_2_Pd_2_S_2_

Formula weight 763.65

Temperature 120(2) K

Wavelength 0.71073 Å

Crystal system Monoclinic

Space group *P*21

Unit cell dimensions *a* = **10.1188(4) Å** *α* = **90°**

*b* = **10.8578(2) Å** *β* = **101.8840(10)°**

*c* = **12.1214(4) Å** *γ* = **90°**

Volume 1303.21(7) Å^3^

*Z* 2

Density (calculated) 1.946 Mg / m^3^

Absorption coefficient 1.692 mm**^−1^**

*F(000)* 752

Crystal block; colourless

Crystal size 0.16 × 0.14 × 0.06 mm^3^

*θ* range for data collection 2.94 − 27.48°

Index ranges −13 ≤ *h* ≤ 13, −14 ≤ *k* ≤ 14, −15 ≤ *l* ≤ 13

Reflections collected 16248

Independent reflections 5855 [*R_int_* = 0.0614]

Completeness to *θ* = 27.48° 99.8 %

Absorption correction Semi−empirical from equivalents

Max. and min. transmission 0.9053 and 0.7735

Refinement method Full-matrix least-squares on *F*^2^

Data / restraints / parameters 5855 / 1 / 343

Goodness-of-fit on *F*^2^ 1.048

Final *R* indices [*F*^2^ > 2*σ*(*F*^2^)] *R1* = 0.0436, *wR2* = 0.0876

*R* indices (all data) *R1* = 0.0532, *wR2* = 0.0928

Absolute structure parameter 0.00(3)

Largest diff. peak and hole 1.402 and −0.831 e Å**^−3^**

**Diffractometer**: *Nonius KappaCCD* area detector (*φ* scans and *ω* scans to fill *asymmetric unit* sphere). **Cell determination:** DirAx (Duisenberg, A.J.M.(1992). J. Appl. Cryst. 25, 92-96.) **Data collection:** Collect (Collect: Data collection software, R. Hooft, Nonius B.V., 1998). **Data reduction and cell** **refinement**: *Denzo* (Z. Otwinowski & W. Minor, *Methods in Enzymology* (1997) Vol. **276**: *Macromolecular Crystallography*, part A, pp. 307−326; C. W. Carter, Jr. & R. M. Sweet, Eds., Academic Press). **Absorption correction**: *SADABS* (Sheldrick, G. M. (2007). SADABS. Version 2007/2. Bruker AXS Inc., Madison, Wisconsin, USA.). S**tructure solution**: SUPERFLIP (Palatinus, L. & Chapuis, G. (2007). J. Appl. Cryst. 40, 786-790.) S**tructure refinement**: *SHELXL97* (G Sheldrick, G.M. (2008). Acta Cryst. A64, 112-122.). **Graphics:** *OLEX2* (Dolomanov, O. V., Bourhis, L. J., Gildea, R. J., Howard, J. A. K. & Puschmann, H. (2009). J. Appl. Cryst. 42, 339-341.)

**Table 3.** Crystal data and structure refinement details for structure **6**.

|  |
| --- |

Identification code **2013ncs0354aa**

Empirical formula C_13_H_12_ClNPdS

Formula weight 356.15

Temperature 100(2) K

Wavelength 0.71075 Å

Crystal system Monoclinic

Space group *P*21/*c*

Unit cell dimensions *a* = **9.1457(5) Å** *α* = **90°**

*b* = **12.4958(9) Å** *β* = 10**7.814(2)°**

*c* = **11.6610(8) Å** *γ* = **90°**

Volume 1268.76(14) Å^3^

*Z* 4

Density (calculated) 1.864 Mg / m^3^

Absorption coefficient 1.812 mm**^−1^**

*F(000)* 704

Crystal Block; Colorless

Crystal size 0.120 × 0.060 × 0.040 mm^3^

*θ* range for data collection 2.978 − 27.486°

Index ranges −11 ≤ *h* ≤ 11, −15 ≤ *k* ≤ 16, −9 ≤ *l* ≤ 15

Reflections collected 8682

Independent reflections 2889 [*R_int_* = 0.0293]

Completeness to *θ* = 25.242° 99.6 %

Absorption correction Semi−empirical from equivalents

Max. and min. transmission 1.000 and 0.690

Refinement method Full-matrix least-squares on *F*^2^

Data / restraints / parameters 2889 / 404 / 282

Goodness-of-fit on *F*^2^ 1.421

Final *R* indices [*F*^2^ > 2*σ*(*F*^2^)] *R1* = 0.0906, *wR2* = 0.2382

*R* indices (all data) *R1* = 0.0914, *wR2* = 0.2385

Extinction coefficient n/a

Largest diff. peak and hole 2.340 and −2.408 e Å**^−3^**

|  |
| --- |

**Diffractometer:** *Rigaku AFC12* goniometer equipped with an enhanced sensitivity (HG) *Saturn724+* detector mounted at the window of an *FR-E+ SuperBright* molybdenum rotating anode generator with HF *Varimax* optics (100µm focus). **Cell determination and** **data collection**: *CrystalClear-SM Expert 3.1 b27* (Rigaku, 2013). **Data reduction, cell** **refinement and** **absorption correction**: *CrystalClear-SM Expert 3.1 b27 (Rigaku, 2013).* **Structure solution**: *SUPERFLIP* (Palatinus, L. & Chapuis, G. (2007). J. Appl. Cryst. 40, 786-790). **Structure refinement**: *SHELXL-2012* (Sheldrick, G.M. (2008). Acta Cryst. A64, 112-122). **Graphics:** *OLEX2* (Dolomanov, O. V., Bourhis, L. J., Gildea, R. J., Howard, J. A. K. & Puschmann, H. (2009). J. Appl. Cryst. 42, 339-341).

**Special details**:

The ligands exhibit 50:50 positional disorder

Figure 1 – ^1^H NMR spectrum of [3-(pyridin-2-yl)phenyl]methanol (**2**)


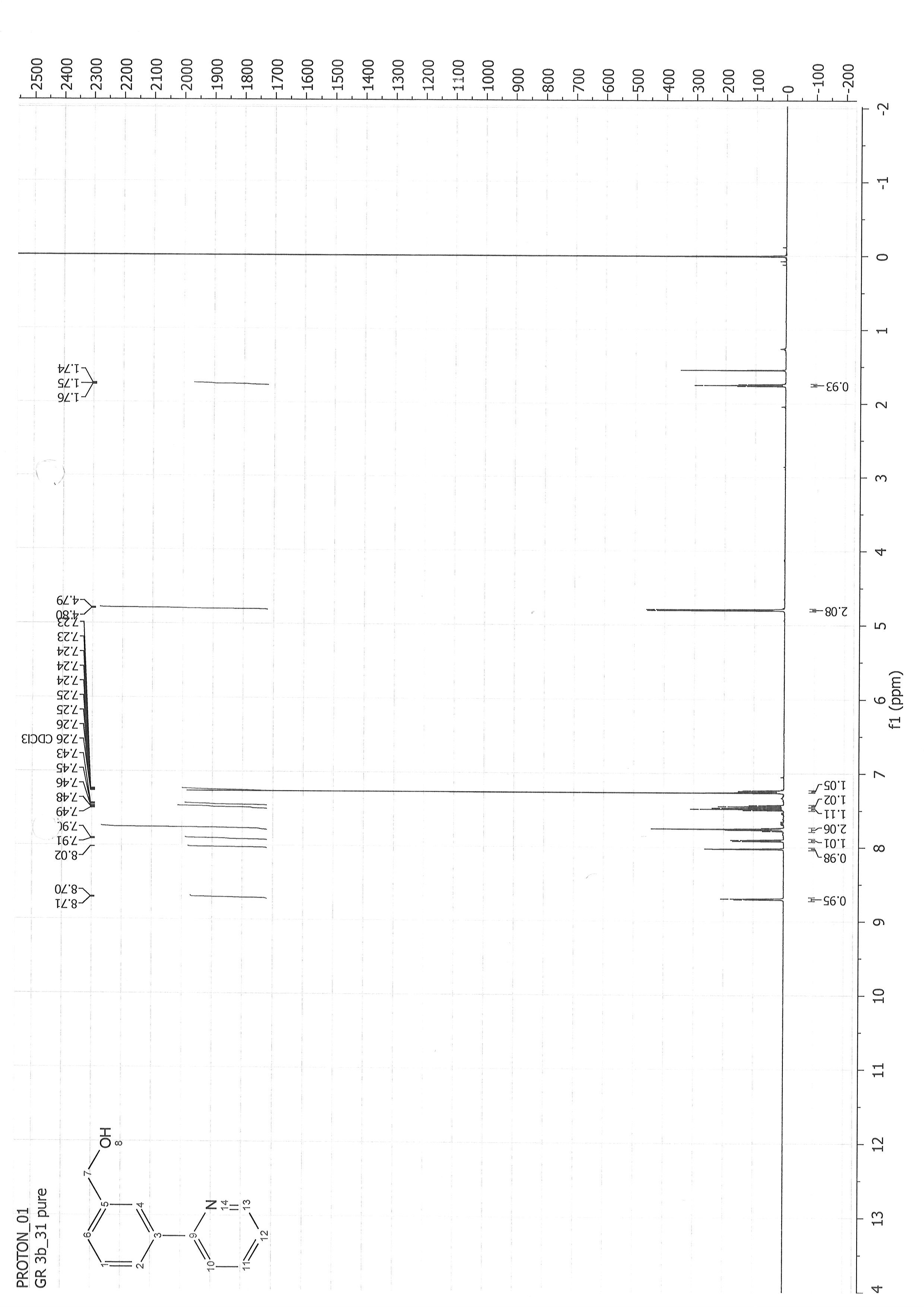


Figure 2 – ^13^C spectrum of [3-(pyridin-2-yl)phenyl]methanol (**2**)

**
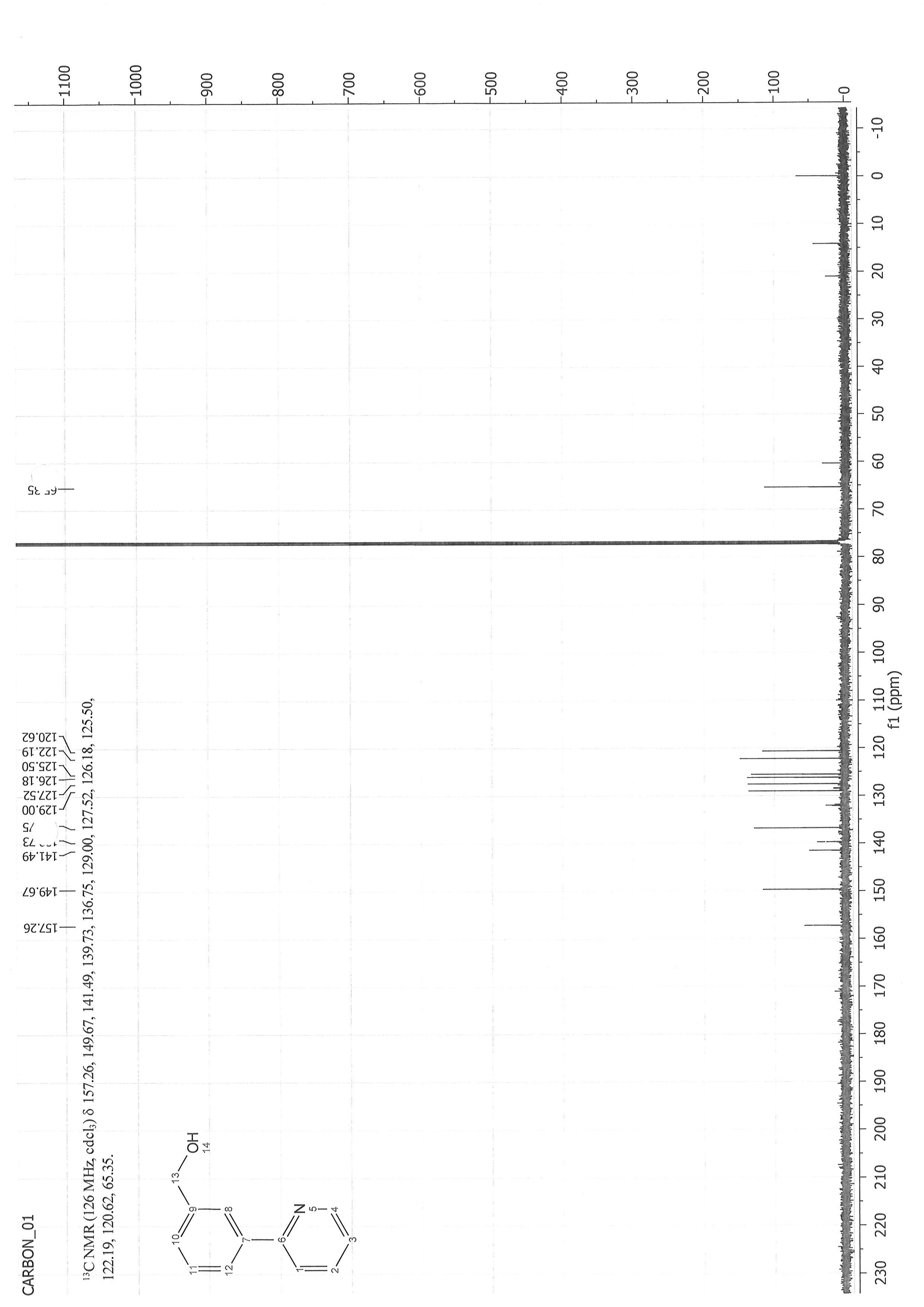
**

Figure 3 – HRMS spectrum of [3-(pyridin-2-yl)phenyl]methanol (**2**)

**
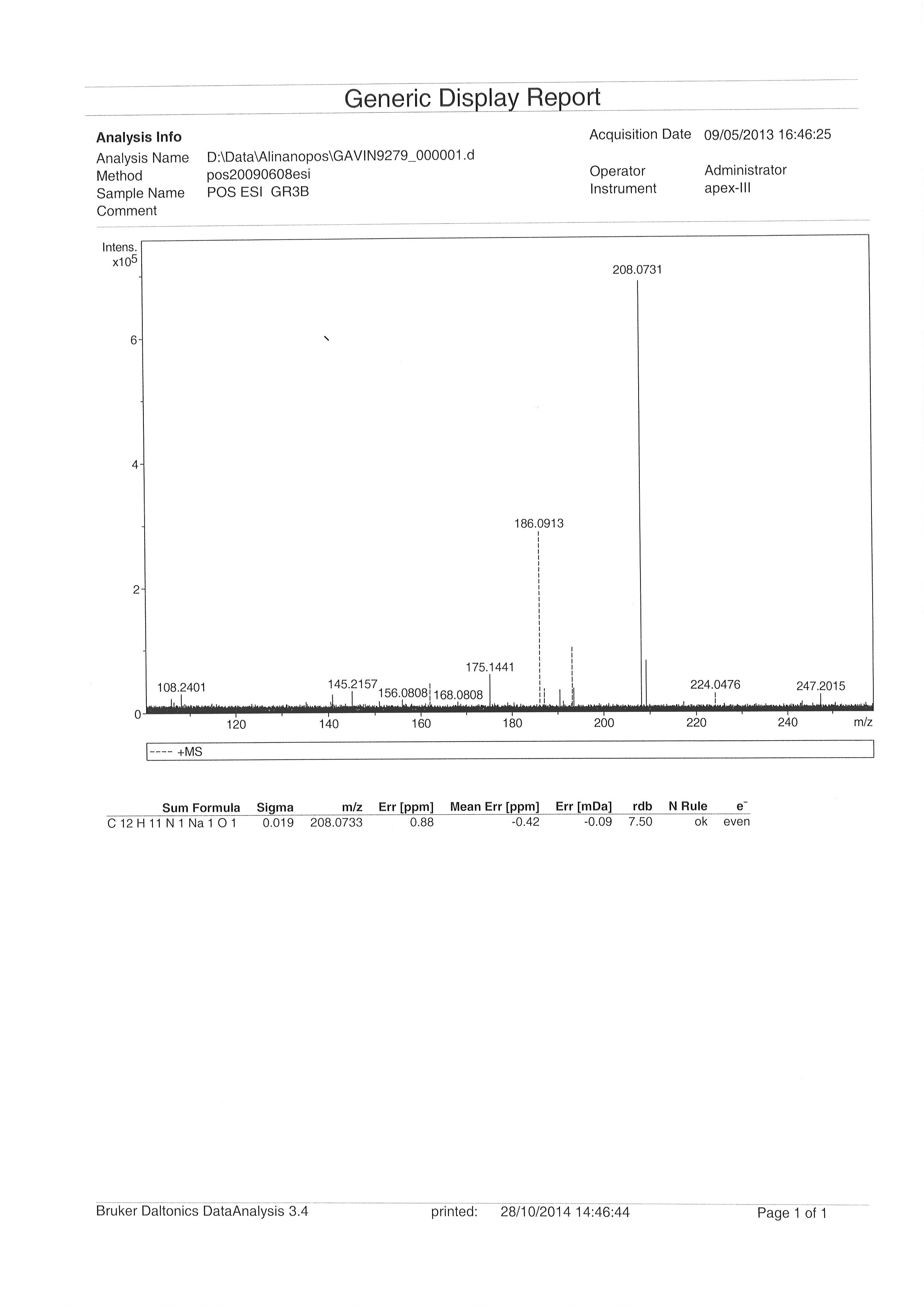
**

Figure 4 – ^1^H spectrum of 2-[3-(bromomethyl)phenyl]pyridine (**3**)


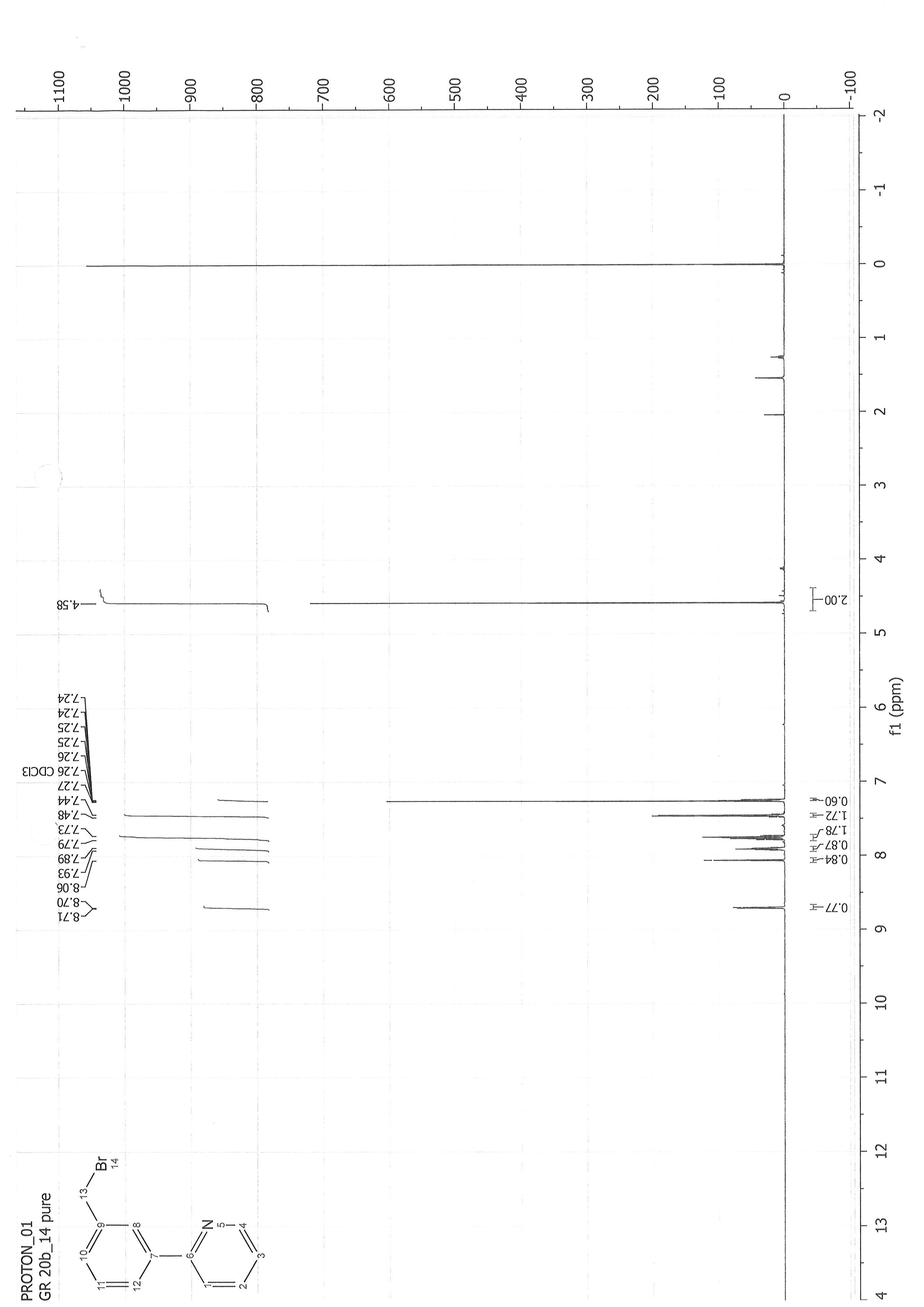


Figure 5 – ^13^C spectrum of 2-[3-(bromomethyl)phenyl]pyridine (**3**)


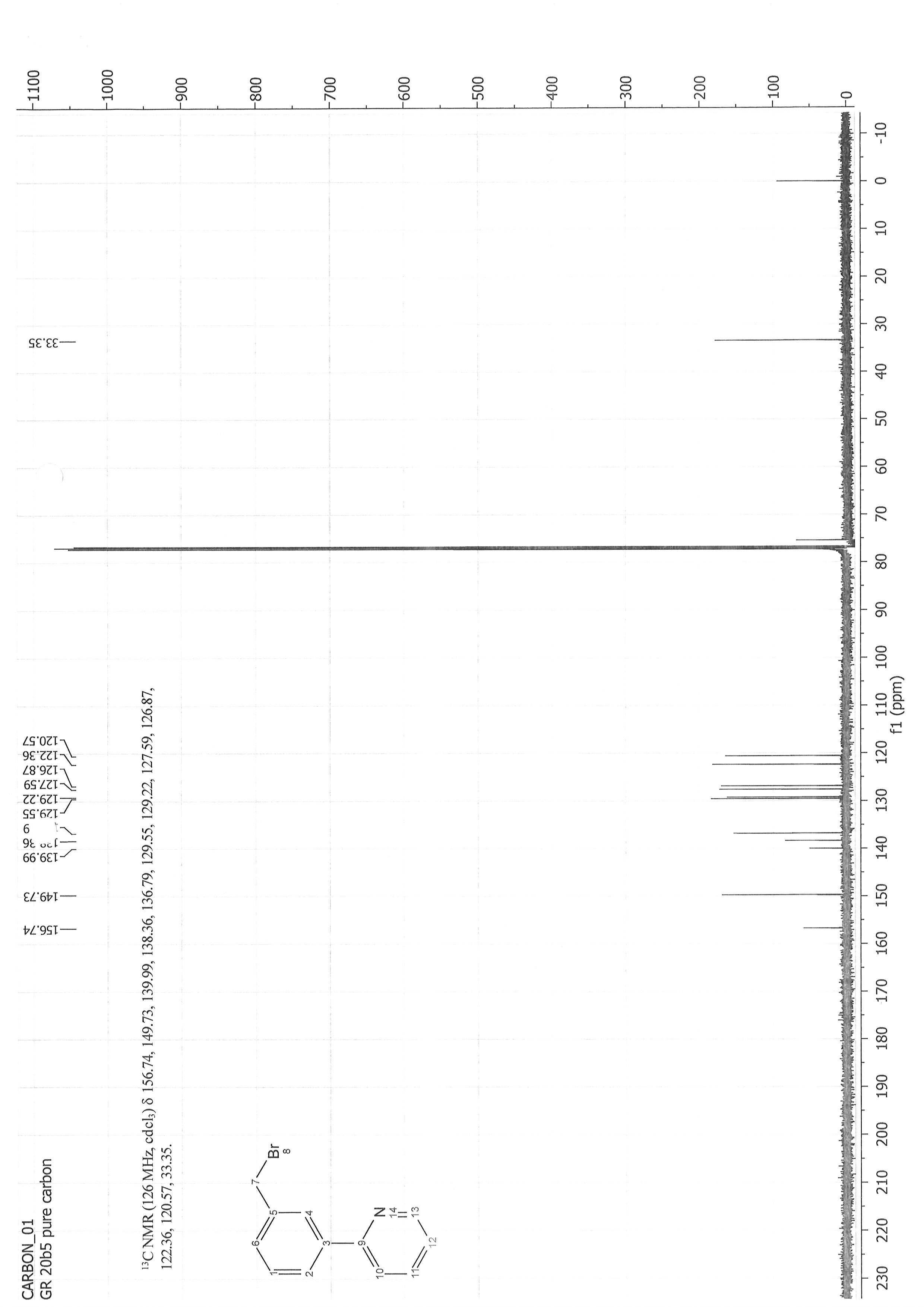


Figure 6 – HRMS of 2-[3-(bromomethyl)phenyl]pyridine (**3**)


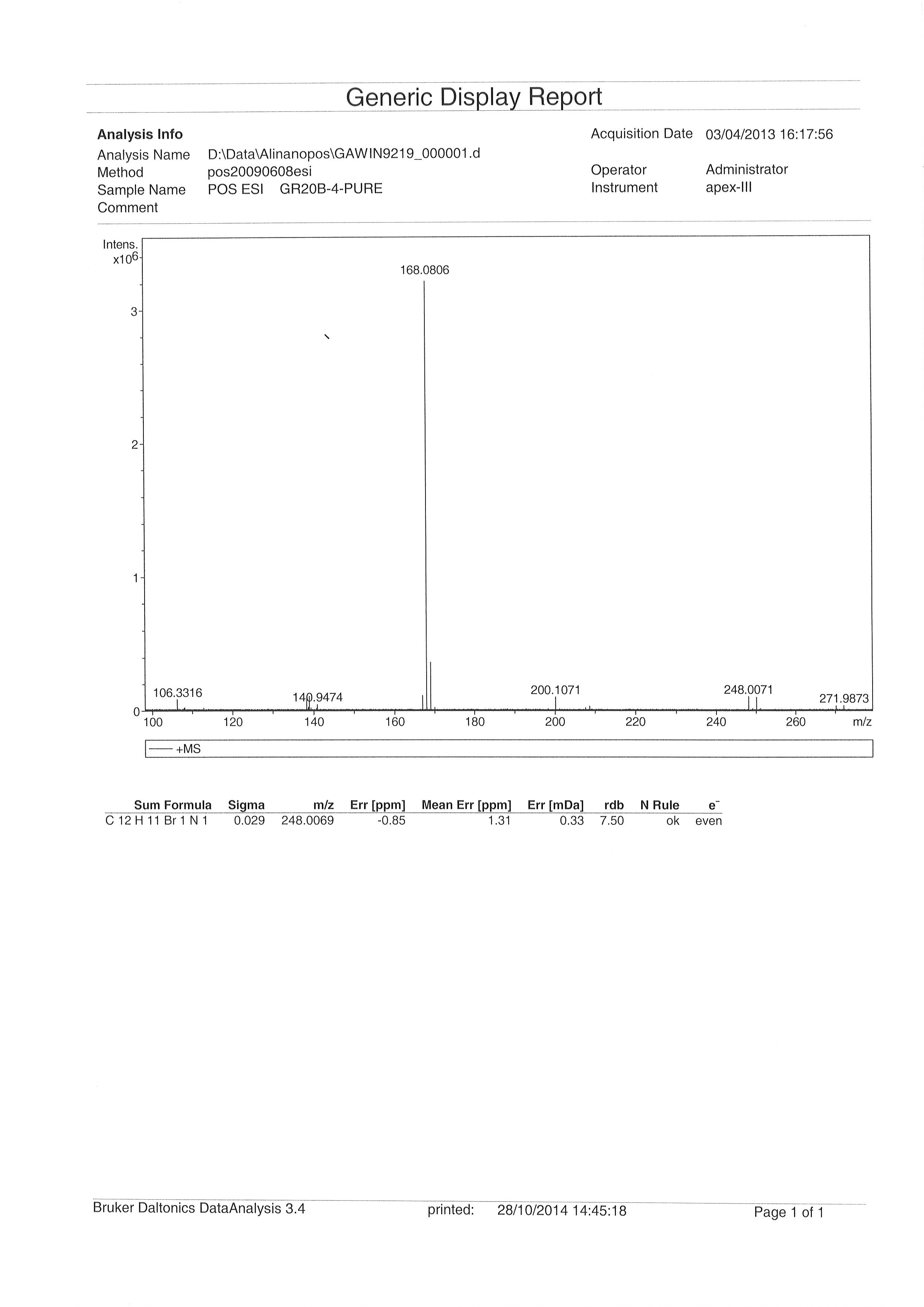


Figure 7 – ^1^H of 2-{3-[(methylsulfanyl)methyl]phenyl}pyridine (**1**)


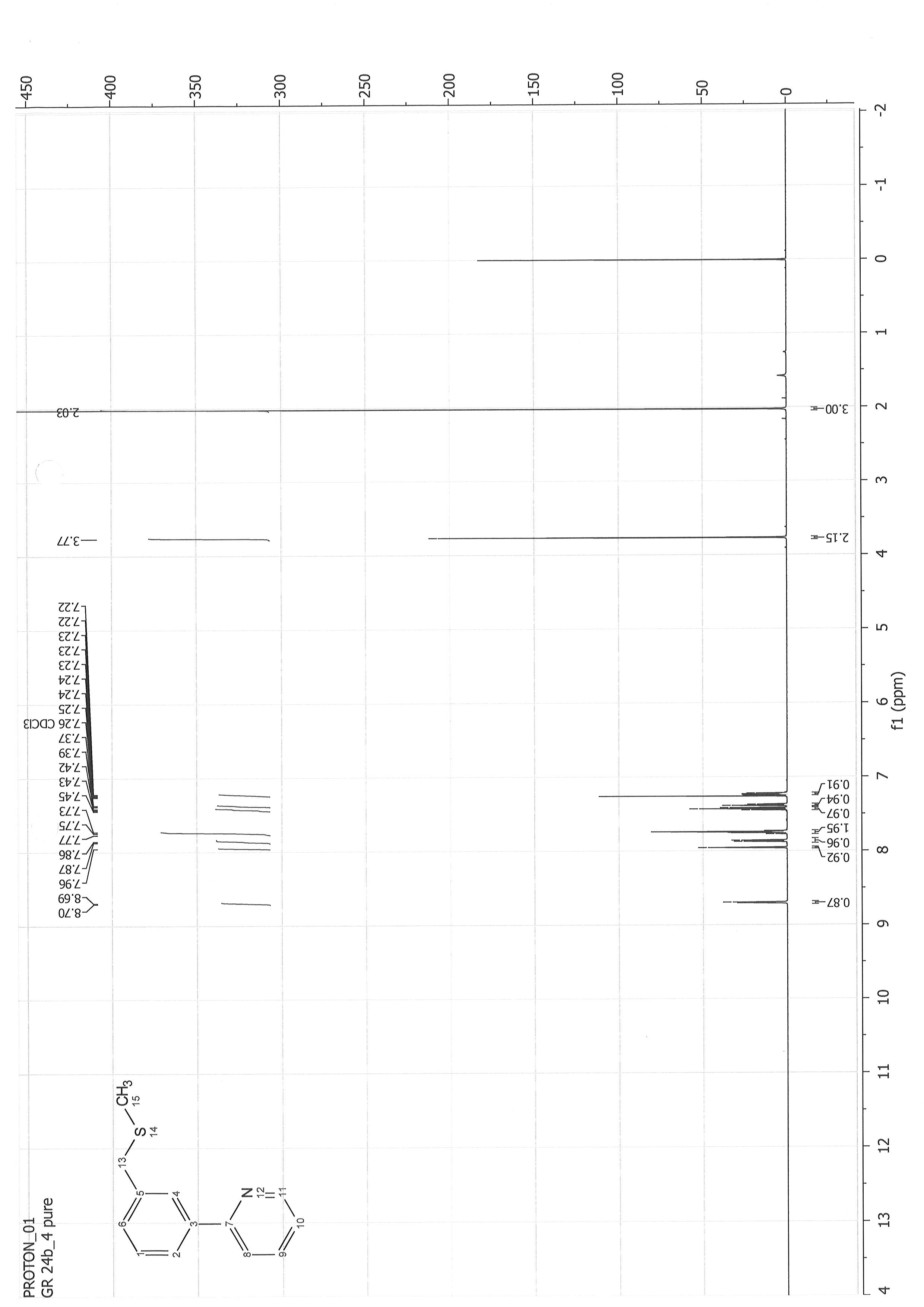


Figure 8 – ^13^C of 2-{3-[(methylsulfanyl)methyl]phenyl}pyridine (**1**)


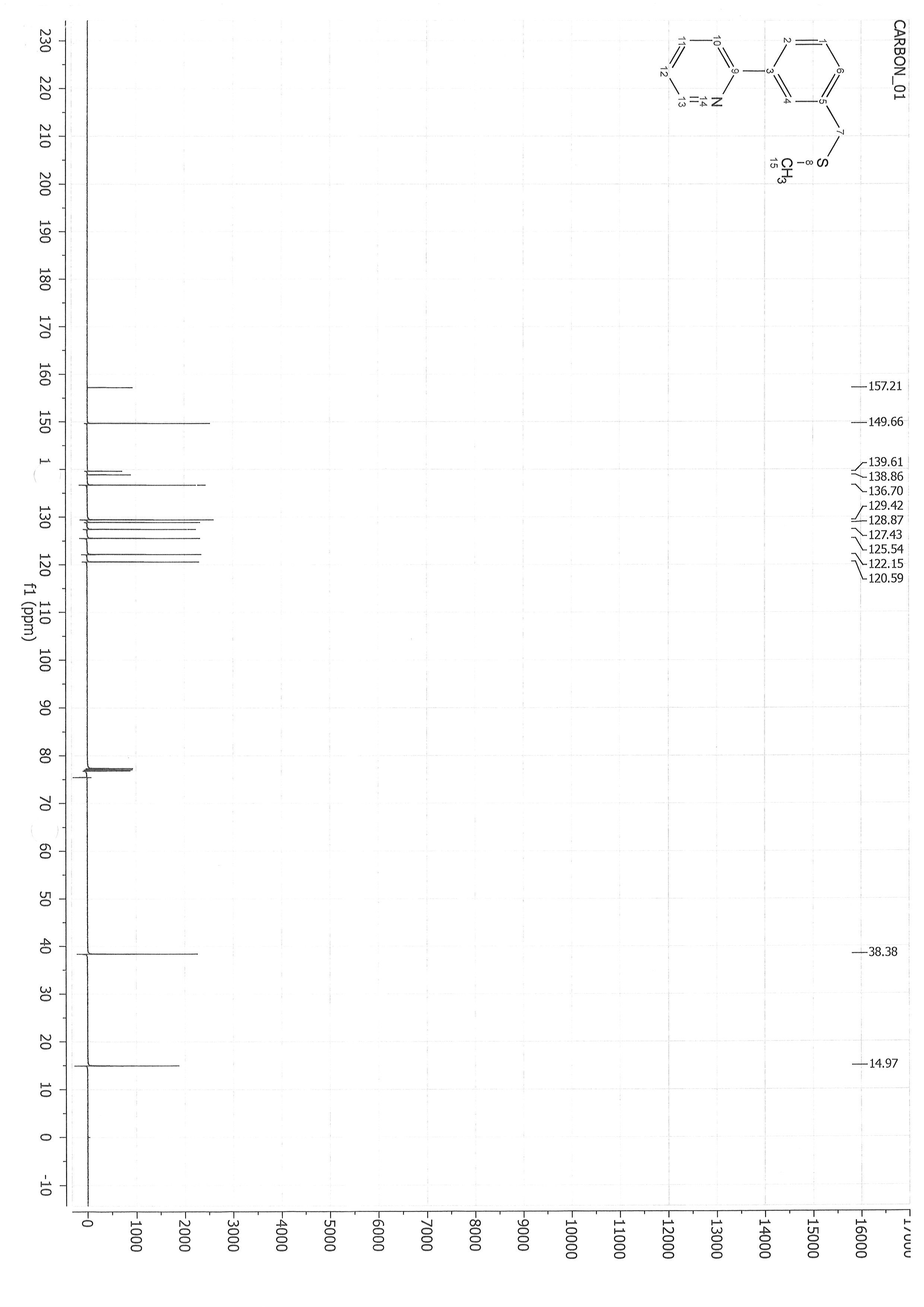


**
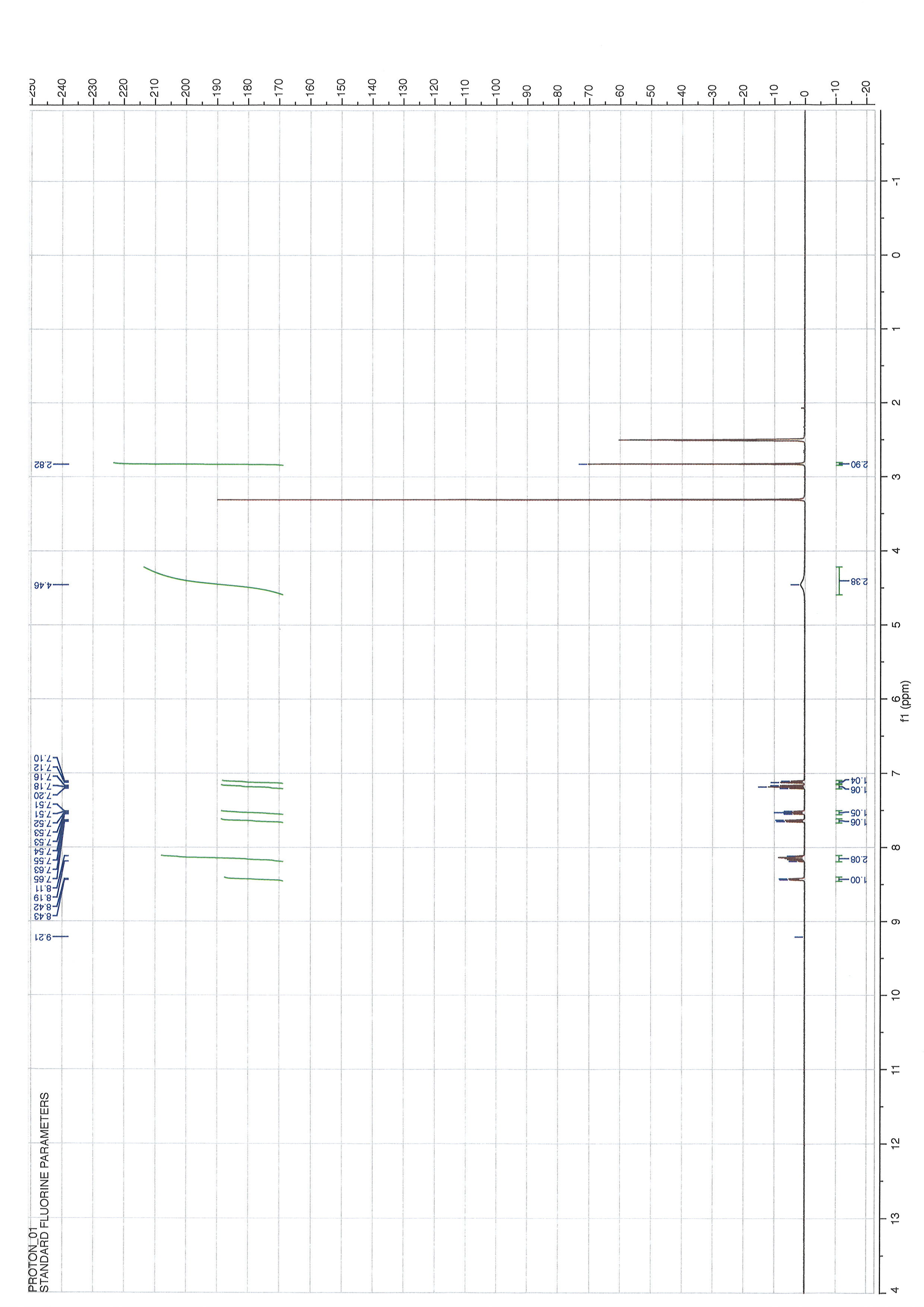
**Figure 9 – ^1^H of mixture of **4b** and **5**


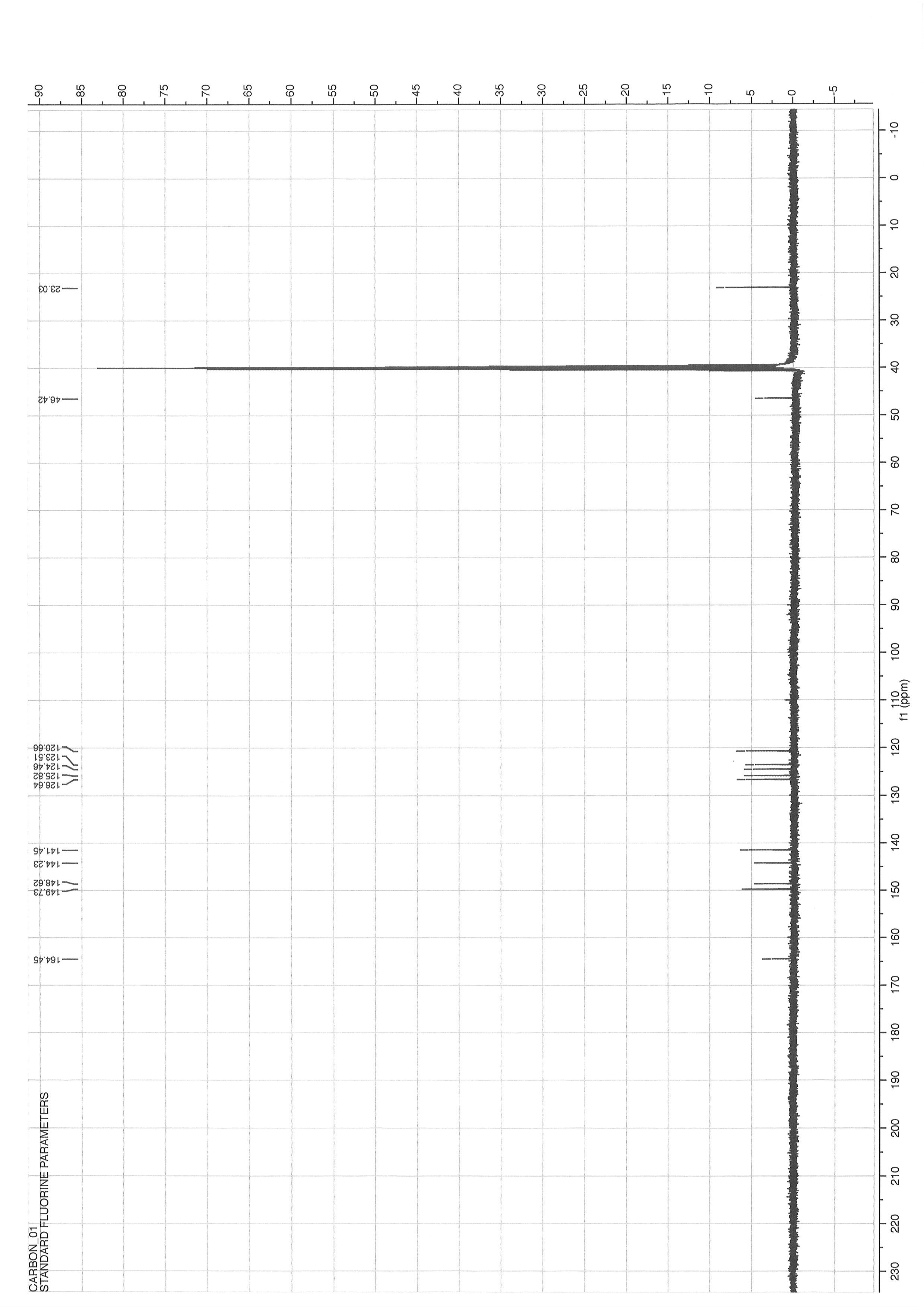
Figure 10 – ^13^C of mixture of **4b** and **5**


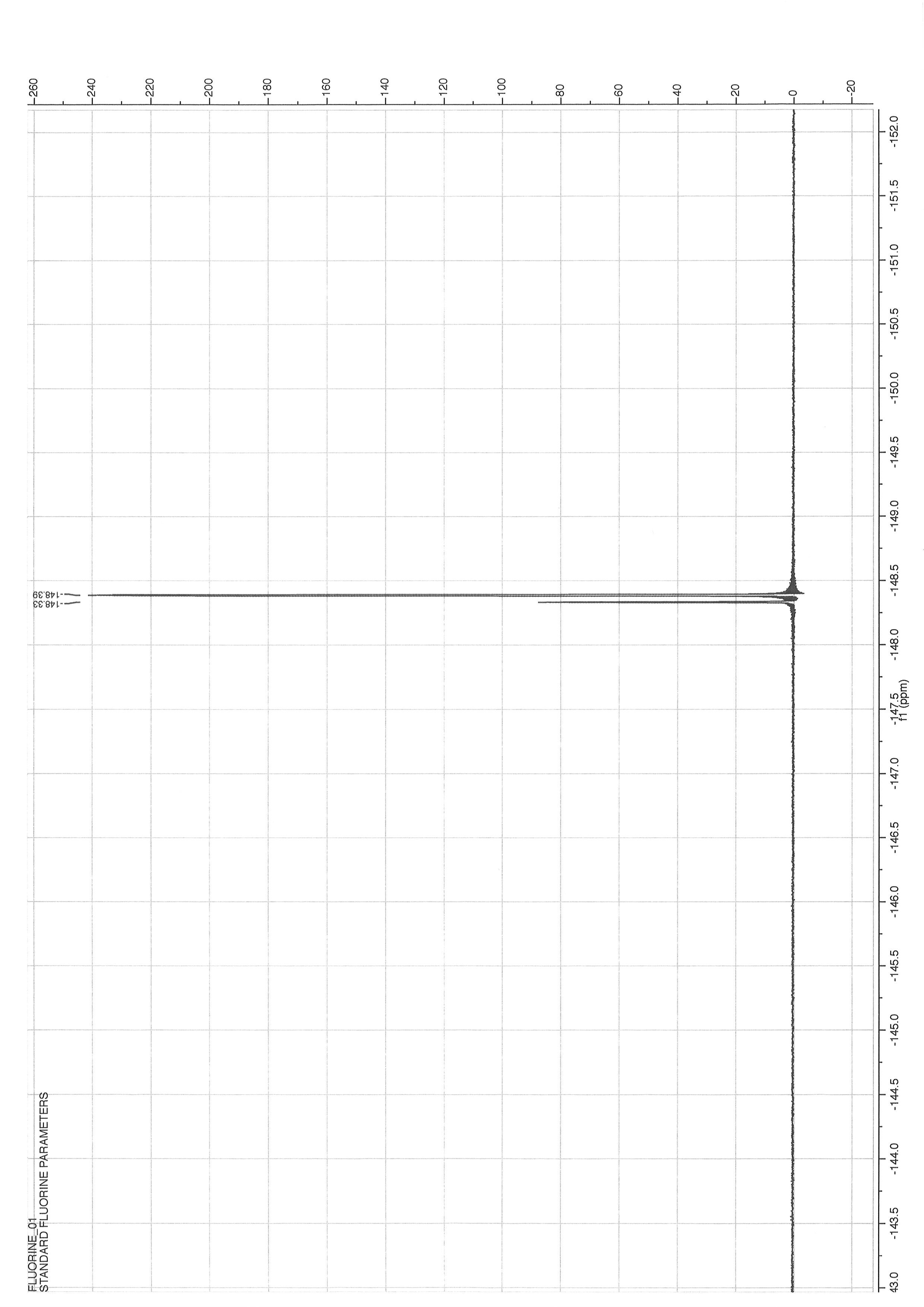
Figure 11 – ^19^F of mixture of **4b** and **5**

**
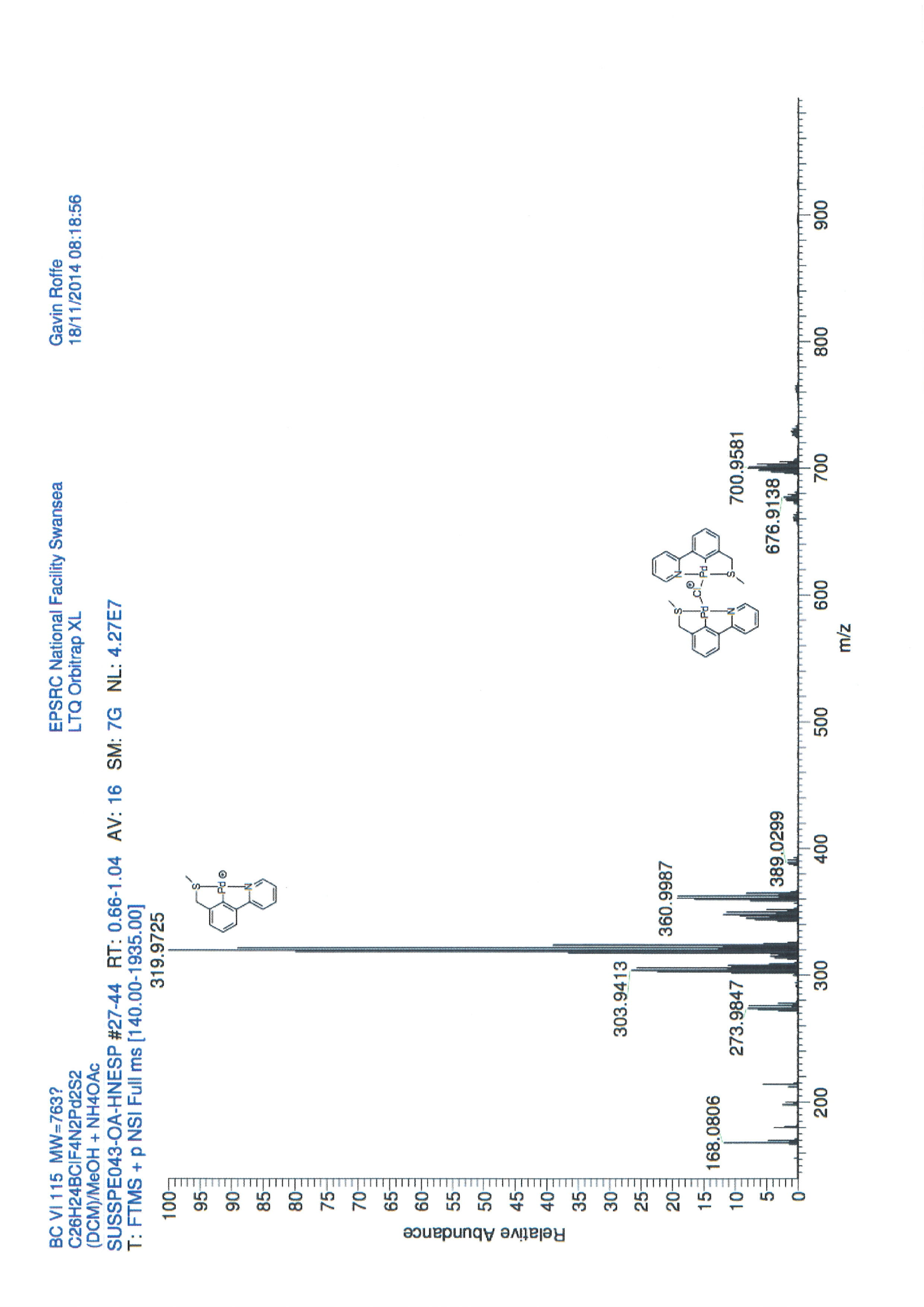
**Figure 12 – HRMS of mixture of **4b** and **5.**


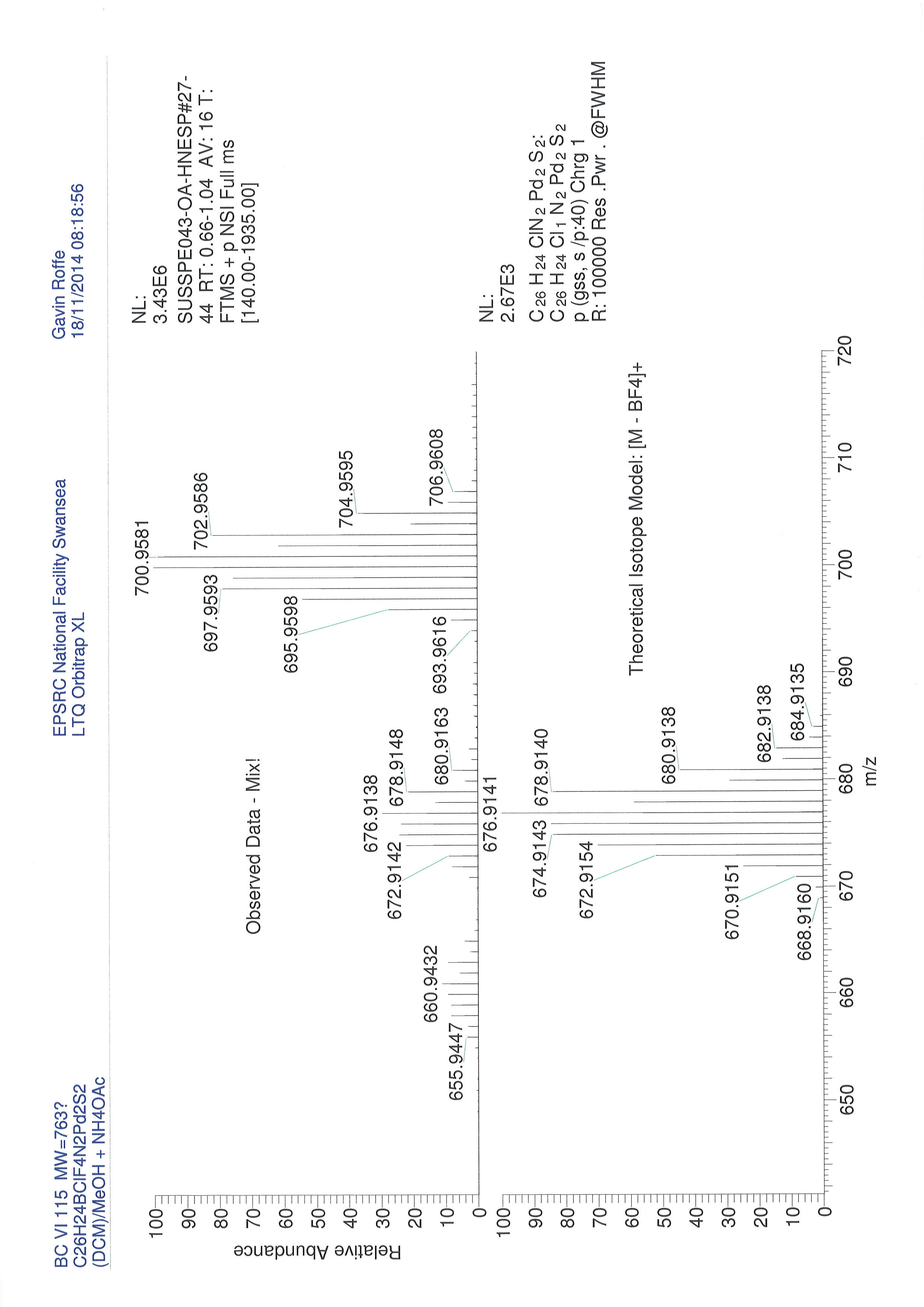


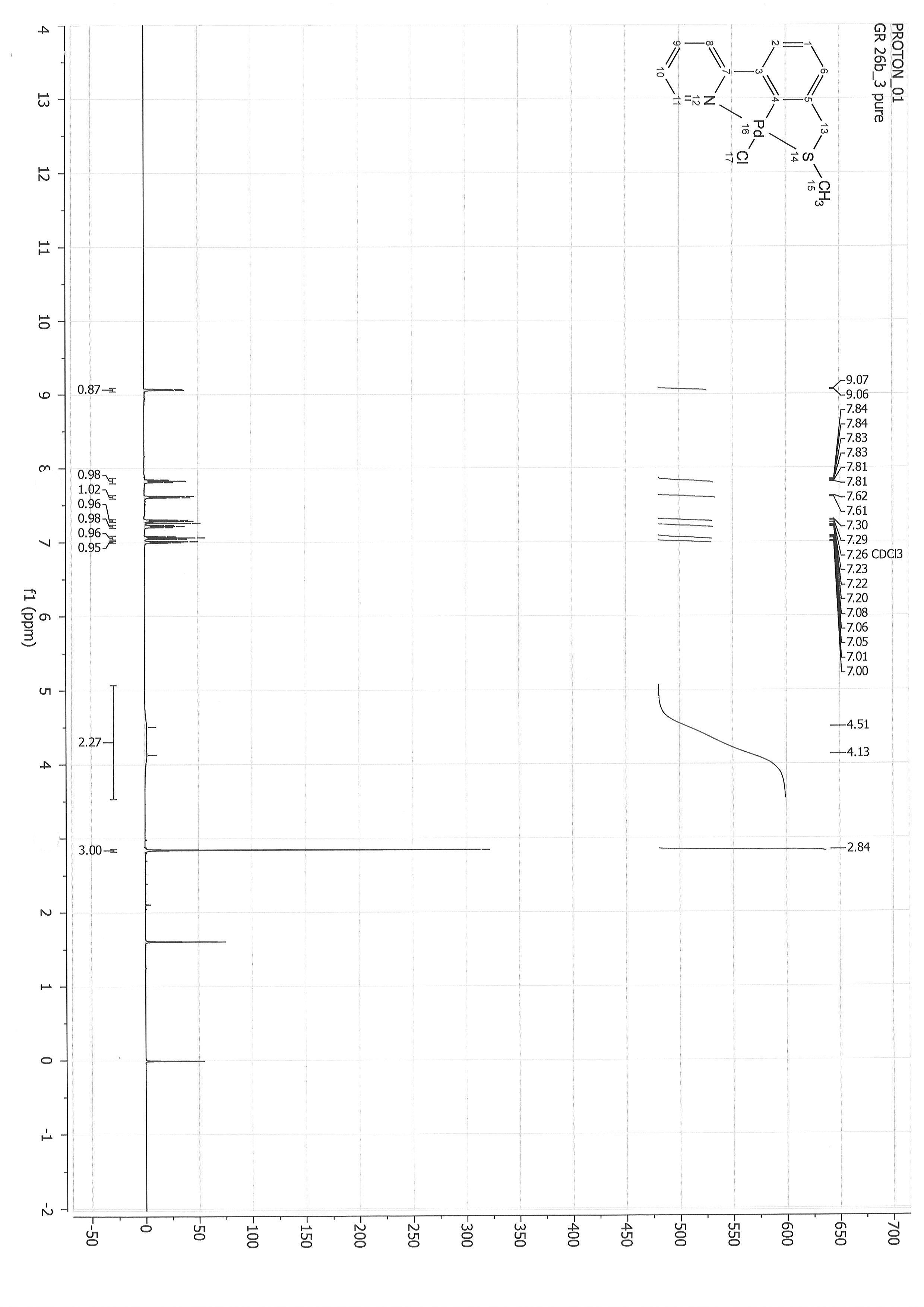
Figure 13 – ^1^H of 2-{3-[(methylsulfanyl)methyl]phenyl}pyridine chloro palladacycle (**6**)


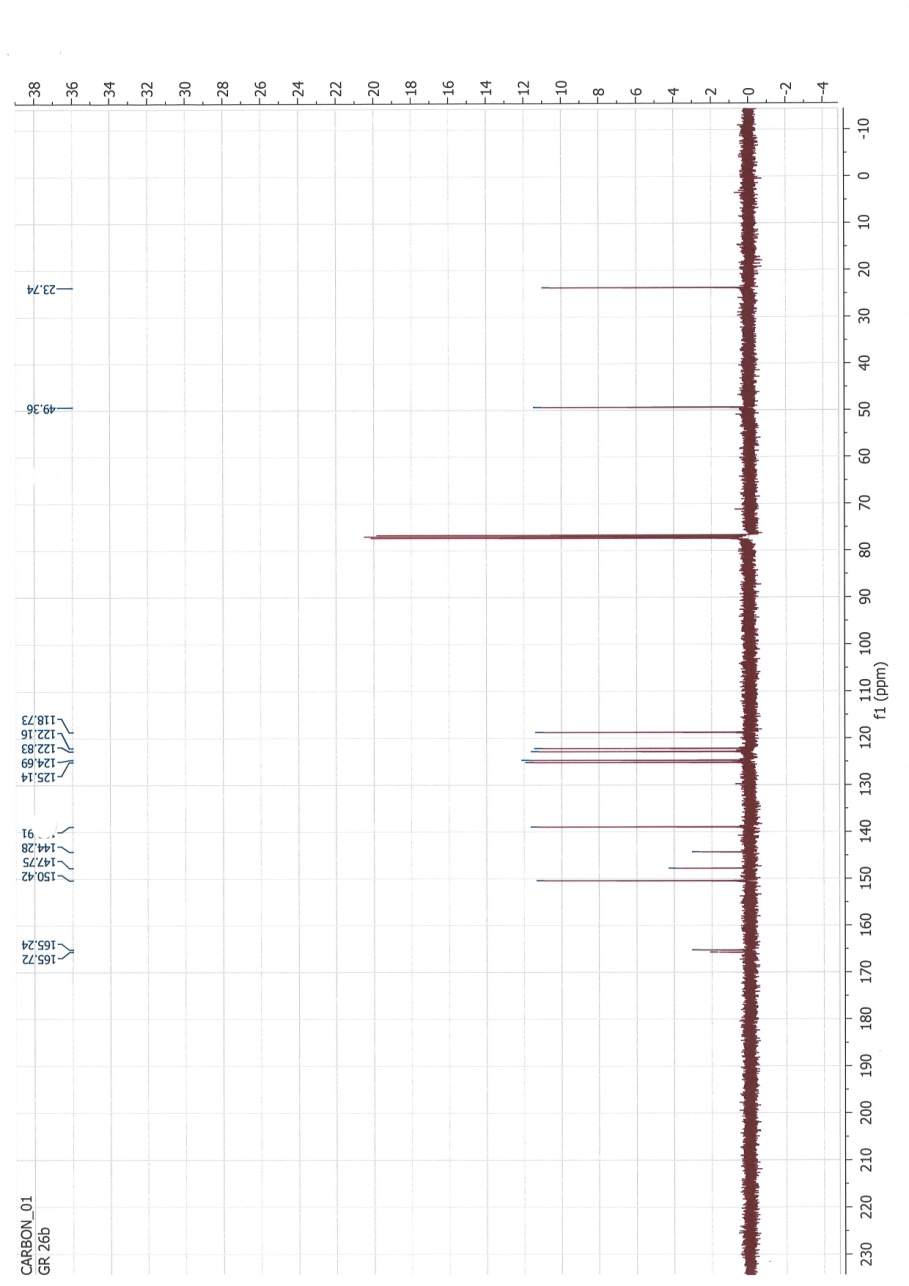
Figure 14 – ^13^C of 2-{3-[(methylsulfanyl)methyl]phenyl}pyridine chloro palladacycle (**6**)

Figure 15 – Elemental analysis of 2-{3-[(methylsulfanyl)methyl]phenyl}pyridine chloro palladacycle (**6**)


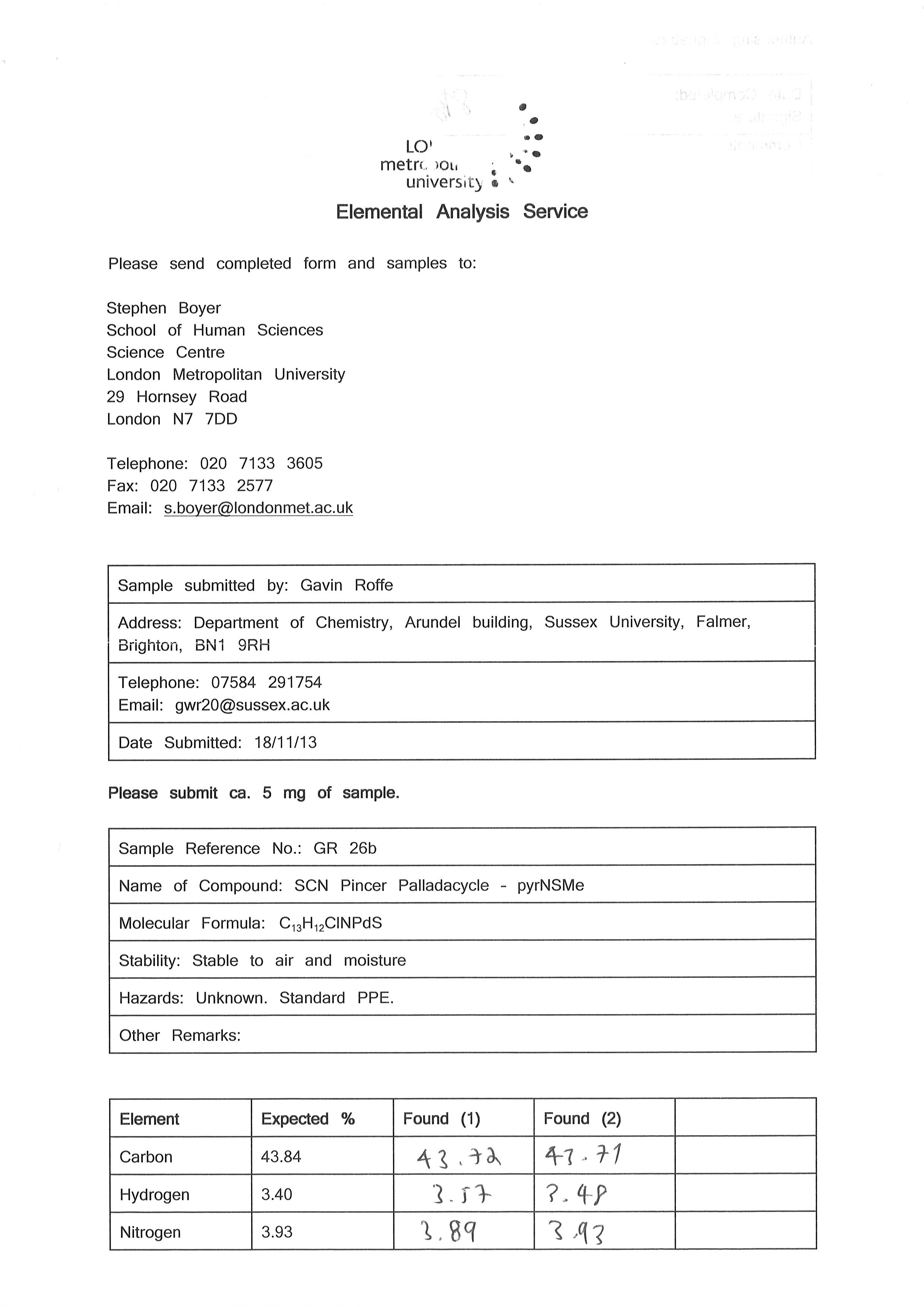


Figure 16 – HRMS of 2-{3-[(methylsulfanyl)methyl]phenyl}pyridine chloro palladacycle (**6**)


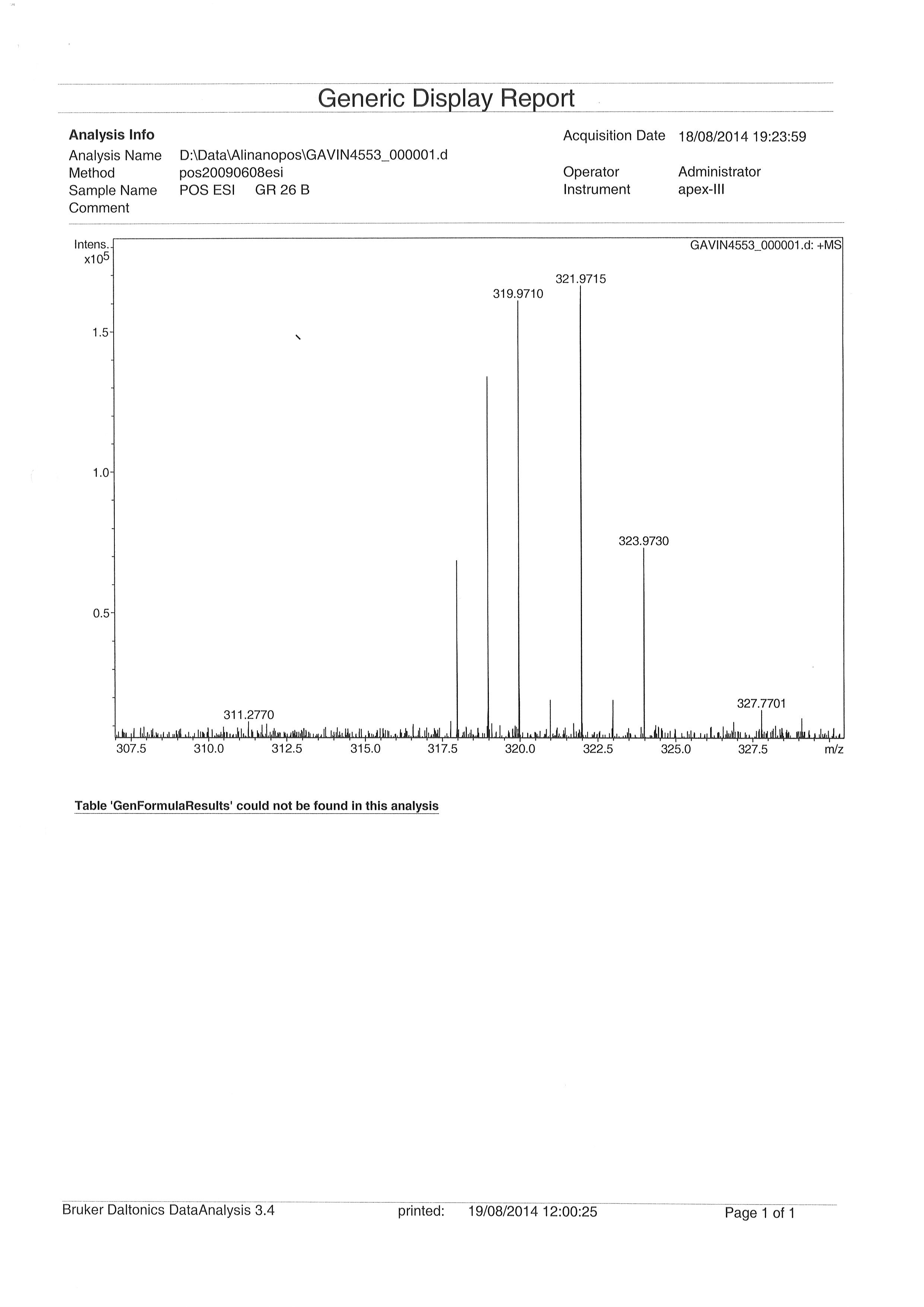


# Figure 17 – Crude ^1^H NMR and GC conversion of 2-bromo-1,3-dimethylbenzene to 1,3-dimethyl-2-phenylbenzene. Reveals a 71 % conversion from the starting 2-bromo-1,3-dimethylbenzene to 1,3-dimethyl-2-phenylbenzene, concurrent with the GC conversion of 67 %.

#

#
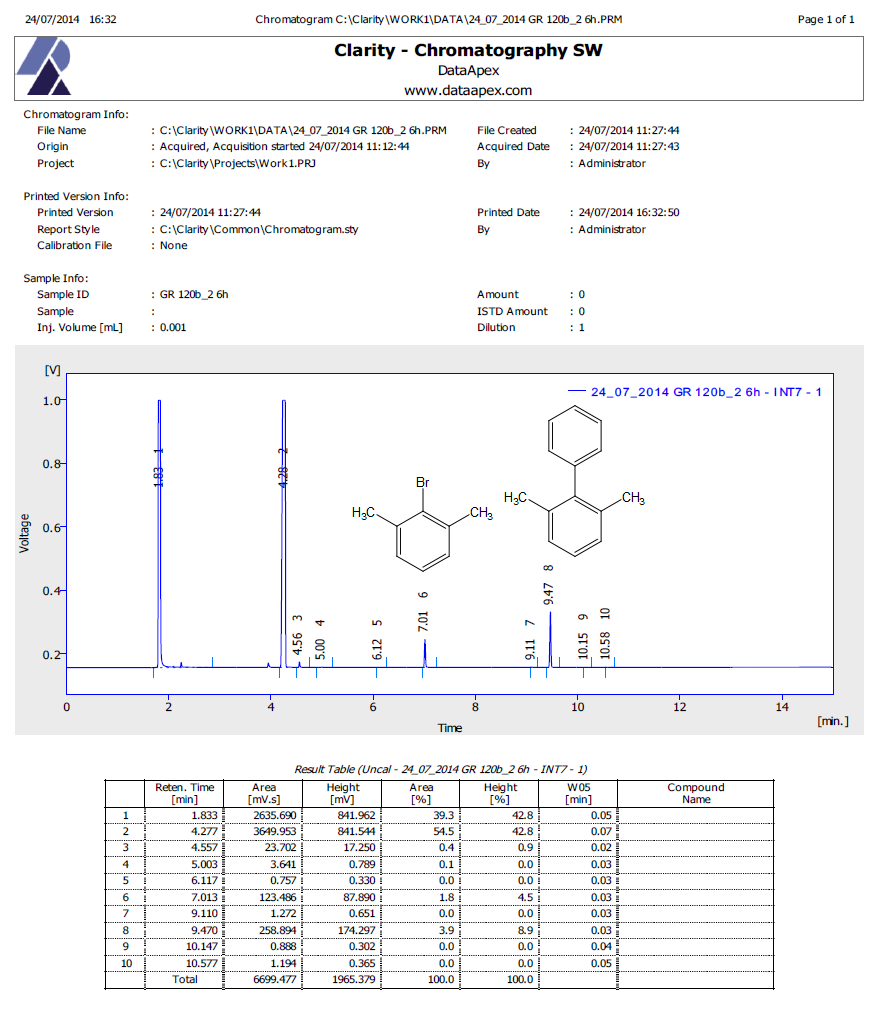


**S2**: Energies for formation reaction pathways to **III** and **6**.

**Table 1:** Zero-point energy corrected (Gibbs Free) energies for pathways to **III,** without solvent corrections.

| **Structure** | **S-coordination first** | **N-coordination first** |
| --- | --- | --- |
|  | **ΔE_0_ (ΔG) / kJmol^-1^** | |
| **Ligand + PdCl_2_** | 0.0 (0.0) | 0.0 (0.0) |
| **Int 1** | -211.7 (-155.8) | -230.6 (-170.5) |
| **TS 1-2** | -97.4 (-31.8) | -104.4 (-41.3) |
| **Int 2** | -249.2 (-185.5) | -175.4 (-110.8) |
| **TS 2-3** | -168.9 (-108.9) | -137.4 (-76.7) |
| **Int 3** | -271.8 (-208.3) | -268.4 (-207.9) |
| **III** | -243.6 (-216.8) | -243.6 (-216.9) |

**Table 2:** Zero-point energy corrected (Gibbs Free) energies for pathways to **6,** without solvent corrections.

| **Structure** | **S-coordination first** | **N-coordination first** |
| --- | --- | --- |
|  | **ΔE_0_ (ΔG) / kJmol^-1^** | |
| **Ligand + PdCl_2_** | 0.0 (0.0) | 0.0 (0.0) |
| **Int 1** | -198.8 (-141.3) | -216.9 (-161.1) |
| **TS 1-2** | -103.3 (-40.3) | -91.7 (-30.5) |
| **Int 2** | -217.4 (-153.7) | -173.2 (-112.1) |
| **TS 2-3** | -174.9 (-114.2) | -147.0 (-84.9) |
| **Int 3** | -277.6 (-218.9) | -282.9 (-226.7) |
| **2** | -250.4 (-225.3) | -247.5 (-222.2) |

**Table 3:** Acetonitrile solvent and Gibbs free corrected energies to **III**.

| **Structure** | **S-coordination first** | **N-coordination first** |
| --- | --- | --- |
|  | **ΔG / kJmol^-1^** | |
| **Ligand + PdCl_2_** | 0.0 | 0.0 |
| **Int 1** | -98.6 | -112.6 |
| **TS 1-2** | 32.1 | 31.1 |
| **Int 2** | -153.1 | -36.7 |
| **TS 2-3** | -41.2 | -16.9 |
| **Int 3** | -125.5 | -128.7 |
| **III** | -143.8 | -143.8 |

**Table 4:** Acetonitrile solvent and Gibbs free corrected energies to **6**.

| **Structure** | **S-coordination first** | **N-coordination first** |
| --- | --- | --- |
|  | **ΔG / kJmol^-1^** | |
| **Ligand + PdCl_2_** | 0.0 | 0.0 |
| **Int 1** | -91.4 | -105.2 |
| **TS 1-2** | 34.7 | 48.9 |
| **Int 2** | -113.5 | -31.8 |
| **TS 2-3** | -62.4 | -2.1 |
| **Int 3** | -135.5 | -143.7 |
| **III** | -150.8 | -150.3 |

**S3:** Bader charge and AIM analysis data for formation pathway to **III** for S-coordination first for key atoms and bonds.

**(1) Ligand charges**

| Atom | Charge /au |
| --- | --- |
| S | -0.005 |
| N | -0.954 |
| C | -0.026 |
| H | 0.041 |

**(2) Ligand AIM data**

| Bond | *ρ*(**r**) | ∇^2^*ρ*(**r**) | *H*(**r**) |
| --- | --- | --- | --- |
| C-H | 0.287 | -1.073 | -0.306 |

**(3) Int 1 charges**

| Atom | Charge /au |
| --- | --- |
| Pd | 0.620 |
| S | 0.116 |
| N | -0.969 |
| C | -0.105 |
| H | 0.071 |

**(4) Int 1 AIM data**

| Bond | *ρ*(**r**) | ∇^2^*ρ*(**r**) | *H*(**r**) |
| --- | --- | --- | --- |
| Pd-S | 0.086 | 0.237 | -0.022 |
| Pd-C | 0.038 | 0.101 | -0.007 |
| Pd-Cl (cleaving) | 0.090 | 0.263 | -0.022 |
| C-H | 0.285 | -1.050 | -0.301 |

**(5) TS 1-2 charges**

| Atom | Charge /au |
| --- | --- |
| Pd | 0.607 |
| S | 0.099 |
| N | -0.975 |
| C | -0.297 |
| H | 0.327 |
| Cl | -0.548 |

**(6) TS 1-2 AIM data**

| Bond | *ρ*(**r**) | ∇^2^*ρ*(**r**) | *H*(**r**) |
| --- | --- | --- | --- |
| Pd-S | 0.085 | 0.213 | -0.023 |
| Pd-C | 0.057 | 0.162 | -0.011 |
| Pd-Cl (cleaving) | 0.084 | 0.242 | -0.020 |
| C-H | 0.136 | -0.207 | -0.085 |
| N-H | 0.107 | -0.042 | -0.057 |

**(7) Int 2 charges**

| Atom | Charge /au |
| --- | --- |
| Pd | 0.557 |
| S | 0.115 |
| N | -0.984 |
| C | -0.141 |
| H | 0.491 |
| Cl | -0.617 |

**(8) Int 2 AIM data**

| Bond | *ρ*(**r**) | ∇^2^*ρ*(**r**) | *H*(**r**) |
| --- | --- | --- | --- |
| Pd-S | 0.093 | 0.246 | -0.026 |
| Pd-C | 0.123 | 0.236 | -0.045 |
| Pd-Cl (cleaving) | 0.069 | 0.234 | -0.013 |
| N-H | 0.297 | -1.572 | -0.447 |

**(9) TS 2-3 charges**

| Atom | Charge /au |
| --- | --- |
| Pd | 0.536 |
| S | 0.145 |
| N | -0.966 |
| C | -0.144 |
| H | 0.351 |
| Cl | -0.416 |

**(10) TS 2-3 AIM data**

| Bond | *ρ*(**r**) | ∇^2^*ρ*(**r**) | *H*(**r**) |
| --- | --- | --- | --- |
| Pd-S | 0.102 | 0.254 | -0.031 |
| Pd-C | 0.144 | 0.193 | -0.062 |
| Pd-N | 0.014 | 0.049 | 0.000 |
| Pd-H | 0.014 | 0.050 | 0.001 |
| N-H | 0.044 | 0.072 | -0.006 |

**(11) Int 3 charges**

| Atom | Charge /au |
| --- | --- |
| Pd | 0.517 |
| S | 0.092 |
| N | -0.912 |
| C | -0.149 |
| H | 0.286 |
| Cl | -0.325 |

**(12) Int 3 AIM data**

| Bond | *ρ*(**r**) | ∇^2^*ρ*(**r**) | *H*(**r**) |
| --- | --- | --- | --- |
| Pd-S | 0.093 | 0.254 | -0.026 |
| Pd-C | 0.146 | 0.220 | -0.062 |
| Pd-N | 0.085 | 0.371 | -0.014 |
| Pd-H | 0.026 | 0.049 | -0.003 |

**(13) Product charges**

| Atom | Charge /au |
| --- | --- |
| Pd | 0.539 |
| S | 0.098 |
| N | -0.912 |
| C | -0.143 |

**(14) Product AIM data**

| Bond | *ρ*(**r**) | ∇^2^*ρ*(**r**) | *H*(**r**) |
| --- | --- | --- | --- |
| Pd-S | 0.093 | 0.260 | -0.026 |
| Pd-C | 0.147 | 0.228 | -0.062 |
| Pd-N | 0.084 | 0.374 | -0.013 |

**S4:** Bader charge and AIM analysis data for formation pathway to **III** for N- coordination first for key atoms and bonds.

**(1) Ligand charges**

| Atom | Charge /au |
| --- | --- |
| S | -0.005 |
| N | -0.954 |
| C | -0.026 |
| H | 0.040 |

**(2) Ligand AIM data**

| Bond | *ρ*(**r**) | ∇^2^*ρ*(**r**) | *H*(**r**) |
| --- | --- | --- | --- |
| C-H | 0.287 | -1.073 | -0.306 |

**(3) Int 1 charges**

| Atom | Charge /au |
| --- | --- |
| Pd | 0.689 |
| S | -0.016 |
| N | -0.925 |
| C | -0.077 |
| H | 0.060 |

**(4) Int 1 AIM data**

| Bond | *ρ*(**r**) | ∇^2^*ρ*(**r**) | *H*(**r**) |
| --- | --- | --- | --- |
| Pd-N | 0.090 | 0.387 | -0.015 |
| Pd-C | 0.049 | 0.134 | -0.009 |
| Pd-Cl (cleaving) | 0.090 | 0.257 | -0.022 |
| C-H | 0.287 | -1.070 | -0.305 |

**(5) TS 1-2 charges**

| Atom | Charge /au |
| --- | --- |
| Pd | 0.669 |
| S | -0.002 |
| N | -0.903 |
| C | -0.265 |
| H | 0.328 |
| Cl | -0.401 |

**(6) TS 1-2 AIM data**

| Bond | *ρ*(**r**) | ∇^2^*ρ*(**r**) | *H*(**r**) |
| --- | --- | --- | --- |
| Pd-N | 0.095 | 0.396 | -0.018 |
| Pd-C | 0.108 | 0.210 | -0.037 |
| Pd-Cl (cleaving) | 0.067 | 0.228 | -0.014 |
| C-H | 0.088 | -0.031 | -0.039 |
| S-H | 0.012 | 0.042 | 0.002 |

**(7) Int 2 charges**

| Atom | Charge /au |
| --- | --- |
| Pd | 0.627 |
| S | 0.006 |
| N | -0.900 |
| C | -0.160 |
| H | 0.346 |
| Cl | -0.350 |

**(8) Int 2 AIM data**

| Bond | *ρ*(**r**) | ∇^2^*ρ*(**r**) | *H*(**r**) |
| --- | --- | --- | --- |
| Pd-N | 0.095 | 0.403 | -0.018 |
| Pd-C | 0.128 | 0.215 | -0.048 |
| Pd-Cl (cleaving) | 0.061 | 0.234 | -0.010 |
| S-H | 0.061 | 0.017 | -0.020 |

**(9) TS 2-3 charges**

| Atom | Charge /au |
| --- | --- |
| Pd | 0.621 |
| S | -0.020 |
| N | -0.894 |
| C | -0.137 |
| H | 0.330 |
| Cl | -0.377 |

**(10) TS 2-3 AIM data**

| Bond | *ρ*(**r**) | ∇^2^*ρ*(**r**) | *H*(**r**) |
| --- | --- | --- | --- |
| Pd-N | 0.094 | 0.393 | -0.018 |
| Pd-C | 0.147 | 0.189 | -0.063 |
| Pd-Cl | 0.021 | 0.066 | -0.001 |
| S-H | 0.040 | 0.041 | -0.008 |

**(11) Int 3 charges**

| Atom | Charge /au |
| --- | --- |
| Pd | 0.523 |
| S | 0.098 |
| N | -0.917 |
| C | -0.153 |
| H | 0.280 |
| Cl | -0.321 |

**(12) Int 3 AIM data**

| Bond | *ρ*(**r**) | ∇^2^*ρ*(**r**) | *H*(**r**) |
| --- | --- | --- | --- |
| Pd-N | 0.086 | 0.375 | -0.014 |
| Pd-C | 0.146 | 0.221 | -0.062 |
| Pd-S | 0.093 | 0.256 | -0.026 |
| Pd-H | 0.025 | 0.050 | -0.003 |

**(13) Product charges**

| Atom | Charge /au |
| --- | --- |
| Pd | 0.539 |
| S | 0.098 |
| N | -0.912 |
| C | -0.143 |

**(14) Product AIM data**

| Bond | *ρ*(**r**) | ∇^2^*ρ*(**r**) | *H*(**r**) |
| --- | --- | --- | --- |
| Pd-N | 0.084 | 0.374 | -0.013 |
| Pd-C | 0.147 | 0.228 | -0.062 |
| Pd-S | 0.093 | 0.260 | -0.026 |

**S5:** Bader charge and AIM analysis data for formation pathway to **6** for S-coordination first for key atoms and bonds.

**(1) Ligand charges**

| Atom | Charge /au |
| --- | --- |
| S | -0.009 |
| N | -1.135 |
| C | -0.032 |
| H | 0.009 |

**(2) Ligand AIM data**

| Bond | *ρ*(**r**) | ∇^2^*ρ*(**r**) | *H*(**r**) |
| --- | --- | --- | --- |
| C-H | 0.286 | -1.062 | -0.306 |

**(3) Int 1 charges**

| Atom | Charge /au |
| --- | --- |
| Pd | 0.610 |
| S | 0.117 |
| N | -1.135 |
| C | -0.064 |
| H | 0.029 |

**(4) Int 1 AIM data**

| Bond | *ρ*(**r**) | ∇^2^*ρ*(**r**) | *H*(**r**) |
| --- | --- | --- | --- |
| Pd-S | 0.087 | 0.237 | -0.023 |
| Pd-C | 0.042 | 0.112 | -0.007 |
| Pd-Cl (cleaving) | 0.091 | 0.260 | -0.023 |
| C-H | 0.287 | -1.065 | -0.306 |

**(5) TS 1-2 charges**

| Atom | Charge /au |
| --- | --- |
| Pd | 0.587 |
| S | 0.122 |
| N | -1.132 |
| C | -0.274 |
| H | 0.328 |
| Cl | -0.434 |

**(6) TS 1-2 AIM data**

| Bond | *ρ*(**r**) | ∇^2^*ρ*(**r**) | *H*(**r**) |
| --- | --- | --- | --- |
| Pd-S | 0.094 | 0.231 | -0.027 |
| Pd-C | 0.105 | 0.211 | -0.035 |
| Pd-Cl (cleaving) | 0.063 | 0.206 | -0.013 |
| C-H | 0.108 | -0.090 | -0.057 |

**(7) Int 2 charges**

| Atom | Charge /au |
| --- | --- |
| Pd | 0.565 |
| S | 0.104 |
| N | -1.274 |
| C | -0.126 |
| H | 0.528 |
| Cl | -0.608 |

**(8) Int 2 AIM data**

| Bond | *ρ*(**r**) | ∇^2^*ρ*(**r**) | *H*(**r**) |
| --- | --- | --- | --- |
| Pd-S | 0.090 | 0.238 | -0.025 |
| Pd-C | 0.123 | 0.247 | -0.044 |
| Pd-Cl (cleaving) | 0.070 | 0.231 | -0.014 |
| N-H | 0.295 | -1.690 | -0.474 |

**(9) TS 2-3 charges**

| Atom | Charge /au |
| --- | --- |
| Pd | 0.570 |
| S | 0.137 |
| N | -1.233 |
| C | -0.120 |
| H | 0.483 |
| Cl | -0.620 |

**(10) TS 2-3 AIM data**

| Bond | *ρ*(**r**) | ∇^2^*ρ*(**r**) | *H*(**r**) |
| --- | --- | --- | --- |
| Pd-S | 0.098 | 0.246 | -0.029 |
| Pd-C | 0.141 | 0.217 | -0.058 |
| Pd-N | 0.025 | 0.095 | -0.002 |
| Pd-Cl | 0.023 | 0.076 | -0.002 |
| N-H | 0.153 | -0.246 | -0.121 |

**(11) Int 3 charges**

| Atom | Charge /au |
| --- | --- |
| Pd | 0.554 |
| S | 0.094 |
| N | -1.157 |
| C | -0.133 |
| H | 0.285 |
| Cl | -0.325 |

**(12) Int 3 AIM data**

| Bond | *ρ*(**r**) | ∇^2^*ρ*(**r**) | *H*(**r**) |
| --- | --- | --- | --- |
| Pd-S | 0.093 | 0.257 | -0.026 |
| Pd-C | 0.147 | 0.231 | -0.062 |
| Pd-N | 0.097 | 0.453 | -0.018 |
| Pd-H | 0.025 | 0.049 | -0.003 |

**(13) Product charges**

| Atom | Charge /au |
| --- | --- |
| Pd | 0.575 |
| S | 0.098 |
| N | -1.154 |
| C | -0.125 |

**(14) Product AIM data**

| Bond | *ρ*(**r**) | ∇^2^*ρ*(**r**) | *H*(**r**) |
| --- | --- | --- | --- |
| Pd-S | 0.093 | 0.264 | -0.026 |
| Pd-C | 0.149 | 0.238 | -0.063 |
| Pd-N | 0.096 | 0.455 | -0.017 |

**S6:** Bader charge and AIM analysis data for formation pathway to **6** for N- coordination first for key atoms and bonds.

**(1) Ligand charges**

| Atom | Charge /au |
| --- | --- |
| S | -0.013 |
| N | -1.137 |
| C | -0.019 |
| H | 0.048 |

**(2) Ligand AIM data**

| Bond | *ρ*(**r**) | ∇^2^*ρ*(**r**) | *H*(**r**) |
| --- | --- | --- | --- |
| C-H | 0.289 | -1.085 | -0.309 |

**(3) Int 1 charges**

| Atom | Charge /au |
| --- | --- |
| Pd | 0.709 |
| S | 0.007 |
| N | -1.175 |
| C | -0.096 |
| H | 0.054 |

**(4) Int 1 AIM data**

| Bond | *ρ*(**r**) | ∇^2^*ρ*(**r**) | *H*(**r**) |
| --- | --- | --- | --- |
| Pd-N | 0.096 | 0.459 | -0.017 |
| Pd-C | 0.044 | 0.124 | -0.009 |
| Pd-Cl (cleaving) | 0.093 | 0.263 | -0.023 |
| C-H | 0.283 | -1.035 | -0.299 |

**(5) TS 1-2 charges**

| Atom | Charge /au |
| --- | --- |
| Pd | 0.706 |
| S | -0.002 |
| N | -1.149 |
| C | -0.252 |
| H | 0.318 |
| Cl | -0.419 |

**(6) TS 1-2 AIM data**

| Bond | *ρ*(**r**) | ∇^2^*ρ*(**r**) | *H*(**r**) |
| --- | --- | --- | --- |
| Pd-N | 0.105 | 0.467 | -0.023 |
| Pd-C | 0.110 | 0.217 | -0.038 |
| Pd-Cl (cleaving) | 0.014 | 0.048 | 0.002 |
| C-H | 0.097 | -0.050 | -0.046 |

**(7) Int 2 charges**

| Atom | Charge /au |
| --- | --- |
| Pd | 0.661 |
| S | -0.021 |
| N | -1.149 |
| C | -0.145 |
| H | 0.347 |
| Cl | -0.369 |

**(8) Int 2 AIM data**

| Bond | *ρ*(**r**) | ∇^2^*ρ*(**r**) | *H*(**r**) |
| --- | --- | --- | --- |
| Pd-N | 0.107 | 0.478 | -0.023 |
| Pd-C | 0.125 | 0.224 | -0.046 |
| Pd-Cl (cleaving) | 0.062 | 0.229 | -0.010 |
| S-H | 0.072 | -0.002 | -0.027 |

**(9) TS 2-3 charges**

| Atom | Charge /au |
| --- | --- |
| Pd | 0.658 |
| S | -0.042 |
| N | -1.145 |
| C | -0.139 |
| H | 0.316 |
| Cl | -0.241 |

**(10) TS 2-3 AIM data**

| Bond | *ρ*(**r**) | ∇^2^*ρ*(**r**) | *H*(**r**) |
| --- | --- | --- | --- |
| Pd-N | 0.109 | 0.481 | -0.024 |
| Pd-C | 0.133 | 0.212 | -0.052 |
| Pd-S | 0.009 | 0.028 | 0.001 |
| Pd-Cl | 0.046 | 0.163 | -0.007 |
| S-H | 0.016 | 0.042 | 0.001 |

**(11) Int 3 charges**

| Atom | Charge /au |
| --- | --- |
| Pd | 0.575 |
| S | 0.116 |
| N | -1.153 |
| C | -0.122 |
| H | 0.340 |
| Cl | -0.403 |

**(12) Int 3 AIM data**

| Bond | *ρ*(**r**) | ∇^2^*ρ*(**r**) | *H*(**r**) |
| --- | --- | --- | --- |
| Pd-N | 0.097 | 0.455 | -0.018 |
| Pd-C | 0.149 | 0.229 | -0.064 |
| Pd-S | 0.090 | 0.251 | -0.025 |

**(13) Product charges**

| Atom | Charge /au |
| --- | --- |
| Pd | 0.578 |
| S | 0.105 |
| N | -1.152 |
| C | -0.126 |

**(14) Product AIM data**

| Bond | *ρ*(**r**) | ∇^2^*ρ*(**r**) | *H*(**r**) |
| --- | --- | --- | --- |
| Pd-N | 0.097 | 0.457 | -0.018 |
| Pd-C | 0.148 | 0.240 | -0.063 |
| Pd-S | 0.091 | 0.257 | -0.025 |

**S7:** Bader charge and AIM analysis data for PdCl_2_.

**(1) Charges**

| Atom | Charge /au |
| --- | --- |
| Pd | 0.683 |
| Cl | -0.341 |

**(2) AIM data**

| Bond | *ρ*(**r**) | ∇^2^*ρ*(**r**) | *H*(**r**) |
| --- | --- | --- | --- |
| Pd-Cl | 0.110 | 0.276 | -0.033 |

**S8:** Molecular orbitals involving significant Pd-donor atom interactions in **Int 1** for **6**.

N-coordinates first molecular orbitals for **Int 1**.

| Molecular orbitals | | Molecular orbital number | Orbital energy / au |
| --- | --- | --- | --- |
| 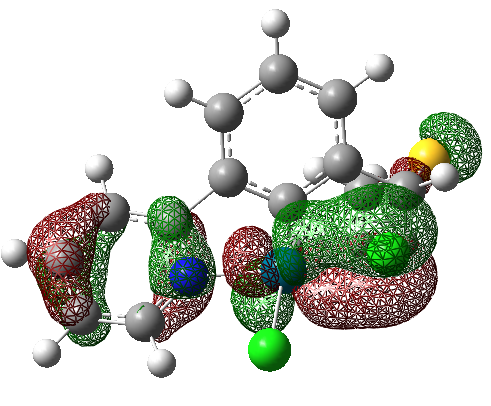 |  | HOMO-8  MO 75 | -0.39091 |
| 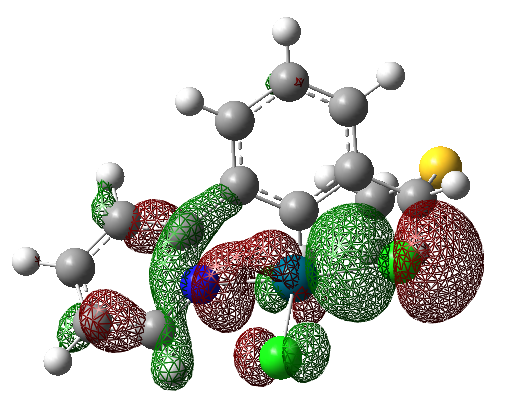 |  | HOMO-9  MO 74 | -0.39125 |
| 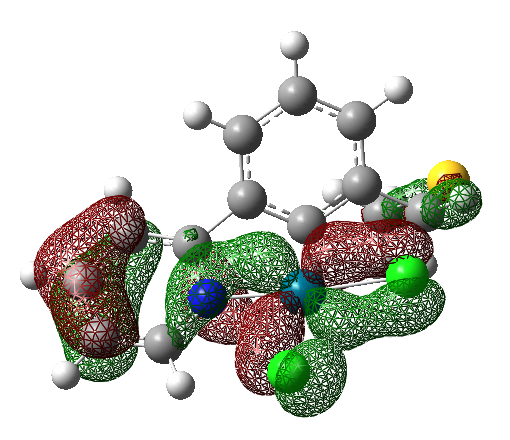 |  | HOMO-13  MO 70 | -0.43208 |
| 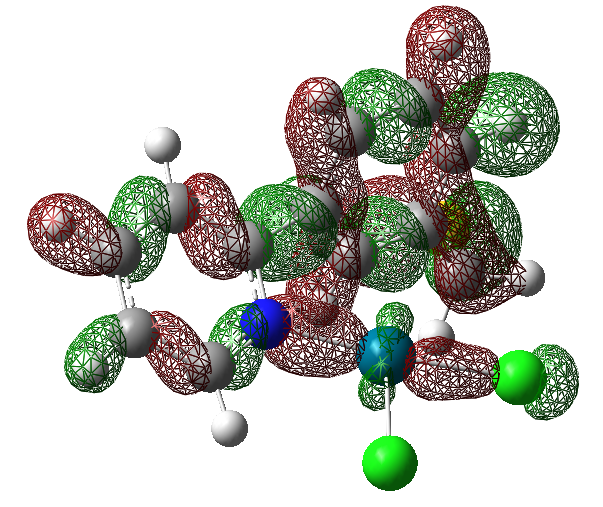 |  | HOMO-17  MO 66 | -0.46496 |
| 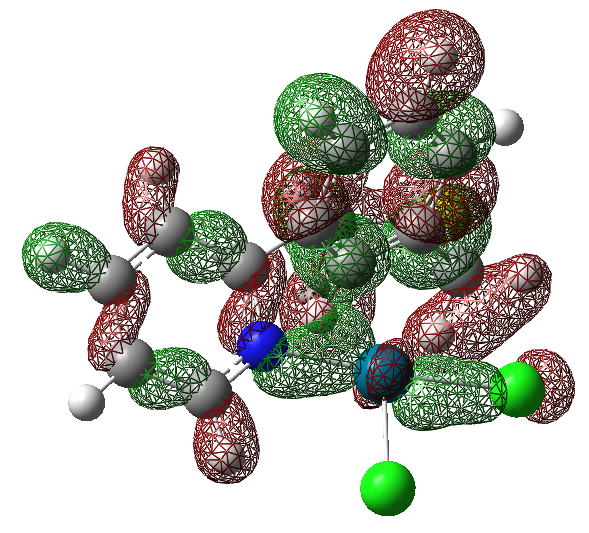 |  | HOMO-18  MO 65 | -0.46814 |
| 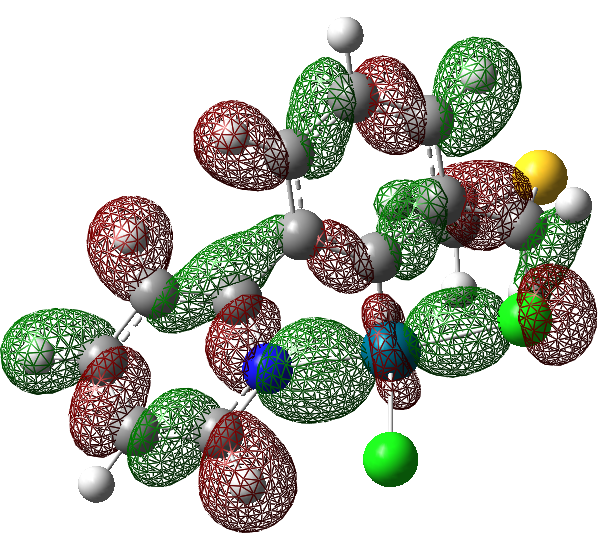 |  | HOMO-19  MO 64 | -0.47870 |
| 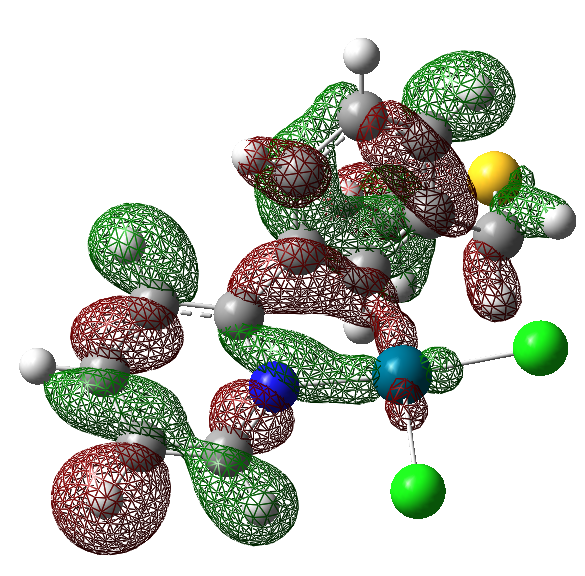 |  | HOMO-20  MO 63 | -0.50381 |
| 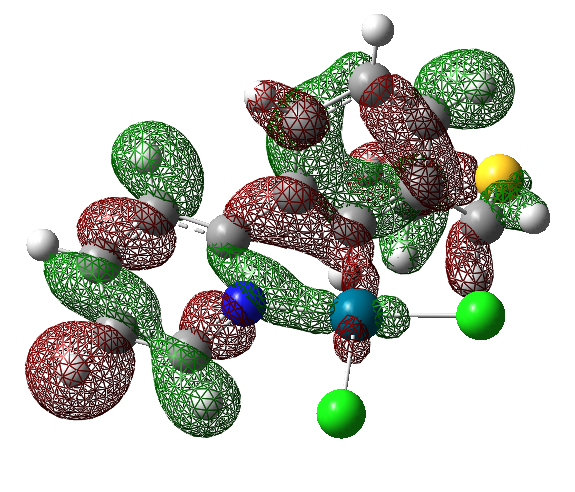 |  | HOMO-27  MO 56 | -0.55597 |
| 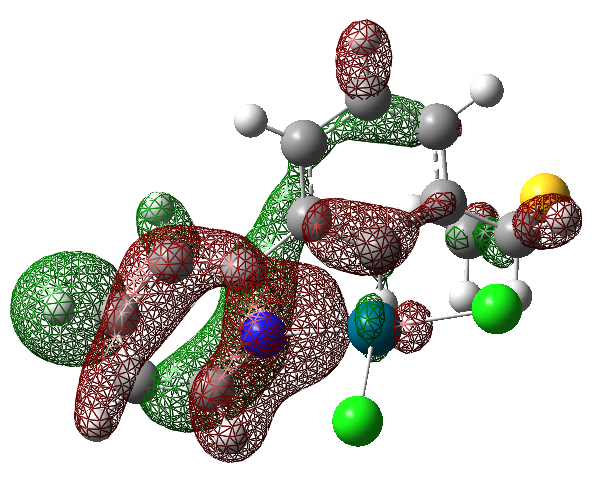 |  | HOMO-28  MO 55 | -0.56289 |

S-coordinates first molecular orbitals for **Int 1**.

| Molecular orbitals | | Molecular orbital number | Orbital energy / au |
| --- | --- | --- | --- |
| 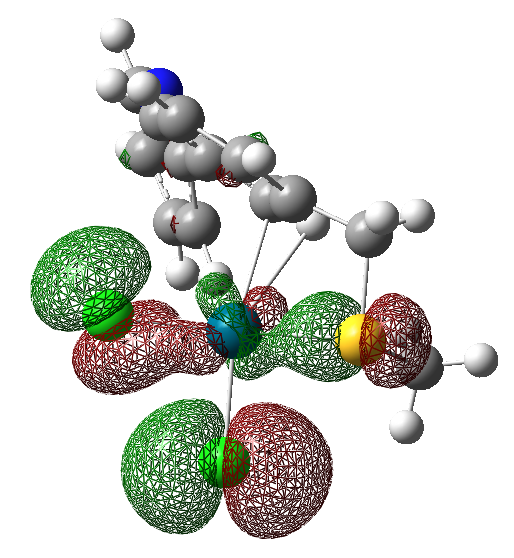 |  | HOMO-3  MO 80 | -0.33188 |
| 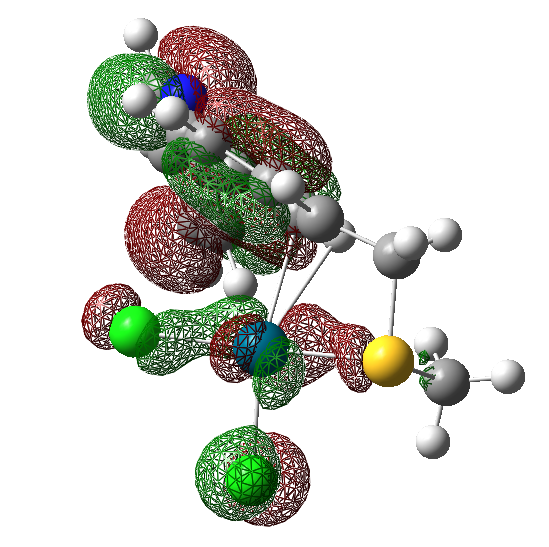 |  | HOMO-8  MO 75 | -0.37268 |
| 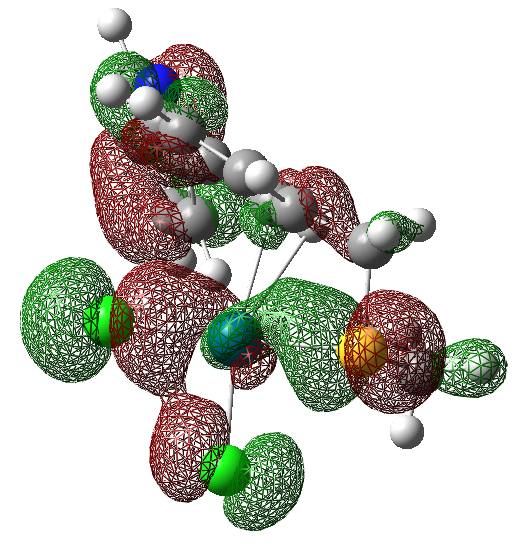 |  | HOMO-9  MO 74 | -0.37839 |
| 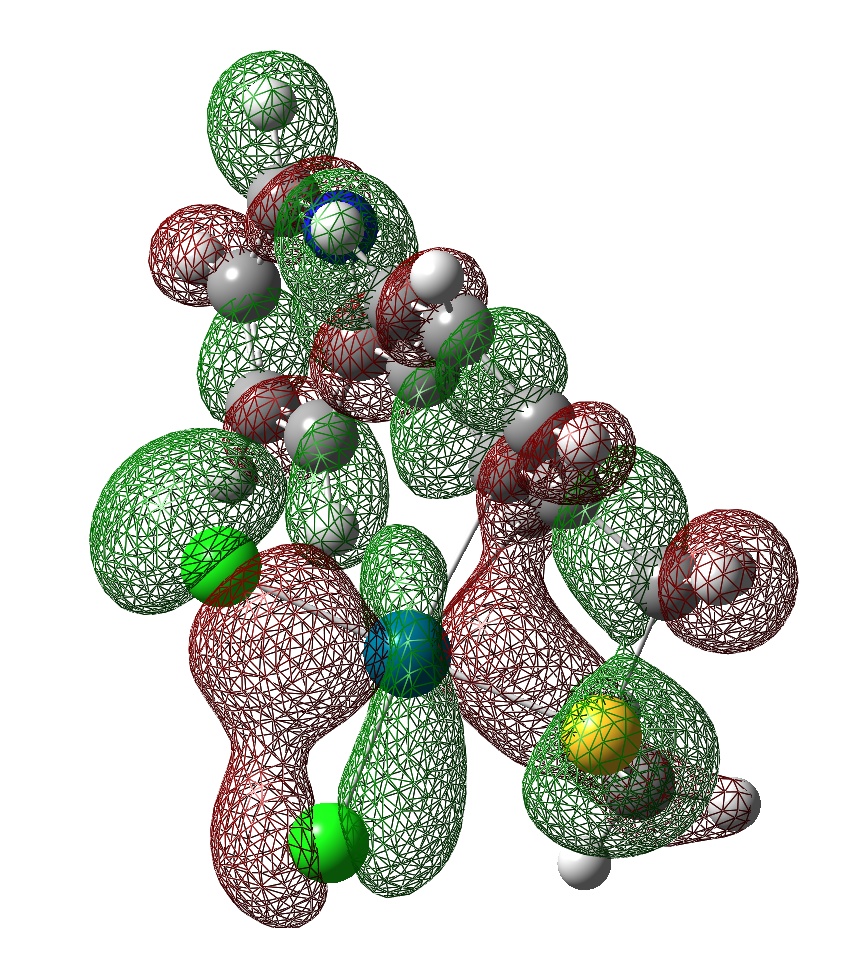 |  | HOMO-15  MO 68 | -0.44720 |
| 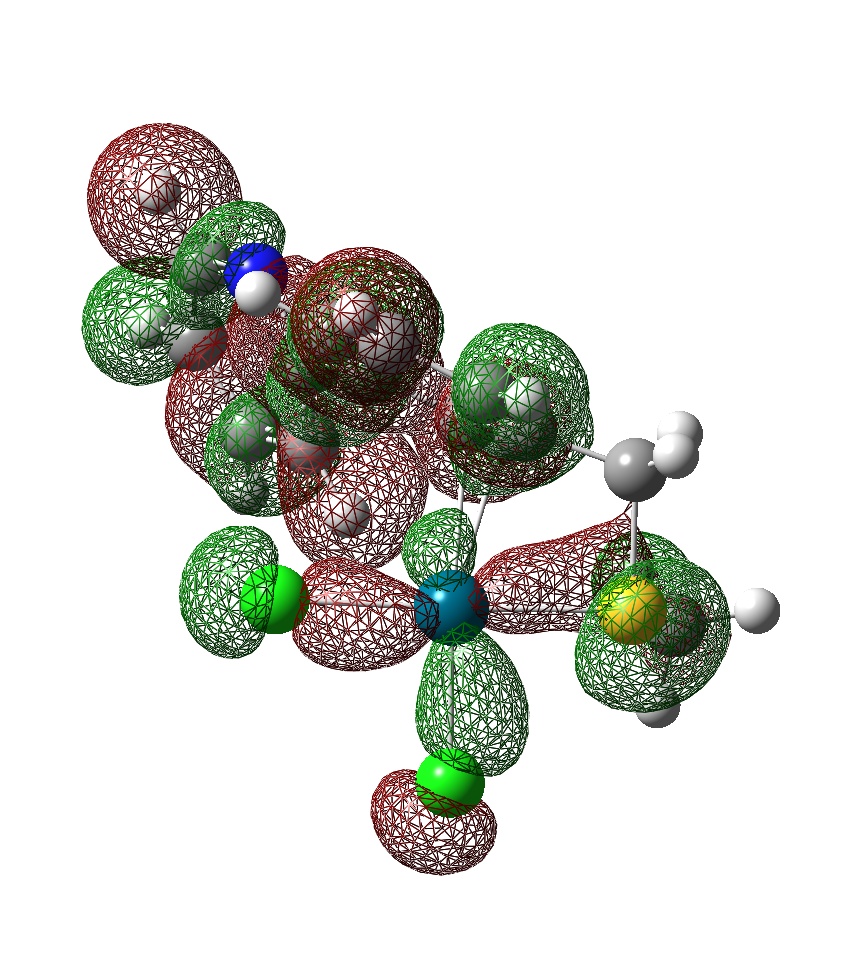 |  | HOMO-16  MO 67 | -0.45071 |

**S9:** Cartesian coordinates, energies, zero-point energy correction, thermal correction to Gibbs free energy, single point and solvent corrected energies for pathway **III** with S-coordination first.

**(1) Ligand**

C 0.15679100 -0.03682400 0.01209500

C 0.01714000 2.73468000 -0.06258300

C -1.02442200 0.59982700 0.39047000

C -1.08868600 1.99619200 0.34335500

C 1.19486100 2.08540300 -0.43062000

C 1.27222200 0.69381500 -0.40301800

C -2.22286700 -0.20766200 0.81387400

C -4.55645700 -1.55860700 0.19588100

C 2.52846500 -0.02865200 -0.83773900

C 3.96756100 -1.91483500 -0.47795500

C 3.35635800 -0.51537800 1.36962200

H -2.00818500 2.50136000 0.62663900

H -0.03538200 3.81877800 -0.08806500

H 2.06058400 2.66629100 -0.73855300

H -1.90741800 -1.13660900 1.29900900

H -2.83884200 0.35957500 1.51895400

H -5.08511000 -0.93176200 0.91922400

H -4.15371000 -2.44206000 0.69868300

H -5.26584000 -1.88414100 -0.56761400

H 3.33825300 0.71114200 -0.99905300

H 2.34274800 -0.50675200 -1.80718900

H 4.89686000 -1.35863100 -0.70961500

H 3.60311300 -2.37371500 -1.40181600

H 4.21863400 -2.71519400 0.22399900

H 4.24959400 0.13220200 1.27212300

H 3.59477200 -1.32640700 2.06352200

H 2.54857600 0.07961200 1.80314400

H 0.22764700 -1.12106200 0.04893100

N 2.93542000 -1.07021300 0.09447700

S -3.24625200 -0.62930500 -0.64399700

Optimization using ωB97XD/6-31++G(d,p)[SDD]

- Energy = -882.2038541 au
- Zero-point energy correction = 0.261636 au
- Thermal correction to Gibbs free energy = 0.218736 au

Single point energy using ωB97XD/6-311++G(2df,2p)[SDD]

- Energy = -882.3484668 au

Single point PCM acetonitrile solvent corrected energy using ωB97XD/6-311++G(2df,2p)[SDD]

- Energy = -882.3553872 au

**(2) Int 1**

C 0.95424700 1.02928500 0.30474700

C 1.39870700 2.55988400 -1.97096800

C 0.12882400 2.14149500 0.03099800

C 0.34748600 2.89672000 -1.11692200

C 2.23407500 1.48637100 -1.68361800

C 2.03314100 0.71635500 -0.53679700

C -0.99921300 2.45242200 0.98086400

C -2.56515000 0.54178700 2.40795500

C 2.93748300 -0.44918700 -0.22115900

C 3.19749100 -2.24071400 1.37388800

C 3.75396800 0.02058000 1.99216400

H -0.29459600 3.74232200 -1.34463400

H 1.56829900 3.14707400 -2.86803400

H 3.05560100 1.24582600 -2.35218000

H -0.66672500 2.41010900 2.02125300

H -1.44326100 3.43092100 0.79042300

H -3.10469900 1.29079400 2.99030400

H -1.61811500 0.28194400 2.88336200

H -3.17186200 -0.35794400 2.29243900

H 3.96283300 -0.22031700 -0.56795200

H 2.57110100 -1.29006100 -0.82105300

H 4.22669600 -2.50756000 1.06867300

H 2.49068300 -2.84380400 0.79837200

H 3.08187700 -2.49169100 2.43254800

H 4.82747100 -0.10088200 1.75189700

H 3.61186400 -0.21423400 3.05138900

H 3.49079400 1.07233600 1.83857200

H 0.89302300 0.50137000 1.25680500

N 2.90375900 -0.83015900 1.17860900

Pd -0.95657600 -0.46640500 -0.31383800

Cl -2.81485000 -1.79623000 -0.31258700

Cl 0.24063300 -2.05069600 -1.45472200

S -2.28559100 1.16483000 0.72758800

Optimization using ωB97XD/6-31++G(d,p)[SDD]

- Energy = -1930.5499666 au
- Zero-point energy correction = 0.265783 au
- Thermal correction to Gibbs free energy = 0.215062 au

Single point energy using ωB97XD/6-311++G(2df,2p)[SDD]

- Energy = -1930.7740038 au

Single point PCM acetonitrile solvent corrected energy using ωB97XD/6-311++G(2df,2p)[SDD]

- Energy = -1930.8026598 au

**(3) TS 1-2**

C 1.27948400 0.60342000 0.10244100

C 3.34042600 2.56214600 0.18891500

C 1.03271100 1.91139100 0.57919900

C 2.05607500 2.86097500 0.62978200

C 3.58636400 1.31079100 -0.35709300

C 2.56690700 0.36359100 -0.40498300

C -0.37015800 2.37828500 0.86180400

C -2.98995900 2.38094800 -0.18148600

C 2.78766400 -1.00159100 -1.01788700

C 1.22108600 -2.88059400 -1.22564900

C 2.43561800 -2.48989900 0.85461300

H 1.83606800 3.85718500 1.00338300

H 4.12723800 3.30707400 0.24611700

H 4.57045200 1.06129800 -0.74567400

H -0.83320700 1.86986000 1.71327400

H -0.42058600 3.45968400 1.01160800

H -3.04486200 3.46946700 -0.10847300

H -3.26768500 1.90076600 0.75709700

H -3.65328400 2.02051300 -0.96726700

H 3.82547800 -1.34259700 -0.91397200

H 2.55257100 -0.96917500 -2.08692400

H 1.97125700 -3.56567600 -1.64428400

H 0.70569700 -2.36466900 -2.04044200

H 0.48013600 -3.44106000 -0.65485000

H 3.26974900 -3.15263000 0.58446800

H 1.66680500 -3.04592900 1.38863900

H 2.80316700 -1.69394400 1.50768500

H 0.98866800 -0.75240100 0.04654000

N 1.84367800 -1.88944800 -0.34636000

Pd -0.94950600 -0.31733900 -0.02210800

Cl -3.13227900 -0.84076400 -0.48391500

Cl -0.84759100 -2.19814700 1.34306200

S -1.30160400 1.92941800 -0.65243100

Optimization using ωB97XD/6-31++G(d,p)[SDD]

- Energy = -1930.5037645 au
- Zero-point energy correction = 0.262993 au
- Thermal correction to Gibbs free energy = 0.215942 au

Single point energy using ωB97XD/6-311++G(2df,2p)[SDD]

- Energy = -1930.7276588 au

Single point PCM acetonitrile solvent corrected energy using ωB97XD/6-311++G(2df,2p)[SDD]

- Energy = -1930.7537483 au

**(4) Int 2**

C 0.40683900 1.03592200 -0.03329300

C 1.88568200 3.47023300 0.06373400

C -0.18283900 2.26050600 0.36194300

C 0.54075400 3.44941100 0.42309700

C 2.46938100 2.30148700 -0.40396800

C 1.74749200 1.10008200 -0.46114800

C -1.66585400 2.25827400 0.62717400

C -3.95389800 0.67250800 0.16292000

C 2.43289300 -0.08844600 -1.08303600

C 3.63509000 -2.20797100 -0.75208900

C 3.97758800 -0.38669700 0.86173500

H 0.04815200 4.36911200 0.72883200

H 2.45318000 4.39431200 0.10223700

H 3.49948000 2.32391600 -0.75549700

H -1.90595700 1.87239000 1.62287000

H -2.12005200 3.24482800 0.50133300

H -4.59898300 1.55355600 0.13142000

H -3.80345500 0.31970700 1.18371800

H -4.37920000 -0.14233000 -0.42287000

H 3.24026600 0.22793500 -1.75207200

H 1.71713700 -0.68887100 -1.65001600

H 4.46616300 -1.88988200 -1.38641900

H 2.86272700 -2.69186400 -1.35109500

H 3.98601500 -2.90847800 0.00641400

H 4.82874300 0.02458900 0.31272200

H 4.31965000 -1.13657500 1.57594200

H 3.45578800 0.41009500 1.39021700

H 2.20132300 -1.40418800 0.47332400

N 3.04073100 -1.03027300 -0.08198000

Pd -0.85335600 -0.57426400 -0.05742200

Cl -2.49942300 -2.28529900 -0.26103900

Cl 0.60488500 -2.11721400 1.05469600

S -2.36583300 1.08754100 -0.59939800

Optimization using ωB97XD/6-31++G(d,p)[SDD]

- Energy = -1930.5689923 au
- Zero-point energy correction = 0.268865 au
- Thermal correction to Gibbs free energy = 0.221084 au

Single point energy using ωB97XD/6-311++G(2df,2p)[SDD]

- Energy = -1930.7913445 au

Single point PCM acetonitrile solvent corrected energy using ωB97XD/6-311++G(2df,2p)[SDD]

- Energy = -1930.8294357 au

**(5) TS 2-3**

C 0.63968300 1.04924700 -0.35081100

C 2.70182900 2.91814800 -0.22178700

C 0.37547700 2.35094500 0.08613100

C 1.40984400 3.28943600 0.13921300

C 2.96687300 1.61128900 -0.63566400

C 1.94021300 0.67164600 -0.70736800

C -1.02828900 2.65709400 0.52389800

C -3.28882900 0.98876600 0.99090600

C 2.16463100 -0.76310900 -1.12544300

C 1.61495000 -3.05339900 -0.48465500

C 3.02084500 -1.69168200 0.92380900

H 1.20943100 4.30653500 0.46596700

H 3.50499100 3.64662200 -0.17911700

H 3.98006700 1.32356600 -0.90705500

H -1.14808800 2.48842000 1.59898100

H -1.35190400 3.67348300 0.28483100

H -3.92391900 1.84287800 1.23549200

H -2.73477900 0.64031200 1.86358100

H -3.88850800 0.16812000 0.59450400

H 3.18966000 -0.90894900 -1.50402600

H 1.48523300 -1.01971100 -1.94843100

H 2.48323100 -3.48382300 -1.00829900

H 0.74866300 -3.04964600 -1.15103700

H 1.36879100 -3.68601600 0.37175600

H 3.94260600 -2.03310500 0.42620500

H 2.80838100 -2.35529800 1.76509200

H 3.17695500 -0.68224700 1.31241400

H 0.74470100 -1.29954200 1.36227100

N 1.88900700 -1.69364000 -0.00935600

Pd -0.76197900 -0.32781900 -0.51000700

Cl -2.17729100 -2.19319200 -0.81184100

Cl 0.14221500 -1.11278700 2.54337800

S -2.14216100 1.46218000 -0.33267500

Optimization using ωB97XD/6-31++G(d,p)[SDD]

- Energy = -1930.5311641 au
- Zero-point energy correction = 0.262527 au
- Thermal correction to Gibbs free energy = 0.213347 au

Single point energy using ωB97XD/6-311++G(2df,2p)[SDD]

- Energy = -1930.7544406 au

Single point PCM acetonitrile solvent corrected energy using ωB97XD/6-311++G(2df,2p)[SDD]

- Energy = -1930.7790863 au

**(6) Int 3**

C -1.30687800 -0.08578100 -0.33492100

C -4.05005300 -0.40643200 -0.03678000

C -1.85189100 -1.37338200 -0.31002600

C -3.23403800 -1.52861000 -0.17372400

C -3.50256700 0.87858000 -0.04856900

C -2.12860800 1.03891900 -0.21010900

C -0.89846700 -2.53801700 -0.38172100

C 1.94605400 -2.85669000 -0.26616200

C -1.42590700 2.36421500 -0.32909600

C 0.82388600 3.28210400 -0.52919400

C 0.09928800 2.34869100 1.57001300

H -3.67642400 -2.52137100 -0.16055300

H -5.12099900 -0.53420600 0.08421400

H -4.15097600 1.74439500 0.06068200

H -0.64000100 -2.88711200 0.62377100

H -1.28852800 -3.38028800 -0.95890200

H 1.95893200 -3.89374000 -0.60586800

H 1.77705700 -2.79719300 0.80992900

H 2.88728500 -2.35925100 -0.50727200

H -1.91288400 3.16495400 0.24550400

H -1.41656700 2.67269000 -1.37996200

H 0.43864200 4.27531400 -0.25849400

H 0.80100500 3.16211100 -1.61372000

H 1.85576900 3.17276700 -0.19441800

H -0.18227400 3.36350200 1.88514600

H 1.12680300 2.14483900 1.87713700

H -0.56986900 1.62728900 2.04236800

H 0.73607400 -0.56645200 1.65779200

N 0.00098000 2.22951100 0.09886600

Pd 0.62344500 0.24703900 -0.45745700

Cl 2.99110500 0.62120600 -0.37439300

Cl 0.61071800 -1.14788300 2.82741000

S 0.66473100 -1.94666700 -1.16599600

Optimization using ωB97XD/6-31++G(d,p)[SDD]

- Energy = -1930.5730576 au
- Zero-point energy correction = 0.263767 au
- Thermal correction to Gibbs free energy = 0.215907 au

Single point energy using ωB97XD/6-311++G(2df,2p)[SDD]

- Energy = -1930.7948443 au

Single point PCM acetonitrile solvent corrected energy using ωB97XD/6-311++G(2df,2p)[SDD]

- Energy = -1930.813735 au

**(7) Product**

C 1.24905700 0.07748500 -0.10357500

C 4.02763400 0.05582200 0.06947900

C 1.94380200 1.28174900 0.05198900

C 3.34009900 1.26719900 0.12323700

C 3.33420600 -1.14918500 -0.06861100

C 1.94506200 -1.13555600 -0.16731400

C 1.13444400 2.54577900 0.18538800

C -1.61870700 3.06467400 0.70431500

C 1.09059300 -2.35203400 -0.40416400

C -1.25222900 -3.00532800 -0.55648700

C -0.32965600 -2.37416000 1.57594600

H 3.89246300 2.19735900 0.23231700

H 5.11092400 0.04910300 0.13888600

H 3.88109300 -2.08822500 -0.10657700

H 0.99840300 2.81068600 1.23909000

H 1.58299800 3.39910900 -0.33035200

H -1.51054000 4.14721800 0.61517800

H -1.37979000 2.72575800 1.71327300

H -2.63754200 2.75755600 0.46121300

H 1.50999700 -3.26708500 0.03918700

H 0.99569000 -2.52102200 -1.48191500

H -0.97409600 -4.05798400 -0.40208500

H -1.26499600 -2.78061600 -1.62430100

H -2.24863800 -2.81648200 -0.15615600

H -0.13489300 -3.43767300 1.77805800

H -1.31649500 -2.10411500 1.95600300

H 0.42726200 -1.76719500 2.07567700

N -0.28831200 -2.11804300 0.12069500

Pd -0.70648200 -0.02566100 -0.15664600

Cl -3.10473600 -0.16663000 -0.00583600

S -0.55130100 2.24589900 -0.50929100

Optimization using ωB97XD/6-31++G(d,p)[SDD]

- Energy = -1469.77668 au
- Zero-point energy correction = 0.254979 au
- Thermal correction to Gibbs free energy = 0.211050 au

Single point energy using ωB97XD/6-311++G(2df,2p)[SDD]

- Energy = -1469.9635673 au

Single point PCM acetonitrile solvent corrected energy using ωB97XD/6-311++G(2df,2p)[SDD]

- Energy = -1469.9833601 au

**S10:** Cartesian coordinates, energies, zero-point energy correction, thermal correction to Gibbs free energy, single point and solvent corrected energies for pathway **III** with N-coordination first.

1. **Ligand**

C 0.15682000 -0.03649500 0.01244300

C 0.01692900 2.73499300 -0.06279000

C 1.27220300 0.69415000 -0.40272000

C 1.19470600 2.08574800 -0.43063300

C -1.08885500 1.99648400 0.34326000

C -1.02448200 0.60014200 0.39062600

C 2.52856600 -0.02815500 -0.83736400

C 3.96704100 -1.91496500 -0.47841800

C -2.22289400 -0.20735700 0.81403600

C -4.55631200 -1.55858500 0.19570000

H 2.06041200 2.66663500 -0.73861100

H -0.03568700 3.81908000 -0.08849700

H -2.00841800 2.50162200 0.62638500

H 2.34319800 -0.50562100 -1.80719500

H 3.33845900 0.71169100 -0.99793400

H 4.89655200 -1.35896200 -0.70970600

H 3.60252200 -2.37325800 -1.40253600

H 4.21777000 -2.71573600 0.22318200

H -2.83933000 0.36016500 1.51846500

H -1.90752800 -1.13599600 1.29980200

H -5.08467800 -0.93143800 0.91898500

H -4.15406600 -2.44224400 0.69852600

H -5.26580800 -1.88371500 -0.56785800

H 0.22776700 -1.12071900 0.04951200

S -3.24554000 -0.63001000 -0.64412600

C 3.35614100 -0.51621700 1.36979300

H 3.59452600 -1.32764200 2.06323400

H 4.24943400 0.13134000 1.27263300

H 2.54843100 0.07861100 1.80366300

N 2.93512800 -1.07030300 0.09433700

Optimization using ωB97XD/6-31++G(d,p)[SDD]

- Energy = -882.2038539 au
- Zero-point energy correction = 0.261636 au
- Thermal correction to Gibbs free energy = 0.218741 au

Single point energy using ωB97XD/6-311++G(2df,2p)[SDD]

- Energy = -882.3484669 au

Single point PCM acetonitrile solvent corrected energy using ωB97XD/6-311++G(2df,2p)[SDD]

- Energy = -882.3553874 au

**(2) Int 1**

C 1.00191100 0.98085200 0.70095200

C 1.05047100 2.89136400 -1.31767900

C -0.09823600 1.84467200 0.52378800

C -0.06460700 2.80645300 -0.49922300

C 2.13425100 2.02685500 -1.13702700

C 2.12797100 1.06585800 -0.13400900

C -1.25530300 1.73108100 1.49622400

C -2.46758700 -0.29121500 2.21237900

C 3.29871400 0.11649200 -0.00963900

C 2.37244000 -2.32411800 1.02074800

H -0.90277400 3.48385800 -0.63347300

H 1.08288900 3.63362500 -2.10843200

H 2.99218800 2.10177600 -1.79923800

H -0.87434200 1.54554900 2.50344000

H -1.85484500 2.64684400 1.52702900

H -3.07743100 0.26955100 2.93334800

H -1.56294000 -0.65709800 2.70131000

H -3.02628800 -1.14600400 1.82997700

H 4.23387700 0.68200400 -0.05814400

H 3.27701900 -0.55443500 -0.87649300

H 2.73808900 -2.77753900 0.09745000

H 1.32426000 -2.05118400 0.88208900

H 2.45448400 -3.04909900 1.83333700

H 1.05558800 0.33147700 1.57268700

S 3.40240000 -0.90268200 1.49029100

Pd -0.79688100 -0.33900300 -0.33302200

Cl -2.29757100 -2.01727700 -0.74490300

Cl 0.59680400 -1.10996400 -1.98256600

C -3.30780400 1.03311200 0.36640900

H -3.83645800 0.16528000 -0.02814900

H -3.95462800 1.59464600 1.05417500

H -3.01802100 1.67456100 -0.46826400

N -2.09611800 0.57398300 1.07730300

Optimization using ωB97XD/6-31++G(d,p)[SDD]

- Energy = -1930.5625161 au
- Zero-point energy correction = 0.267634 au
- Thermal correction to Gibbs free energy = 0.218503 au

Single point energy using ωB97XD/6-311++G(2df,2p)[SDD]

- Energy = -1930.7830549 au

Single point PCM acetonitrile solvent corrected energy using ωB97XD/6-311++G(2df,2p)[SDD]

- Energy = -1930.8114444 au

**(3) TS 1-2**

C -0.37119900 0.92478000 -0.19391800

C -1.49166700 3.50400000 -0.10162000

C 0.37746300 2.06403200 -0.57176600

C -0.17347200 3.33658200 -0.52881500

C -2.21141300 2.40774400 0.34463100

C -1.66249000 1.11462400 0.33019500

C 1.83801200 1.84794500 -0.84489300

C 3.74808800 0.47964700 -0.24192700

C -2.49641400 -0.00105800 0.90980700

C -4.48293400 -1.89103000 0.61955700

H 0.42656600 4.20115400 -0.79982000

H -1.93668300 4.49346600 -0.07613300

H -3.21886600 2.54217000 0.72951700

H 1.99894900 1.41433600 -1.83716000

H 2.41025400 2.78434700 -0.78327700

H 4.38258600 1.37573100 -0.27662400

H 3.72696500 -0.00161600 -1.21996900

H 4.13859700 -0.23592300 0.47948200

H -2.85203700 0.29691500 1.90197600

H -1.91768800 -0.91887500 1.02374300

H -4.72584500 -1.74203700 1.67499200

H -3.71176200 -2.65950900 0.51903700

H -5.38196500 -2.22090100 0.09523200

H -0.60288000 -0.45683500 -0.95534600

S -3.94451000 -0.34475300 -0.15443200

Pd 0.96459700 -0.68526600 -0.01275900

Cl 2.47883700 -2.39495000 0.46960000

Cl -0.75924000 -1.96069500 -1.14041800

C 2.40684000 1.44327500 1.51171200

H 2.73226900 0.66825500 2.20682900

H 3.10964300 2.28698800 1.54470700

H 1.41235800 1.78815900 1.79488100

N 2.37602700 0.86930600 0.14884200

Optimization using ωB97XD/6-31++G(d,p)[SDD]

- Energy = -1930.5051015 au
- Zero-point energy correction = 0.261152 au
- Thermal correction to Gibbs free energy = 0.213184 au

Single point energy using ωB97XD/6-311++G(2df,2p)[SDD]

- Energy = -1930.7284906 au

Single point PCM acetonitrile solvent corrected energy using ωB97XD/6-311++G(2df,2p)[SDD]

- Energy = -1930.7513724 au

**(4) Int 2**

C -0.25627500 1.08829500 -0.04912800

C -1.30603300 3.72242100 -0.17265300

C 0.53265100 2.19217900 -0.43521900

C 0.02658000 3.48592100 -0.50174900

C -2.08793800 2.66370700 0.26608000

C -1.57639900 1.35915700 0.35163300

C 1.97121900 1.89133000 -0.72977200

C 3.69407600 0.22670400 -0.39605500

C -2.49174500 0.30334600 0.91684200

C -4.43095000 -1.66768800 0.53613900

H 0.67121100 4.30949400 -0.79937700

H -1.72017200 4.72390200 -0.22830700

H -3.11839300 2.84562200 0.56348100

H 2.09969300 1.58500700 -1.77299800

H 2.63043400 2.75284300 -0.54747500

H 4.42964500 1.04274900 -0.40882500

H 3.55509500 -0.16825600 -1.40268800

H 4.03724600 -0.58627500 0.24097100

H -3.15612800 0.73005100 1.67309700

H -1.92725400 -0.51524200 1.37200200

H -5.02290500 -1.20525000 1.32874900

H -3.72733100 -2.38543300 0.96655600

H -5.09965400 -2.18759600 -0.15176200

H -2.03299500 -1.49248900 -0.79327800

S -3.54946400 -0.39563800 -0.40986200

Pd 0.82068000 -0.63206200 -0.01987700

Cl 2.22790400 -2.55439600 0.22629700

Cl -0.89151100 -2.24562300 -0.62869500

C 2.58569700 1.16224700 1.52351200

H 2.82990700 0.28191400 2.11979400

H 3.40313200 1.89362900 1.59168600

H 1.66510100 1.61053000 1.89729500

N 2.40564400 0.74349700 0.11517100

Optimization using ωB97XD/6-31++G(d,p)[SDD]

- Energy = -1930.5368886 au
- Zero-point energy correction = 0.264455 au
- Thermal correction to Gibbs free energy = 0.217018 au

Single point energy using ωB97XD/6-311++G(2df,2p)[SDD]

- Energy = -1930.7588212 au

Single point PCM acetonitrile solvent corrected energy using ωB97XD/6-311++G(2df,2p)[SDD]

- Energy = -1930.7810462 au

**(5) TS 2-3**

C 0.08225500 1.17555900 -0.14903600

C 0.82362200 3.86018800 0.00850100

C -0.80812200 2.12713600 0.36337900

C -0.44230000 3.46824700 0.44345300

C 1.70302400 2.91852500 -0.52016600

C 1.34039400 1.56966200 -0.61468300

C -2.12507100 1.57006700 0.81689000

C -3.38956700 -0.52123600 0.70063900

C 2.27991400 0.55816200 -1.22143900

C 3.30297400 -1.97517200 -0.57102200

H -1.13671600 4.20586400 0.83777000

H 1.11860000 4.90285700 0.06779100

H 2.68005100 3.23401000 -0.87916300

H -2.05478200 1.24928900 1.86137100

H -2.95566800 2.28547700 0.73519100

H -4.32930900 0.02262400 0.87060600

H -2.96859600 -0.83933300 1.65534900

H -3.57420500 -1.40712100 0.09244200

H 2.93366400 1.02859300 -1.95999900

H 1.71933600 -0.23259500 -1.73303500

H 3.74983500 -2.04334300 -1.56463600

H 2.27867200 -2.36112200 -0.58930600

H 3.89234700 -2.57556600 0.12450200

H 1.92015500 -0.67047800 1.54382500

S 3.34372100 -0.26376600 0.03710100

Pd -0.58981200 -0.66817700 -0.24485000

Cl -1.07096100 -2.94148800 -0.71936400

Cl 0.93955800 -1.15704400 2.30189400

C -2.96128000 0.73154200 -1.32097200

H -3.07785000 -0.16672100 -1.92850600

H -3.93655300 1.22336800 -1.19739800

H -2.26855000 1.41483000 -1.81324000

N -2.42283400 0.35210100 0.00423200

Optimization using ωB97XD/6-31++G(d,p)[SDD]

- Energy = -1930.5221989 au
- Zero-point energy correction = 0.262746 au
- Thermal correction to Gibbs free energy = 0.213835 au

Single point energy using ωB97XD/6-311++G(2df,2p)[SDD]

- Energy = -1930.7426346 au

Single point PCM acetonitrile solvent corrected energy using ωB97XD/6-311++G(2df,2p)[SDD]

- Energy = -1930.7703005 au

**(6) Int 3**

C -1.27688900 0.13892800 -0.18504000

C -4.05221400 0.04651200 -0.31254100

C -1.92749700 -1.09209500 -0.32486300

C -3.31720800 -1.13792300 -0.40010400

C -3.40755600 1.27309800 -0.16362200

C -2.01136600 1.32660400 -0.11312100

C -1.03229800 -2.30124400 -0.31831300

C 1.33386100 -2.88810000 -0.28077800

C -1.25144900 2.62531000 -0.03030800

C 1.46446700 3.31194300 -0.57515900

H -3.83183300 -2.08827300 -0.51716200

H -5.13597100 0.01189000 -0.36205400

H -3.99326100 2.18655700 -0.09683900

H -0.92604500 -2.66285900 0.70995500

H -1.42341800 -3.13082700 -0.92406100

H 1.08733900 -3.90493200 -0.61720300

H 1.34216300 -2.85542600 0.80993800

H 2.32089400 -2.59967700 -0.64316800

H -1.70425900 3.34158800 0.66040400

H -1.17288000 3.09713300 -1.01516900

H 1.30956200 4.36371200 -0.32777200

H 1.20967200 3.11068400 -1.61647900

H 2.50301400 3.02471500 -0.40210800

H 0.41651700 -0.59776800 1.96276200

S 0.47320500 2.27829000 0.53466000

Pd 0.68559300 0.09881500 -0.18677700

Cl 3.07363900 0.08097700 -0.40798600

Cl 0.21921800 -1.21491000 3.10008000

C 0.37736100 -1.92638600 -2.27375300

H 1.35181400 -1.55850900 -2.60031500

H 0.21999800 -2.94412600 -2.65825400

H -0.40482000 -1.27027200 -2.66003500

N 0.33558500 -1.92925400 -0.79503400

Optimization using ωB97XD/6-31++G(d,p)[SDD]

- Energy = -1930.5715106 au
- Zero-point energy correction = 0.263585 au
- Thermal correction to Gibbs free energy = 0.214635 au

Single point energy using ωB97XD/6-311++G(2df,2p)[SDD]

- Energy = -1930.7934012 au
- Energy = -1930.7426346 au

Single point PCM acetonitrile solvent corrected energy using ωB97XD/6-311++G(2df,2p)[SDD]

- Energy = -1930.8136794 au

**(7) Product**

C 1.24905600 0.07754900 -0.10370200

C 4.02763200 0.05597400 0.06965100

C 1.94512700 -1.13549200 -0.16733200

C 3.33424000 -1.14906500 -0.06846200

C 3.34006700 1.26731700 0.12326900

C 1.94376200 1.28182800 0.05187200

C 1.09069900 -2.35199200 -0.40422000

C -1.25209400 -3.00539300 -0.55646100

C 1.13437300 2.54585500 0.18513800

C -1.61879800 3.06437100 0.70457600

H 3.88117400 -2.08808600 -0.10629900

H 5.11091100 0.04925500 0.13921000

H 3.89237500 2.19750800 0.23236100

H 0.99580500 -2.52090400 -1.48198400

H 1.51015400 -3.26705000 0.03906200

H -0.97395500 -4.05803700 -0.40197400

H -1.26483400 -2.78077300 -1.62429800

H -2.24850700 -2.81651200 -0.15616100

H 1.58284800 3.39910700 -0.33080000

H 0.99847800 2.81093500 1.23881800

H -1.51083900 4.14694600 0.61553100

H -1.37960500 2.72537700 1.71344200

H -2.63764600 2.75711600 0.46169600

S -0.55145100 2.24588300 -0.50930500

Pd -0.70651000 -0.02569400 -0.15669700

Cl -3.10471500 -0.16667000 -0.00575700

C -0.32949600 -2.37406300 1.57595400

H -1.31632300 -2.10400600 1.95604300

H -0.13471400 -3.43755500 1.77814000

H 0.42743800 -1.76707100 2.07562900

N -0.28819100 -2.11805600 0.12067200

Optimization using ωB97XD/6-31++G(d,p)[SDD]

- Energy = -1469.77668 au
- Zero-point energy correction = 0.254975 au
- Thermal correction to Gibbs free energy = 0.211038 au

Single point energy using ωB97XD/6-311++G(2df,2p)[SDD]

- Energy = -1469.9635667 au

Single point PCM acetonitrile solvent corrected energy using ωB97XD/6-311++G(2df,2p)[SDD]

- Energy = -1469.9833582 au

**S11:** Cartesian coordinates, energies, zero-point energy correction, thermal correction to Gibbs free energy, single point and solvent corrected energies for pathway **6** with S-coordination first.

**(1) Ligand**

C -0.41862200 0.01841900 0.58060800

C -0.75996700 2.53308600 -0.53230200

C -1.68854900 0.59363900 0.57381400

C -1.85234700 1.85827400 0.00204600

C 0.50519700 1.95290400 -0.51463200

C 0.68634800 0.68128600 0.03395800

C -2.86202600 -0.14944000 1.15750700

C -2.90929500 -1.87108300 -1.03006600

H -2.84025400 2.30962600 -0.02659200

H -0.89455700 3.51764600 -0.96925900

H 1.36311100 2.47463600 -0.92379000

H -2.51864100 -0.97601900 1.78756800

H -3.47051300 0.50978700 1.78318600

H -2.49123600 -2.65693300 -0.39456100

H -2.10042400 -1.29218700 -1.48276300

H -3.49960100 -2.33630700 -1.82186200

H -0.28912900 -0.95497000 1.04731000

C 2.03747200 0.05799300 0.03042600

C 2.20394400 -1.33249300 -0.00680400

N 3.08578500 0.89417800 0.04284700

C 3.48774400 -1.86200800 -0.00292700

H 1.34223500 -1.98862800 -0.06108200

C 4.31302300 0.37389400 0.04113600

C 4.57383200 -0.99335600 0.02712400

H 3.63754500 -2.93675900 -0.03500200

H 5.13018400 1.09119500 0.05329500

H 5.59439700 -1.36006900 0.03093400

S -4.02900900 -0.80168500 -0.09041500

Optimization using ωB97XD/6-31++G(d,p)[SDD]

- Energy = -955.9882138 au
- Zero-point energy correction = 0.229459 au
- Thermal correction to Gibbs free energy = 0.187078 au

Single point energy using ωB97XD/6-311++G(2df,2p)[SDD]

- Energy = -956.1501166 au

Single point PCM acetonitrile solvent corrected energy using ωB97XD/6-311++G(2df,2p)[SDD]

- Energy = -956.1591991 au

**(2) Int 1**

C 0.48633000 1.09850000 0.67823500

C 0.71789500 2.71018900 -1.57625200

C -0.58202400 1.94185500 0.31234300

C -0.45778100 2.73488800 -0.84245100

C 1.78550200 1.90037100 -1.18432300

C 1.67766600 1.08020300 -0.06762000

C -1.77861200 2.09524600 1.23063900

C -2.51889400 -0.25929200 2.68115100

H -1.27913200 3.37856300 -1.14274700

H 0.80839700 3.32363200 -2.46636100

H 2.70803600 1.88748200 -1.75341500

H -1.48013500 2.23862300 2.27202100

H -2.39890900 2.94214600 0.93183300

H -3.03824000 0.27135600 3.48132400

H -1.45184800 -0.33596800 2.89250000

H -2.93488400 -1.26134500 2.56220600

H 0.45132000 0.56336400 1.62424000

Pd -1.28670500 -0.38251800 -0.39063700

Cl 0.17179500 -1.17258300 -1.96437700

Cl -2.64119500 -2.20431500 -0.59044500

C 2.79637800 0.18687900 0.32254700

C 2.54953500 -1.09871200 0.81507500

N 4.03351900 0.67140500 0.15113400

C 3.63130600 -1.89513700 1.17054700

H 1.53619400 -1.48316300 0.86230700

C 5.06193900 -0.10576400 0.49305500

C 4.91774900 -1.38878400 1.01574900

H 3.47065400 -2.90117300 1.54492300

H 6.05010000 0.32153800 0.34130200

H 5.79208200 -1.97496300 1.27735500

S -2.81387400 0.58315800 1.10148100

Optimization using ωB97XD/6-31++G(d,p)[SDD]

- Energy = -2004.3276363 au
- Zero-point energy correction = 0.233554 au
- Thermal correction to Gibbs free energy = 0.183937 au

Single point energy using ωB97XD/6-311++G(2df,2p)[SDD]

- Energy = -2004.5706705 au

Single point PCM acetonitrile solvent corrected energy using ωB97XD/6-311++G(2df,2p)[SDD]

- Energy = -2004.6042585 au

**(3) TS 1-2**

C 0.13654600 1.15909600 0.18406300

C 1.11931400 3.78043700 -0.02530100

C -0.73884500 2.27226800 0.25515600

C -0.25099400 3.56642600 0.14020900

C 1.98047900 2.70332900 -0.15376400

C 1.49821000 1.38767600 -0.09524000

C -2.21835300 2.00391000 0.31783700

C -4.13801600 0.03548400 -0.37940400

H -0.93439700 4.41047700 0.16851500

H 1.50560800 4.79267100 -0.08959200

H 3.04191800 2.85667800 -0.31579300

H -2.53309400 1.63049200 1.29751300

H -2.81366400 2.88179500 0.05507900

H -4.90018500 0.77818900 -0.62358500

H -4.12596200 -0.19149800 0.68703200

H -4.30981900 -0.88924700 -0.93046600

H 0.29629500 0.13669000 1.24182500

Pd -0.91606800 -0.67066000 0.07383900

Cl 0.56115500 -1.22113300 1.96175900

Cl -2.09967100 -2.65786200 -0.18328700

C 2.46317100 0.27659300 -0.32497900

C 2.12053200 -0.82730400 -1.11104800

N 3.67402200 0.41702300 0.23093300

C 3.04943800 -1.84721300 -1.27728700

H 1.14583100 -0.88335200 -1.58658900

C 4.55793700 -0.56567900 0.06024000

C 4.29412900 -1.72207400 -0.67137700

H 2.80088500 -2.72256800 -1.86856200

H 5.52554300 -0.42158000 0.53459200

H 5.04991800 -2.49409800 -0.76597900

S -2.52590000 0.66573600 -0.91036000

Optimization using ωB97XD/6-31++G(d,p)[SDD]

- Energy = -2004.2826718 au
- Zero-point energy correction = 0.227200 au
- Thermal correction to Gibbs free energy = 0.179688 au

Single point energy using ωB97XD/6-311++G(2df,2p)[SDD]

- Energy = -2004.5279353 au

Single point PCM acetonitrile solvent corrected energy using ωB97XD/6-311++G(2df,2p)[SDD]

- Energy = -2004.5519429 au

**(4) Int 2**

C 0.14802600 1.11474200 0.27185900

C 1.29231300 3.69865700 0.67205800

C -0.64165100 2.28765300 0.35392300

C -0.08872600 3.54723100 0.56314700

C 2.10181800 2.59374800 0.47141000

C 1.54590300 1.31975600 0.24771500

C -2.12293200 2.13698400 0.11370200

C -3.98924200 0.30836200 -0.97465200

H -0.73546100 4.41842500 0.63119000

H 1.72976700 4.67207100 0.86615200

H 3.18188500 2.71183700 0.50099400

H -2.65234200 1.78288200 1.00375600

H -2.58605500 3.06196600 -0.24032800

H -4.64497500 1.09211700 -1.36008300

H -4.19878500 0.08656900 0.07232800

H -4.10294100 -0.61274300 -1.54665100

H 1.59548600 -1.20406800 0.99429300

Pd -0.94388300 -0.60210000 0.08963600

Cl 0.09655200 -1.68733800 1.95078800

Cl -2.31417600 -2.49526400 -0.33491100

C 2.50304000 0.26753500 -0.15751600

C 3.54259300 0.51211700 -1.06461800

N 2.38624800 -0.98525200 0.31323500

C 4.39413600 -0.51453300 -1.44215300

H 3.64475200 1.50229300 -1.49106800

C 3.18041300 -2.00560300 -0.04338700

C 4.21606800 -1.80283300 -0.92916700

H 5.18822600 -0.31936000 -2.15555500

H 2.94100500 -2.96114500 0.40882300

H 4.85685100 -2.62600800 -1.21876200

S -2.26834400 0.83186700 -1.17155200

Optimization using ωB97XD/6-31++G(d,p)[SDD]

- Energy = -2004.3356416 au
- Zero-point energy correction = 0.234369 au
- Thermal correction to Gibbs free energy = 0.187136 au

Single point energy using ωB97XD/6-311++G(2df,2p)[SDD]

- Energy = -2004.578567 au

Single point PCM acetonitrile solvent corrected energy using ωB97XD/6-311++G(2df,2p)[SDD]

- Energy = -2004.6158685 au

**(5) TS 2-3**

C -0.12213300 1.25301000 -0.00748800

C 0.58752000 3.96196000 0.16546400

C -1.11183400 2.24608500 0.05045500

C -0.75759800 3.59151500 0.13451000

C 1.57814000 2.99040300 0.08710200

C 1.22815000 1.63822600 -0.00602400

C -2.54204400 1.78113200 0.03235200

C -3.87768200 -0.72400900 -0.13158200

H -1.52828400 4.35655700 0.17880600

H 0.85891400 5.00915500 0.24710200

H 2.62495500 3.28141600 0.11420700

H -2.87872000 1.52165100 1.04121900

H -3.23192100 2.50958600 -0.40141800

H -4.84557600 -0.25481700 -0.31994800

H -3.66930600 -0.79448000 0.93671200

H -3.84227000 -1.72369100 -0.56667100

H 1.19188800 -0.69805300 1.44076700

Pd -0.62090500 -0.65643600 -0.18793600

Cl 0.25198400 -0.87288700 2.62543500

Cl -1.05528300 -2.95604500 -0.54734200

C 2.24688000 0.58096700 -0.17010900

C 3.39584900 0.72062600 -0.95009100

N 1.98402100 -0.61092100 0.41055500

C 4.23171600 -0.37311600 -1.13715200

H 3.60007500 1.66862600 -1.43415700

C 2.77293100 -1.67863800 0.22051900

C 3.91286300 -1.60244700 -0.55840400

H 5.12142200 -0.27459000 -1.75087200

H 2.45184300 -2.58610900 0.72223400

H 4.53216300 -2.47852500 -0.70633100

S -2.58201100 0.23208200 -0.96471000

Optimization using ωB97XD/6-31++G(d,p)[SDD]

- Energy = -2004.3142041 au
- Zero-point energy correction = 0.227857 au
- Thermal correction to Gibbs free energy = 0.179455 au

Single point energy using ωB97XD/6-311++G(2df,2p)[SDD]

- Energy = -2004.5558573 au

Single point PCM acetonitrile solvent corrected energy using ωB97XD/6-311++G(2df,2p)[SDD]

- Energy = -2004.5887336 au

**(6) Int 3**

C -0.01533500 1.22270500 -0.34562000

C 0.59621800 3.93497500 -0.28336900

C -1.04784500 2.16282400 -0.40028100

C -0.73352600 3.52374300 -0.38452800

C 1.62365400 2.99911400 -0.18598500

C 1.31959700 1.63643800 -0.21504900

C -2.46593600 1.65350300 -0.39229000

C -3.62615800 -0.89867700 0.17243400

H -1.52294500 4.26902300 -0.43830800

H 0.82998700 4.99445300 -0.26751500

H 2.64870800 3.34390600 -0.08472300

H -2.85983700 1.64962100 0.62979200

H -3.13743200 2.24007900 -1.02457200

H -4.64729500 -0.59219100 -0.06079100

H -3.36769000 -0.65702100 1.20466700

H -3.50165500 -1.97105700 0.01119900

H -0.74181200 -0.26022600 1.78580600

Pd -0.32524400 -0.70986800 -0.42292500

Cl -1.19202900 0.09111400 2.96363600

Cl -0.68190700 -3.07457400 -0.40989100

C 2.29187100 0.53791500 -0.08389800

C 3.66922700 0.67759300 0.07033600

N 1.73227900 -0.70096000 -0.10846700

C 4.46107900 -0.45757300 0.19985300

H 4.11501400 1.66506600 0.09021400

C 2.49023900 -1.79612000 0.01356500

C 3.86737500 -1.71549700 0.17245600

H 5.53470200 -0.35795300 0.32320100

H 1.95364000 -2.73970700 -0.02219700

H 4.45071100 -2.62311900 0.27089900

S -2.47779200 -0.09794100 -0.97770100

Optimization using ωB97XD/6-31++G(d,p)[SDD]

- Energy = -2004.355339 au
- Zero-point energy correction = 0.230241 au
- Thermal correction to Gibbs free energy = 0.181082 au

Single point energy using ωB97XD/6-311++G(2df,2p)[SDD]

- Energy = -2004.5973553 au

Single point PCM acetonitrile solvent corrected energy using ωB97XD/6-311++G(2df,2p)[SDD]

- Energy = -2004.6181962 au

**(7) Product**

C -0.14764700 1.22410400 -0.03141400

C 0.46522700 3.94324000 0.02014100

C -1.18051200 2.16715600 -0.01283000

C -0.86701300 3.52897900 -0.00065000

C 1.49733400 3.00728400 0.03356700

C 1.19333100 1.64469400 0.00751900

C -2.60215500 1.66764100 0.04530600

C -3.61107400 -0.86239300 0.90659100

H -1.65948600 4.27340600 -0.00085700

H 0.69805200 5.00307200 0.03204200

H 2.52691500 3.35267700 0.06405900

H -2.99632300 1.73345300 1.06467600

H -3.27260200 2.22478800 -0.61465100

H -4.65971700 -0.57154100 0.82236000

H -3.19677100 -0.57381800 1.87338000

H -3.50119400 -1.94088000 0.77776000

Pd -0.45561700 -0.70344600 -0.10330400

Cl -0.80405300 -3.07798600 -0.07284100

C 2.17789400 0.54933800 0.03936500

C 3.56311100 0.69360000 0.08566300

N 1.62248900 -0.69088300 0.03120000

C 4.36742200 -0.43963500 0.12321000

H 4.00607900 1.68258300 0.09140700

C 2.39208500 -1.78365400 0.06568500

C 3.77770800 -1.69965500 0.11396600

H 5.44735800 -0.33699500 0.16033800

H 1.85716400 -2.72890700 0.05229100

H 4.37082200 -2.60602900 0.14192700

S -2.65654400 -0.11472000 -0.44184600

Optimization using ωB97XD/6-31++G(d,p)[SDD]

- Energy = -1543.5595076 au
- Zero-point energy correction = 0.221609 au
- Thermal correction to Gibbs free energy = 0.177517 au

Single point energy using ωB97XD/6-311++G(2df,2p)[SDD]

- Energy = -1543.7665862 au

Single point PCM acetonitrile solvent corrected energy using ωB97XD/6-311++G(2df,2p)[SDD]

- Energy = -1543.7879507 au

**S12:** Cartesian coordinates, energies, zero-point energy correction, thermal correction to Gibbs free energy, single point and solvent corrected energies for pathway **6** with N-coordination first.

**(1) Ligand**

C -0.41633200 -0.04545100 -0.57007500

C -0.83487200 2.61185900 0.10709900

C 0.66716500 0.73132100 -0.14417000

C 0.44652300 2.07028000 0.18684900

C -1.90512000 1.82404400 -0.29881500

C -1.70212500 0.48481000 -0.64771000

C -2.85665200 -0.38507500 -1.07187300

C -2.76607000 -1.77103900 1.34288700

H 1.27551200 2.70610100 0.48227800

H -0.99488400 3.65644500 0.35507000

H -2.90569000 2.24517400 -0.34436400

H -3.51122400 0.14801900 -1.76724300

H -2.49281500 -1.28285300 -1.58054300

H -2.32705700 -2.61836300 0.80920800

H -1.97347400 -1.10277100 1.68773000

H -3.31608900 -2.14656400 2.20792700

H -0.23538900 -1.08096300 -0.84221300

C 2.02163400 0.12154700 -0.05634800

C 2.97437000 0.58614600 0.85968800

N 2.27682400 -0.90299900 -0.88299200

C 4.22790700 -0.01033300 0.89416100

H 2.72805400 1.38538800 1.54980800

C 3.48256500 -1.46979700 -0.83713000

C 4.49779300 -1.06054900 0.02262000

H 4.97881100 0.33342700 1.59872300

H 3.64490900 -2.29295300 -1.52895900

H 5.46188700 -1.55674300 0.01161900

S -3.95569000 -0.88743300 0.30117500

Optimization using ωB97XD/6-31++G(d,p)[SDD]

- Energy = -955.9885048 au
- Zero-point energy correction = 0.229277 au
- Thermal correction to Gibbs free energy = 0.186867 au

Single point energy using ωB97XD/6-311++G(2df,2p)[SDD]

- Energy = -956.150481 au

Single point PCM acetonitrile solvent corrected energy using ωB97XD/6-311++G(2df,2p)[SDD]

- Energy = -956.1592757 au

**(2) Int 1**

C 0.90924800 0.65313100 -0.09513200

C 1.65036800 1.37985700 2.48229500

C 0.04913200 1.45374100 0.69024200

C 0.42909500 1.82241700 1.97956900

C 2.48739100 0.57089100 1.71838400

C 2.13424900 0.20165900 0.42144300

C 3.04127200 -0.64788200 -0.42310700

C 3.80374000 1.51940800 -1.98244500

H -0.24069800 2.41279400 2.59664600

H 1.94087700 1.64803100 3.49305000

H 3.43114600 0.22697400 2.13001800

H 3.38026200 -1.51367700 0.15142900

H 2.50638900 -1.03703700 -1.29325300

H 3.21735900 1.08520100 -2.79716700

H 3.17467600 2.18466000 -1.38489300

H 4.62176400 2.10102600 -2.41187300

H 0.70050300 0.50612400 -1.15582800

Pd -0.85661400 -1.01505700 0.01274900

Cl 0.49442900 -2.78409700 0.50174000

Cl -2.66239300 -2.36330100 -0.31317500

C -1.28765600 1.79239900 0.14355600

C -1.85901700 3.05847300 0.13263000

N -1.94314800 0.72064800 -0.34364800

C -3.12992400 3.20857100 -0.41561500

H -1.30919900 3.90249900 0.53358500

C -3.16663500 0.85482600 -0.86660000

C -3.78989900 2.09487600 -0.92698900

H -3.59895500 4.18651600 -0.44688900

H -3.63140300 -0.06214000 -1.21318300

H -4.77845100 2.17662300 -1.36300800

S 4.55120000 0.22262700 -0.96090900

Optimization using ωB97XD/6-31++G(d,p)[SDD]

- Energy = -2004.337731 au
- Zero-point energy correction = 0.233930 au
- Thermal correction to Gibbs free energy = 0.183642 au

Single point energy using ωB97XD/6-311++G(2df,2p)[SDD]

- Energy = -2004.5784914 au

Single point PCM acetonitrile solvent corrected energy using ωB97XD/6-311++G(2df,2p)[SDD]

- Energy = -2004.6095188 au

**(3) TS 1-2**

C -0.51400300 0.72316500 -0.06939400

C -1.46567900 3.36791300 -0.08563000

C 0.35776100 1.81703300 0.19387800

C -0.12421500 3.12201500 0.19817500

C -2.29243300 2.32176900 -0.45739300

C -1.82940800 0.99605800 -0.48714600

C -2.73759800 -0.07135600 -1.06993800

C -4.35167100 -0.22976800 1.21184200

H 0.54299800 3.95919300 0.37436400

H -1.84773500 4.38345900 -0.06872500

H -3.32336100 2.51397100 -0.73948000

H -2.75796500 0.06878700 -2.15632800

H -2.34970900 -1.07419100 -0.89473500

H -3.99173300 -1.23113300 1.45200800

H -3.68660200 0.51755100 1.65375500

H -5.35337000 -0.09711100 1.62566200

H -0.82266500 -0.51672500 0.80785900

Pd 0.61722100 -1.03164300 -0.13783800

Cl -1.17847200 -1.99275500 1.19023700

Cl 1.95282900 -2.91819000 -0.42131100

C 1.80688300 1.54371500 0.22270500

C 2.79186100 2.46686600 0.57192600

N 2.16297600 0.31185900 -0.21861600

C 4.13115800 2.13145900 0.42375000

H 2.51157100 3.43775400 0.96172900

C 3.45313900 -0.01825400 -0.36750800

C 4.47030000 0.87689900 -0.07212800

H 4.90225000 2.84501300 0.69498700

H 3.63964500 -1.03085900 -0.70769200

H 5.50260300 0.57898100 -0.21051700

S -4.48997100 0.00743000 -0.57953700

Optimization using ωB97XD/6-31++G(d,p)[SDD]

- Energy = -2004.2804731 au
- Zero-point energy correction = 0.227318 au
- Thermal correction to Gibbs free energy = 0.179092 au

Single point energy using ωB97XD/6-311++G(2df,2p)[SDD]

- Energy = -2004.5241771 au

Single point PCM acetonitrile solvent corrected energy using ωB97XD/6-311++G(2df,2p)[SDD]

- Energy = -2004.5462581 au

**(4) Int 2**

C -0.42994800 0.92450800 -0.32040900

C -1.04011500 3.71236500 -0.32205500

C 0.55008900 1.91807600 -0.06138800

C 0.24907400 3.28188400 -0.04467200

C -2.00245700 2.76692500 -0.64572800

C -1.71534800 1.39402400 -0.65687800

C -2.84879800 0.49435200 -1.06115100

C -3.16853600 0.64235600 1.81965700

H 1.01729800 4.01833200 0.16753800

H -1.28345500 4.76957400 -0.31452400

H -3.00729200 3.09504600 -0.90328300

H -3.53993900 1.02754800 -1.71531400

H -2.48684800 -0.38772200 -1.59317500

H -2.11314400 0.38915400 1.93410100

H -3.28913500 1.72495800 1.77077800

H -3.73602000 0.24980600 2.66560600

H -2.70488600 -1.55989700 0.29783800

Pd 0.33174500 -0.96125300 -0.17298100

Cl -1.61992000 -2.39717500 0.02973800

Cl 1.45445800 -3.07170200 -0.08100400

C 1.92682800 1.43885800 0.11479500

C 3.04351600 2.23774800 0.36494300

N 2.06959900 0.10083600 -0.02691200

C 4.30072900 1.65691800 0.44832600

H 2.92868000 3.30669100 0.49237600

C 3.28329600 -0.46665900 0.04664600

C 4.42671500 0.28258800 0.27701400

H 5.17312300 2.27234500 0.64352200

H 3.30026300 -1.54446900 -0.07295100

H 5.38816000 -0.21391800 0.32817000

S -3.87698000 -0.14802700 0.34760800

Optimization using ωB97XD/6-31++G(d,p)[SDD]

- Energy = -2004.3164203 au
- Zero-point energy correction = 0.230682 au
- Thermal correction to Gibbs free energy = 0.182449 au

Single point energy using ωB97XD/6-311++G(2df,2p)[SDD]

- Energy = -2004.5586088 au

Single point PCM acetonitrile solvent corrected energy using ωB97XD/6-311++G(2df,2p)[SDD]

- Energy = -2004.5803502 au

**(5) TS 2-3**

C 0.74256100 1.22371300 -0.16284700

C 2.62284200 3.21459900 -0.68152300

C 2.10460600 0.89584800 -0.27277200

C 3.04479000 1.89540100 -0.53224700

C 1.26897800 3.53973200 -0.59044900

C 0.32044500 2.54517300 -0.33892800

C -1.15965500 2.82462200 -0.31668400

C -2.05684000 1.83924000 2.16597200

H 4.10226200 1.66298700 -0.61895700

H 3.35208200 3.99414200 -0.87615600

H 0.95566400 4.57226100 -0.72475400

H -1.42538300 3.73031400 0.23732800

H -1.54990100 2.93280400 -1.33362700

H -1.03881200 2.07668500 2.47788600

H -2.72829400 2.68077800 2.34777100

H -2.41043000 0.95872200 2.70456900

H -2.82192600 -1.77789600 -0.99440700

Pd -0.46947100 -0.23820300 0.28970700

Cl -3.25679900 -1.46991400 -2.19691600

Cl -1.92435600 -2.05916200 0.93989300

C 2.40599400 -0.53594300 -0.10520000

C 3.66484300 -1.12480200 -0.20256900

N 1.31431300 -1.30189000 0.15593600

C 3.79281200 -2.49814100 -0.03105200

H 4.53429800 -0.51360300 -0.41445600

C 1.43330600 -2.62335200 0.32333600

C 2.66232500 -3.26366700 0.23678000

H 4.76867400 -2.96667800 -0.10846100

H 0.50798600 -3.15158000 0.53173100

H 2.72232200 -4.33633800 0.37648200

S -2.09846900 1.41298300 0.39971500

Optimization using ωB97XD/6-31++G(d,p)[SDD]

- Energy = -2004.3579111 au
- Zero-point energy correction = 0.230516 au
- Thermal correction to Gibbs free energy = 0.180381 au

Single point energy using ωB97XD/6-311++G(2df,2p)[SDD]

- Energy = -2004.6002066 au

Single point PCM acetonitrile solvent corrected energy using ωB97XD/6-311++G(2df,2p)[SDD]

- Energy = -2004.5687624 au

**(6) Int 3**

C 0.74256100 1.22371300 -0.16284700

C 2.62284200 3.21459900 -0.68152300

C 2.10460600 0.89584800 -0.27277200

C 3.04479000 1.89540100 -0.53224700

C 1.26897800 3.53973200 -0.59044900

C 0.32044500 2.54517300 -0.33892800

C -1.15965500 2.82462200 -0.31668400

C -2.05684000 1.83924000 2.16597200

H 4.10226200 1.66298700 -0.61895700

H 3.35208200 3.99414200 -0.87615600

H 0.95566400 4.57226100 -0.72475400

H -1.42538300 3.73031400 0.23732800

H -1.54990100 2.93280400 -1.33362700

H -1.03881200 2.07668500 2.47788600

H -2.72829400 2.68077800 2.34777100

H -2.41043000 0.95872200 2.70456900

H -2.82192600 -1.77789600 -0.99440700

Pd -0.46947100 -0.23820300 0.28970700

Cl -3.25679900 -1.46991400 -2.19691600

Cl -1.92435600 -2.05916200 0.93989300

C 2.40599400 -0.53594300 -0.10520000

C 3.66484300 -1.12480200 -0.20256900

N 1.31431300 -1.30189000 0.15593600

C 3.79281200 -2.49814100 -0.03105200

H 4.53429800 -0.51360300 -0.41445600

C 1.43330600 -2.62335200 0.32333600

C 2.66232500 -3.26366700 0.23678000

H 4.76867400 -2.96667800 -0.10846100

H 0.50798600 -3.15158000 0.53173100

H 2.72232200 -4.33633800 0.37648200

S -2.09846900 1.41298300 0.39971500

Optimization using ωB97XD/6-31++G(d,p)[SDD]

- Energy = -2004.3579111 au
- Zero-point energy correction = 0.230516 au
- Thermal correction to Gibbs free energy = 0.180381 au

Single point energy using ωB97XD/6-311++G(2df,2p)[SDD]

- Energy = -2004.6002066 au

Single point PCM acetonitrile solvent corrected energy using ωB97XD/6-311++G(2df,2p)[SDD]

- Energy = -2004.6209195 au

**(7) Product**

C -0.30576900 1.17339400 -0.12772000

C -0.02181600 3.94093200 0.08726400

C 0.97076100 1.74506700 0.01996400

C 1.11086200 3.13031000 0.12577200

C -1.28962400 3.37824800 -0.06210500

C -1.43845900 1.99298200 -0.17272400

C -2.78357700 1.34651200 -0.38615700

C -3.15719900 -0.77662900 1.45060400

H 2.08854700 3.58875800 0.24406700

H 0.08327100 5.01728400 0.17599500

H -2.16242700 4.02642000 -0.09410700

H -3.53928200 1.68271200 0.33062200

H -3.16653600 1.55705500 -1.38961500

H -2.57412200 -0.13926100 2.11651100

H -4.22683500 -0.59107900 1.56673300

H -2.93226300 -1.82445400 1.65692500

Pd -0.38353100 -0.77863500 -0.17936200

Cl -0.44018100 -3.17236700 -0.07176000

C 2.07632700 0.77243900 0.05983500

C 3.43064400 1.07604100 0.18430900

N 1.67580800 -0.52249300 -0.03435900

C 4.36302800 0.04561300 0.20977400

H 3.74925600 2.10906300 0.26152500

C 2.56862800 -1.51758800 -0.00858800

C 3.93052300 -1.27336400 0.11159000

H 5.42009900 0.27134800 0.30839700

H 2.14840000 -2.51665200 -0.08209500

H 4.62773700 -2.10252600 0.12861800

S -2.67793700 -0.49234400 -0.27896300

Optimization using ωB97XD/6-31++G(d,p)[SDD]

- Energy = -2004.3579111 au
- Zero-point energy correction = 0.230516 au
- Thermal correction to Gibbs free energy = 0.180381 au

Single point energy using ωB97XD/6-311++G(2df,2p)[SDD]

- Energy = -2004.6002066 au

Single point PCM acetonitrile solvent corrected energy using ωB97XD/6-311++G(2df,2p)[SDD]

- Energy = -1543.7883798 au

**S13:** Cartesian coordinates, energies, zero-point energy correction, thermal correction to Gibbs free energy, single point and solvent corrected energies for HCl.

Cl 0.00000000 0.00000000 0.07121700

H 0.00000000 0.00000000 -1.21068500

Optimization using ωB97XD/6-31++G(d,p)[SDD]

- Energy = -460.7846958 au
- Zero-point energy correction = 0.006812 au
- Thermal correction to Gibbs free energy = -0.011069 au

Single point energy using ωB97XD/6-311++G(2df,2p)[SDD]

- Energy = -460.8185934 au

Single point PCM acetonitrile solvent corrected energy using ωB97XD/6-311++G(2df,2p)[SDD]

- Energy = -460.8214359 au

**S14:** Cartesian coordinates, energies, zero-point energy correction, thermal correction to Gibbs free energy, single point and solvent corrected energies for PdCl_2_.

Pd 0.00000000 0.00000000 0.61281800

Cl 0.00000000 1.66150000 -0.82910600

Cl 0.00000000 -1.66150000 -0.82910600

Optimization using ωB97XD/6-31++G(d,p)[SDD]

- Energy = -1048.2719833 au
- Zero-point energy correction = 0.002065 au
- Thermal correction to Gibbs free energy = -0.027061 au

Single point energy using ωB97XD/6-311++G(2df,2p)[SDD]

- Energy = -1048.3428062 au

Single point PCM acetonitrile solvent corrected energy using ωB97XD/6-311++G(2df,2p)[SDD]

- Energy = -1048.3863304 au
